# The presence of *Pseudogymnoascus destructans*, a fungal pathogen of bats, correlates with changes in microbial metacommunity structure

**DOI:** 10.1038/s41598-021-91118-1

**Published:** 2021-06-03

**Authors:** Matthew Grisnik, Joshua B. Grinath, Donald M. Walker

**Affiliations:** 1 Department of Biology, Middle Tennessee State University, 1672 Greenland Drive, Murfreesboro, TN 37132 USA; 2 Department of Biological Sciences, Idaho State University, Pocatello, ID 83209 USA

**Keywords:** Community ecology, Ecology, Microbial ecology, Microbiome

## Abstract

Metacommunity theory provides a framework for how community patterns arise from processes across scales, which is relevant for understanding patterns in host-associated microbial assemblages. Microbial metacommunities may have important roles in host health through interactions with pathogens; however, it is unclear how pathogens affect host microbial metacommunities. Here, we studied relationships between a fungal pathogen and a host-associated microbial metacommunity. We hypothesized that a fungal pathogen of bats, *Pseudogymnoascus destructans,* correlates with a shift in metacommunity structure and changes in relationships between community composition, and factors shaping these assemblages, such as ecoregion. We sampled bat cutaneous microbial assemblages in the presence/absence of *P. destructans* and analyzed microbial metacommunity composition and relationships with structuring variables. Absence of *P. destructans* correlated with a metacommunity characterized by a common core microbial group that was lacking in disease positive bats. Additionally, *P. destructans* presence correlated with a change in the relationship between community structure and ecoregion. Our results suggest that the fungal pathogen intensifies local processes influencing a microbial metacommunity and highlights the importance of cutaneous microbial assemblages in host–pathogen interactions.

## Introduction

Elucidating how patterns of community structure relate to underlying structuring variables and processes of community assembly is a primary goal of community ecologists. Patterns observed at one scale of observation can be directly influenced by processes occurring at another scale^1^. For example, rescue effects describe the process by which species can persist in unfavorable local environments through dispersal from regional source populations^2^. Communities interacting between scales, including local and regional, form a spatial patchwork of taxa referred to as a metacommunity^3^.

Metacommunities are defined as groups of habitat patches, linked by species dispersal and interactions between taxa among these patches^3,4^. Both local and regional processes contribute to shaping metacommunity structure and the distribution of species across habitat patches^3^. By using pattern-based assessments, one can analyze species distributions along environmental and spatial gradients to diagnose metacommunity structure^5,6^.

**Figure 1:**
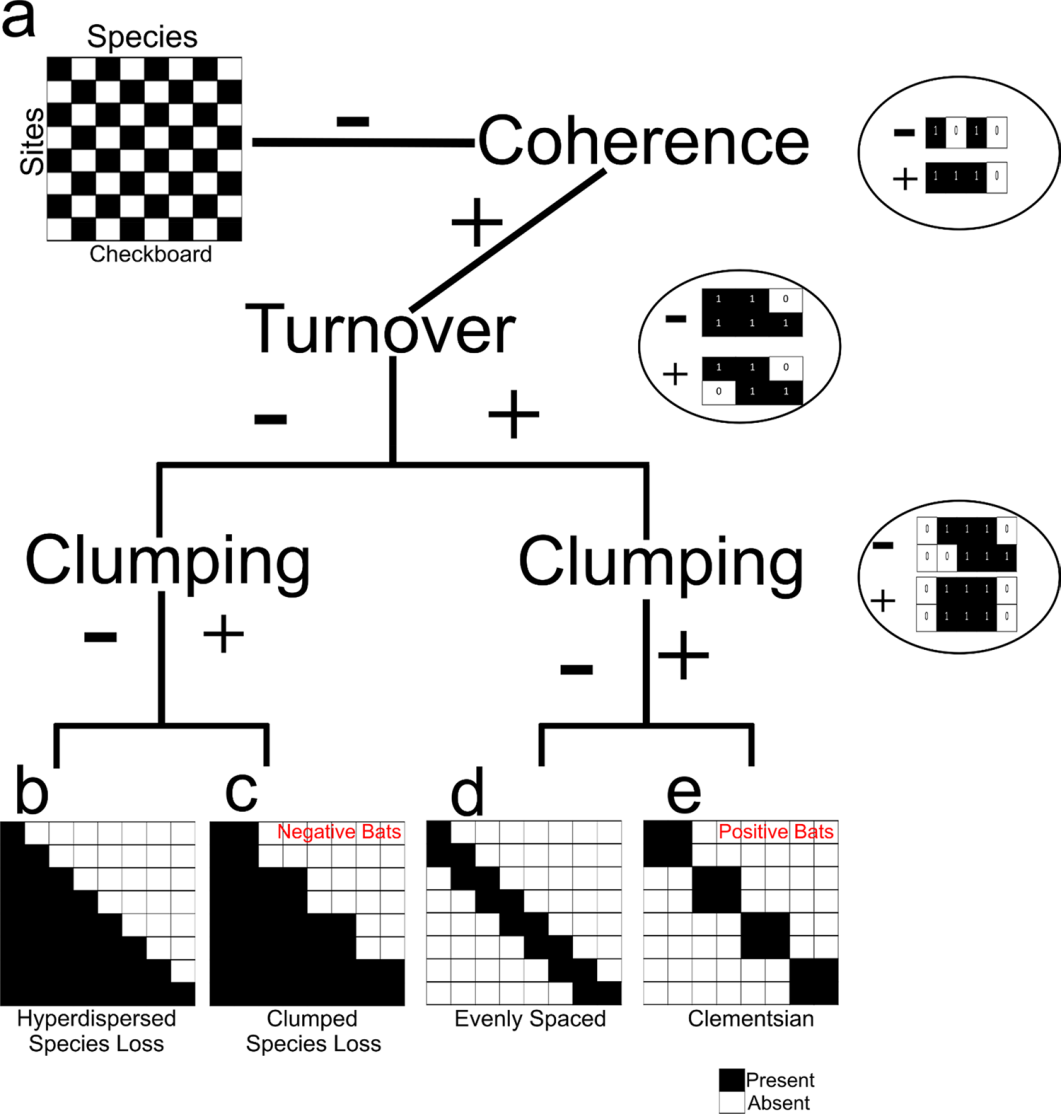
The elements of metacommunity structure and their resulting patterns. Plus signs ( +) indicate a significantly positive relationship, whereas minus signs ( −) indicate a negative relationship. (**a**) metacommunity with checkerboard pattern. (**b**) nested metacommunity with hyperdispersed species loss. (**c**) nested metacommunity with clumped species loss. (**d**) evenly spaced metacommunity. (**e**) Clementsian metacommunity structure. (Modified from reference^5^ using the software Inkscape 1.0 www.inkscape.org).

Leibold and Mikkelson^6^ developed a framework to identify metacommunity patterns, called the Elements of Metacommunity Structure (EMS), which uses three metrics to describe metacommunity structures: coherence, turnover, and boundary clumping (Fig. 1). Coherence is measured as the number of embedded species absences from a site and describes the overall response of a community to an environmental or spatial gradient (Fig. 1a). Turnover is measured as the number of species replacements across samples. Boundary clumping describes clustering in species’ range boundaries and is a metric that defines how cohesive species ranges are across sites^6^. After determining the EMS, an idealized distributional pattern including hyperdispersed species loss, clumped species loss, evenly spaced, or Clementsian (Fig. 1b-e) can be used to describe metacommunity structure^5,6^. Clementsian structure (Fig. 1e) describes communities of species that have similar responses to environmental differences, resulting in discrete community boundaries^5–7^. Specifically, Clementsian metacommunities have positive coherence, turnover, and clumping, meaning there are less absences (positive coherence) but more frequent species replacements (positive turnover) than expected by chance alone. Additionally, Clementsian metacommunities have clumped species boundaries defined by positive boundary clumping. For example, Clementsian succession occurs when communities of species replace each other over time, with little overlap in community composition. Evenly spaced metacommunities (Fig. 1d) are similar to Clementsian, in that they have positive coherence and turnover, but they have hyperdispersed species boundaries as opposed to clumped species loss. Evenly spaced metacommunities still exhibit turnover, with species replacing each other across sites, however there are no distinct communities characteristic of Clementsian metacommunities. Nested patterns consist of less diverse assemblages making up subsets of more diverse communities^5,6,8^. Nested community structures result from positive coherence, but negative turnover, where species do not replace each other but rather, are lost from sites. For example, nested sites are made up of subsets of species from a much larger species pool. Nested metacommunities that exhibit positive clumping have clumped species loss (Fig. 1c) where species are lost from sites in groups. Those with negative clumping have hyperdispersed loss (Fig. 1b) where individual species are lost from sites. While EMS analyses provide descriptions of metacommunity structure, they do not reveal the variables responsible for such processes.

Complimentary analyses are needed to elucidate the variables driving metacommunity structure. The influence of geographic distance on community structure is assessed using distance-decay models, which estimate the rate of species turnover along a gradient^9^. Positive relationships between community dissimilarity and geographic distance may indicate that species’ distributions are highly affected by dispersal limitation^10^, whereas, the lack of a relationship suggests that environmental filtering and species sorting may be more important for determining community structure^11^. Species sorting and environmental filtering emphasize the role of the local abiotic environment in determining what species can persist within an assemblage, resulting in assemblages correlating with local habitat factors, such as precipitation^3^. In addition, permutational multivariate methods are frequently implemented to understand the effects of environmental factors on community patterns. For instance, permutational models can distinguish differences in community composition and turnover across environmental variables, as well as the interactive effects of multiple environmental variables^12^. Interactive effects are especially important to consider when new structuring variables, such as invasive species or anthropogenic perturbations, are introduced to a metacommunity, as they might change the role of established structuring factors.

While macroorganismal metacommunities have been studied in some detail^10,13,14^, minimal work has focused on characterization of host-associated microbial metacommunities^15–17^. Understanding variation in structure of host-associated microbial metacommunities may be especially important given the role of host assemblages in pathogen defense^18,19^. For instance, the pathogenic fungus *Pseudogymnoascus destructans*, the causative agent of white-nose syndrome, was introduced into the United States in 2006, and is responsible for massive bat population declines^20,21^. Recently, declines have been shown to be highly variable across space, bat species, and time^22^. Tri-colored bats (*Perimyotis subflavus*) have shown recent population stabilizations possibly due in part to the presence of antifungal bacteria composing the cutaneous microbial assemblage^22,23^. Previous work has shown *P. subflavus* that are exposed to, but not invaded by *P. destructans,* have microbial assemblages enriched in antifungal bacterial taxa^24^. Determining the structure and drivers of bat cutaneous microbial metacommunities in relation to this fungal pathogen may improve our understanding of microbial metacommunity response to fungal invasion, as well as our understanding of assemblages that are resistant to fungal invasion.

Understanding how the presence of a fungal pathogen correlates with changes in both metacommunity structure and its relationship with structuring factors is likely important to understand the role of the host associated microbial assemblage in pathogen defense. The objectives of this study were to understand how the presence of a fungal pathogen correlates with the composition of a host-associated cutaneous microbial metacommunity and its structuring factors. We investigated the relationship between *P. destructans* and the cutaneous microbial metacommunity of hibernating *P. subflavus* across 48 sites in Tennessee, USA. We hypothesized that the presence of *P. destructans* would be correlated with a shift in 1) metacommunity structure and 2) relationships between structuring variables and community composition. We tested the first hypothesis using EMS, further informed by indicator operational taxonomic unit (OTU) and fungal pathogen load analyses. We tested the second hypothesis with distance-decay and permutational models to understand how spatial and environmental variables structure the bat cutaneous microbial assemblage in the presence/absence of a fungal pathogen.

## Results

**Figure 2:**
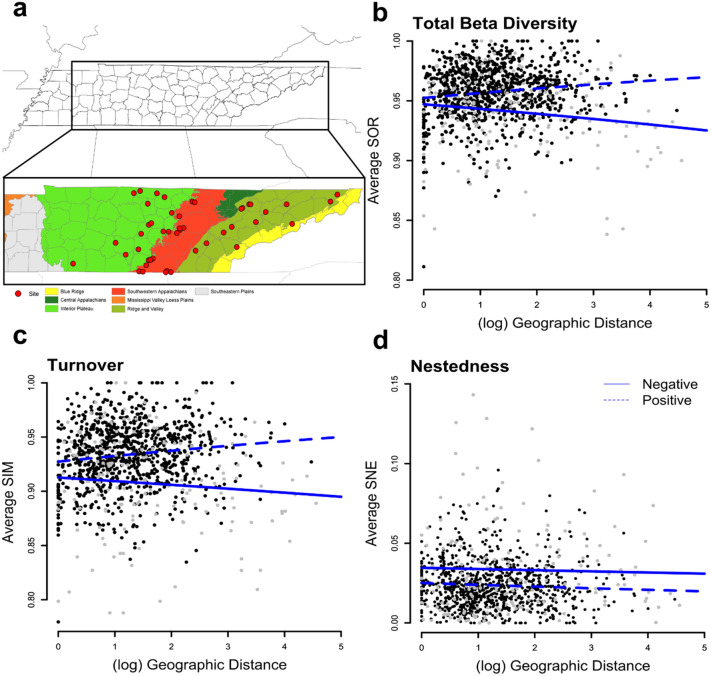
(**a**) Map of the study system, red dots indicate sample sites. Samples were collected across three Tennessee ecoregions (Interior Plateau in light green, South West Appalachians in red, and Ridge and Valley in olive green). Map produced using ArcGis 10.7.1. (https://desktop.arcgis.com/en/arcmap/) Copyright 1995–2018 Esri. All rights reserved. Published in the United States of America. Distance-decay relationships, comparing geographic distances between sites and (**b**) total beta diversity, (**c**) turnover, (**d**) nestedness, for *P. destructans* positive (dashed line and black dots) and *P. destructans* negative (solid line and grey dots) bats averaged by site. There is no significant relationship between geographic distance and community dissimilarity (GLM; SOR: z = 0.79, *p* > 0.05; SIM: z = 0.86, *p* > 0.05; SNE: z = 0.96, *p* > 0.05) or decay rates between disease states (*p* > 0.05) for any metric (ANOVA; SOR: *p* > 0.05, SIM: *p* > 0.05, SNE: *p* > 0.05). Graphs were produced using the ggplot2 package (version 3.3.2) in R^25^.

Of the 249 individuals of *P. subflavus* studied, quantitative PCR (qPCR) results indicated that there were 40 negative and 209 *P. destructans* positive bat individuals collected from 48 sites across three ecoregions (Interior, Ridge and Valley, and the South West Appalachians; Fig. 2a). All sites were determined to have at least one *P. destructans* positive bat. Post processing of high-throughput sequence data resulted in a mean read depth of 194,550 sequences per sample (14,899–2,968,637) reads and a total of 11,071 OTUs for *P. destructans* positive and 3370 OTUs for *P. destructans* negative bats.

### Metacommunity structure

**Figure 3:**
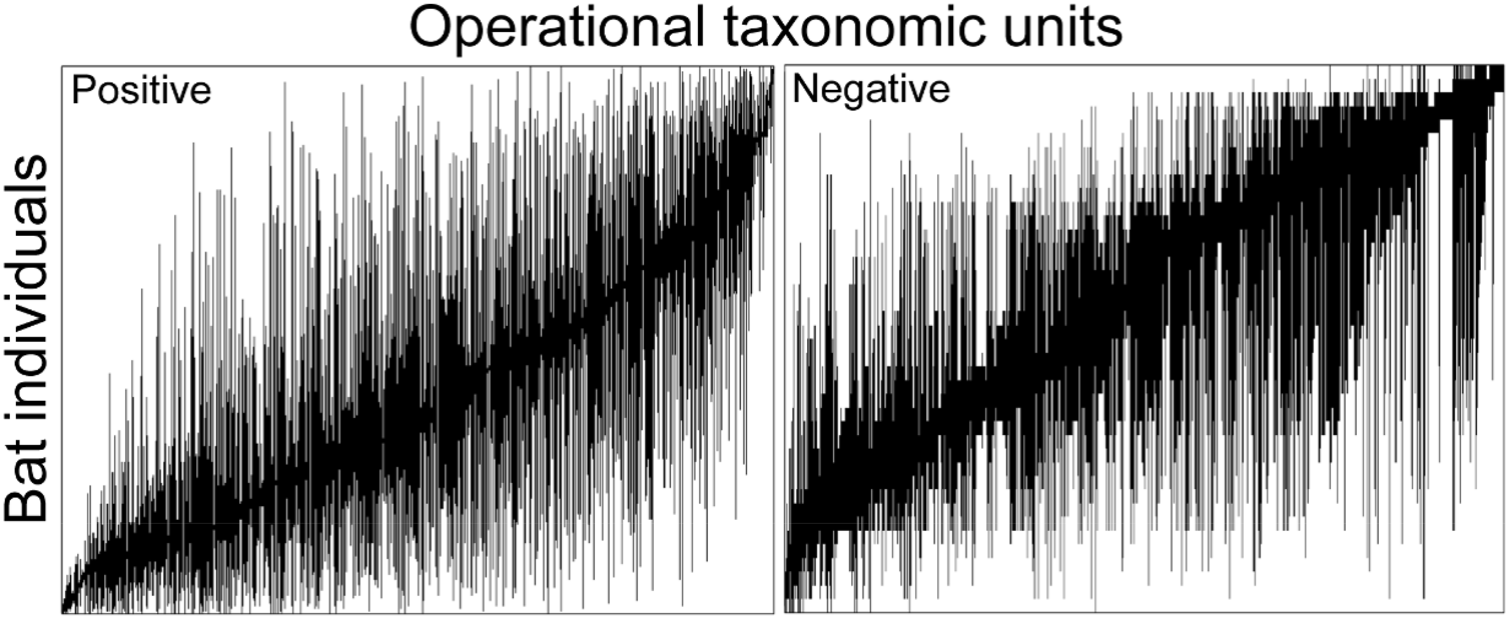
Site by species incidence matrix for OTUs on *P. destructans* positive/negative bats describing the actual metacommunity patterns. Black bars represent each OTUs (x-axis) range across bat samples (y-axis). EMS analysis suggests a Clementsian structure for *P. destructans* positive and a nested structure for *P. destructans* negative bat microbial metacommunities. Plots were produced using the *Imagine* function in the *metacom* package (version 1.5.3) in R^26^.

Metacommunities for both *P. destructans* positive and negative bats showed positive coherence, with significantly (*p* ≤ 0.05) less embedded absences than expected based on null models (positive = 1,142,539 embedded absences, 1,376,131.98 ± 1943.23 expected; negative = 46,167 embedded absences, 52,651 ± 186.4 expected; Fig. 3, Table 1). The *P. destructans* positive metacommunity was characterized by significant positive turnover (*p* ≤ 0.05, 2.48e + 10 replacements; simulated mean 2.26e + 10 ± 2.25e + 08), while the *P. destructans* negative metacommunity had significant negative turnover (*p* ≤ 0.05, 42,579,240 replacements; 47,012,450 ± 1,005,206 expected replacements). Both *P. destructans* positive and negative metacommunities had significant clumping of species range boundaries (positive bats; Morisita’s index = 1.44, *p* ≤ 0.05; negative bats; Morisita’s index = 1.39, *p* ≤ 0.05). Together, these results indicate that the *P. destructans* positive metacommunity can be described as having a Clementsian structure (Fig. 1e), whereas, the *P. destructans* negative metacommunity had a nested structure with clumped species losses (Fig. 1c; ref.^5^).

**Table 1:** Results for the EMS analysis of bats across *P. destructans* status.

*P. destructans* positive bats		
	Coherence	*p* value
Absences	1,142,539	≤ 0.0001
Simulated mean	1,376,131.9 (± 1943.2)	
	**Turnover**	
Turnover	2.48 e + 10	≤ 0.0001
Simulated mean	2.26 e + 10 (± 2.25 e + 8)	
	**Boundary**	
Index	1.44	≤ 0.0001

***P. destructans* negative bats**		
	Coherence	*p* value
Absences	46,167	≤ 0.0001
Simulated mean	52,651 (± 186.4)	
	**Turnover**	
Turnover	42,579,240	≤ 0.0001
Simulated mean	47,012,450 (± 1,005,206)	
	**Boundary**	
Index	1.39	≤ 0.0001

Results suggest Clementsian metacommunity structure for *P. destructans* positive bats, and a nested metacommunity structure for *P. destructans* negative bats^5^.

**Figure 4:**
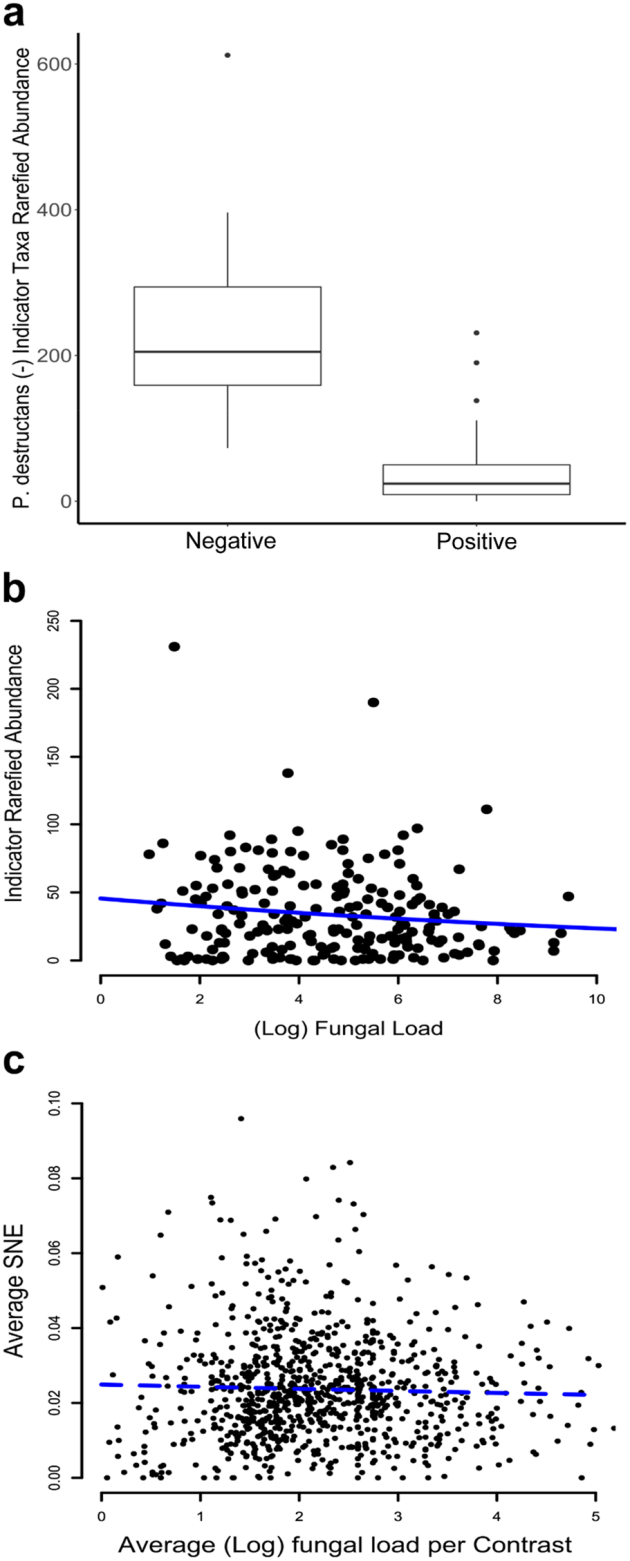
(**a**) Comparison of the rarefied abundance of *P. destructans* negative indicator taxa between *P. destructans* positive*/*negative samples. Indicator taxa are significantly more abundant within *P. destructans* negative samples (GLMM; z = − 62.84, *p* ≤ 0.05). (**b**) Comparison of the rarefied abundance of *P. destructans* negative indicator taxa by fungal load. There is a significantly negative relationship between indicator taxa rarefied abundance and amount of *P. destructans* present (GLMM; z = − 10.78, *p* ≤ 0.05). (**c**) Comparison of the (log) difference in average fungal load and average nestedness (SNE) of bats averaged by site. There is no significant relationship between similarity in fungal load and nestedness (GLM; z = − 0.10, *p* > 0.05). Graphs were produced using the *ggplot2* package (version 3.3.2) in R^25^.

A total of 14 OTUs were identified as indicator taxa for *P. destructans* positive and 363 OTUs for negative bats (Supplemental Table). The group of indicator OTUs for the *P. destructans* negative bats represents the common taxa occurring across the individual microbial communities that contributed to the nestedness in metacommunity structure. OTUs indicative of *P. destructans* negative bats were significantly more abundant on *P. destructans* negative, relative to *P. destructans* positive bats (GLMM; z = − 62.84, *p* ≤ 0.05; Fig. 4a, Supplemental File 1E). Additionally, there was a significant negative relationship between log transformed fungal load and indicator taxa rarefied abundance (GLMM; z = − 10.78, *p* ≤ 0.05; Fig. 4b, Supplemental File 1E), with increased fungal load predictive of fewer indicator taxa. However, similar patterns were not found when analyzing the relationship between *P. destructans* load and the nested component (SNE) of averaged community dissimilarities (GLM; z = − 0.10, *p* > 0.05; Fig. 4c). Between site average community dissimilarity (SOR and SIM) was not related to the between site average difference in log transformed fungal load (GLM; SOR: z = 0.237, *p* > 0.05; SIM: z = 0.798, *p*  > 0.05, Supplemental File 1E). When the compositional nature of the dataset was considered, patterns of indicator taxa abundance were identical to those observed with subsampled data (Supplemental File 1F).

### Relationship between community structure and structuring variables

**Figure 5:**
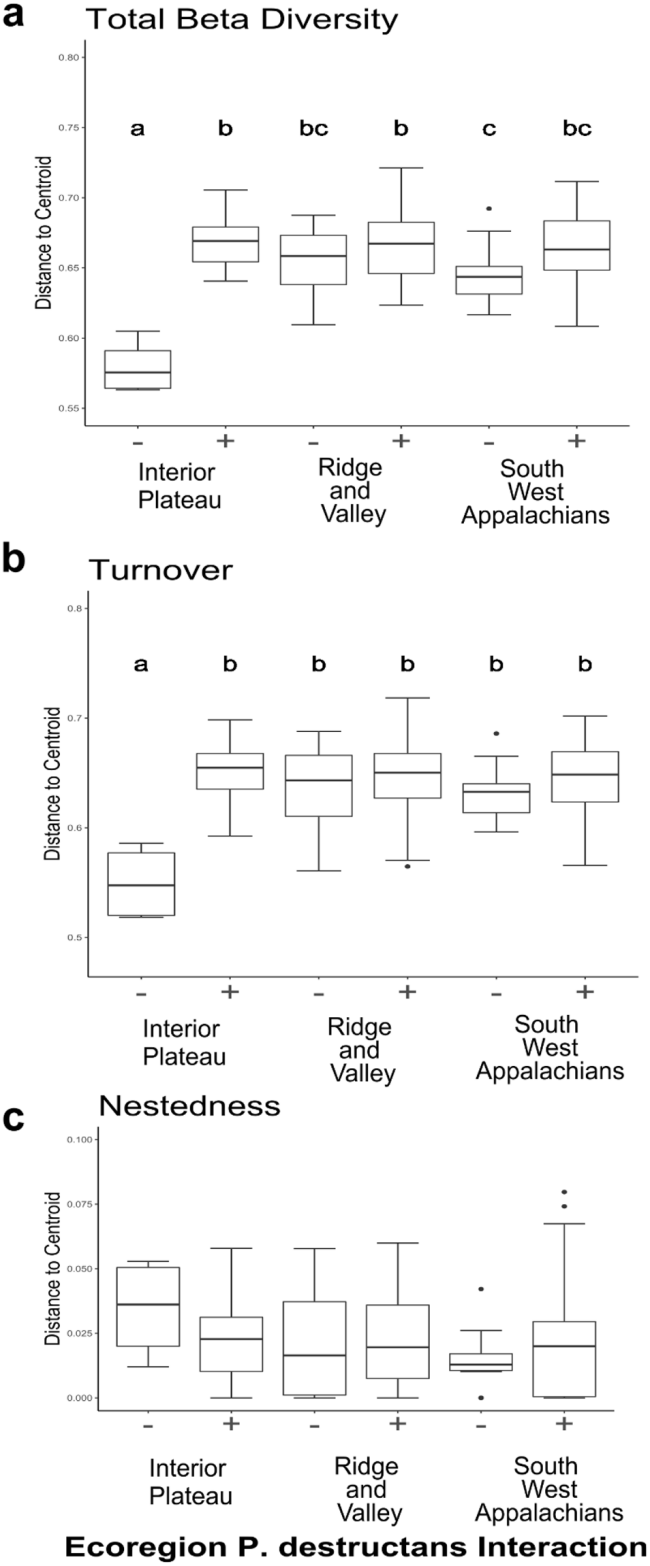
Betadisper analysis comparing beta diversity measured as multivariate dispersion across the interaction of ecoregion and *P. destructans* status, for (**a**) total beta diversity (SOR), (**b**) turnover (SIM), and (**c**) nestedness (SNE). Different lowercase letters indicate a significant difference (*p* ≤ 0.05) between groups, lowercase letters are missing from panel (**c**) due to lack of significant differences between groups. There is a significant interaction between *P. destructans* status and ecoregion for both total beta diversity as well as turnover (SOR: F_5, 243_ = 15.232, *p* ≤ 0.05; SIM: F_5,243_ = 8.646, *p* ≤ 0.05). Graphs were produced using the *ggplot2* package (version 3.3.2) in R^25^.

**Figure 6:**
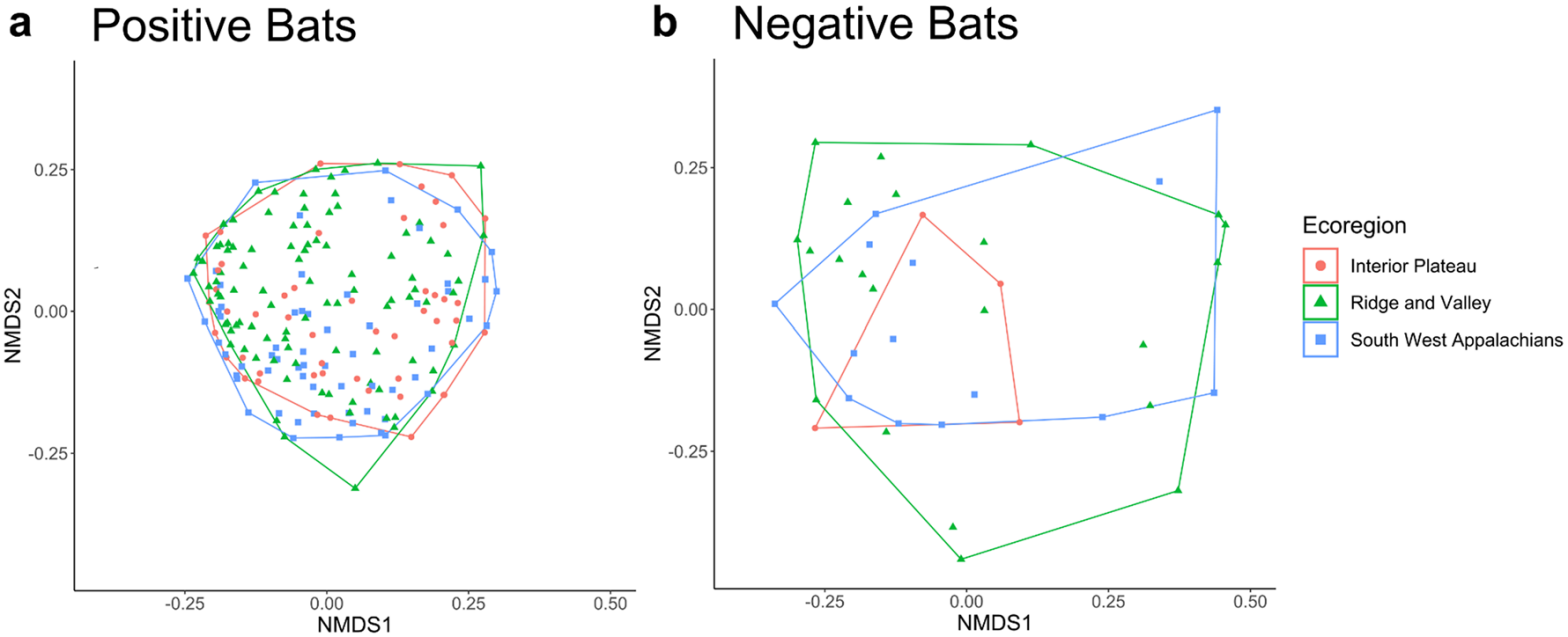
Non-metric multidimensional scaling ordination for. (**a**) *P. destructans* positive and (**b**) *P. destructans* negative bats across ecoregions (**a**) stress 0.17; (**b**) stress 0.14. There is a significant effect of *P. destructans*, year and ecoregion (*p* < 0.05), however, there is no significant interaction between *P. destructans* status and ecoregion. There is significant variation in dispersion across ecoregions for *P. destructans* negative bats (**b**). Graphs were produced using the *ggplot2* package (version 3.3.2) in R^25^.

All three measures of beta diversity lacked a distance-decay relationship (GLM; SOR: z = 0.79, *p* > 0.05; SIM: z = 0.86, *p* > 0.05; SNE: z = 0.96, *p* > 0.05; Fig. 2, Supplemental File 1E). There was no difference in the rate of decay between positive and negative bats for total beta diversity (ANOVA; SOR: *p* > 0.05, Fig. 2b), the turnover component of beta diversity (ANOVA; SIM:  *p* > 0.05; Fig. 2c), or nestedness (ANOVA; SNE: *p* > 0.05 Fig. 2d). Multivariate dispersion was statistically different between *P. destructans* positive and negative bats for total beta diversity (betadisper; SOR: F_1, 247_ = 5.89, *p* ≤ 0.05, Supplemental File 1G). Interestingly, analyses of multivariate dispersion indicated that there was a significant interactive effect between *P. destructans* status and ecoregion for both total beta diversity and turnover (betadisper; SOR: F_5,243_ = 15.232, *p* ≤ 0.05; SIM: F_5,243_ = 8.646, *p* ≤ 0.05, Supplemental File 1G; Figs. 5a,b, 6). Post-hoc analysis of the interaction term for total beta diversity showed that dispersion was not different across ecoregions for *P. destructans* positive bats but varied for *P. destructans* negative bats (Fig. 5a). In general, dispersion was large in *P. destructans* positive bats, with negative bats within the Interior Plateau having significantly less dispersion (Fig. 5a), largely driven by a difference in the turnover component (Fig. 5b). PERMANOVA revealed that average community composition (multivariate centroids) differed between *P. destructans* status and ecoregion when analyzing both total dissimilarities and the turnover component, but not nestedness, and that these effects were independent of each other (Fig. 6, Table 2).

**Table 2:** PERMANOVA results for total beta diversity (SOR), the turnover component of beta diversity (SIM), and the nested component of beta diversity (SNE).

	Df	Sums of squares	Mean squares	F test	R^2^	*p* value
**Total beta diversity**						
*P. destructans* status	1	0.746	0.746	1.7403	0.006	0.018*
Ecoregion	2	1.427	0.713	1.664	0.012	0.011*
year	1	0.795	0.794	1.8541	0.007	0.001***
site	45	24.357	0.541	1.2626	0.216	0.771
*P. destructans* status:Ecoregion	2	0.905	0.452	1.055	0.008	0.107
Residuals	197	84.454	0.428		0.749	
Total	248	112.683			1	
**Turnover**						
*P. destructans* status	1	0.862	0.861	2.139	0.008	0.003**
Ecoregion	2	1.591	0.795	1.975	0.014	0.033*
year	1	0.605	0.605	1.502	0.005	0.012*
site	45	23.712	0.526	1.308	0.221	0.72
*P. destructans* status:Ecoregion	2	0.876	0.437	1.087	0.008	0.22
Residuals	197	79.344	0.402		0.741	
Total	248	106.99			1	
**Nestedness**						
*P. destructans* status	1	− 0.007	− 0.007	− 7.92	− 0.045	1
Ecoregion	2	− 0.01	− 0.005	− 5.955	− 0.067	0.347
year	1	0.009	0.009	11.111	0.063	0.105
site	45	− 0.01	− 0.0002	− 0.269	− 0.069	0.737
*P. destructans* status:Ecoregion	2	− 0.0003	− 0.0001	− 0.22	− 0.002	0.745
Residuals	197	0.174	0.0008		1.121	
Total	248	0.156			1	

There is a significant difference between *P. destructans* status, Ecoregion, and year for both SOR and SIM (*p* < 0.05).

## Discussion

This study characterized the metacommunity structure of host microbial assemblages in the presence of a fungal pathogen. Overall, support was found for both of our hypotheses, we determined that the presence of *P. destructans* correlated with a change in cutaneous microbial metacommunity structure and loss of indicator OTUs from the core skin assemblage. Additionally, we found that the presence of *P. destructans* correlated with a change in relationship between community structure and an environmental structuring variable. These results suggest that the presence of *P. destructans* alters cutaneous microbial metacommunity structure by intensifying local processes, such as species sorting mechanisms or antagonistic species interactions.

The cutaneous microbial assemblages of *P. destructans* negative bats were characterized by a nested metacommunity structure with clumped species loss. The presence of numerous indicator taxa within negative bats further supported the inference of a nested metacommunity structure. Nested metacommunities have been observed in a variety of organisms, including Bryophytes^14^, macroinfauna^27^, and bats^13^, and likely represent variation in species-specific characteristics such as dispersal ability and tolerance to environmental conditions^5^. This is supported by previous work, which has shown the importance of host environment in shaping the cutaneous microbial assemblage^12^ and suggests that OTU-specific tolerances to host environmental conditions might drive the clumped OTU loss seen in *P. destructans* negative bats.

The core microbiome is defined as the taxonomic identity of the most common bacterial taxa within a system^28^. Pairing of the EMS and indicator analysis results for *P. destructans* positive bats suggested a loss of bacterial OTUs from the core microbiome. This loss might suggest alteration in community function and host defense against pathogens^24,29,30^. Nucleotide BLAST searches (based on ~ 250 bp region) revealed that indicator taxa identified in this study (Supplemental Table) were not genetically identical to cultured bacteria with in vitro anti-*P. destructans* activity identified in Grisnik et al.^24^. However, seven of 363 indicator taxa were identified to the same genera of anti-*P. destructans* bacteria identified previously^24^, including *Nocardia, Rhodococcus, Streptomyces, Luteibacter, Lysobacter,* and *Sphingomonas*. Each of these bacterial genera were detected on both positive and negative bats. Alternatively, bacteria with anti-fungal activity could have been gained to form the core microbiome of *P. destructans* negative bats, but additional work is required to mechanistically explain the correlational patterns found here. It is also important to acknowledge that approaches to understand assemblage function in vitro likely oversimplify complex inter- and intra-specific interactions at the community level, and further work to understand how bacterial function relates to fungal pathogenicity is warranted. The two most common phyla of indicator taxa were Proteobacteria (*P. destructans* positive n = 2 of 14, *P. destructans* negative n = 104 of 363) and Actinobacteria (*P. destructans* positive n = 12 of 14, *P. destructans* negative n = 95 of 363). Interestingly, the majority of indicator taxa for *P. destructans* positive bats are from the family Micrococcaceae (n = 11 of 14) which contains *Micrococcus,* a genus of well documented skin colonizers^19^. The more common indicator taxa of *P. destructans* negative bats include the phyla Planctomycetes (n = 35 of 363) and Acidobacteria (n = 28 of 363), both of which are commonly found in soils^31,32^. This might suggest increased interactions between cutaneous microbial assemblage and environmental microbes in the absence of *P. destructans*, however, manipulative studies would be required to address this question.

The cutaneous microbial assemblage of *P. destructans* positive bats exhibited turnover with boundaries clumped along an environmental gradient (Clementsian structure). Clementsian structure is known to be common in both free living^14^ and host-associated microbial assemblages^15^. Clementsian metacommunities can arise from antagonistic interactions preventing the coexistence of some taxa^6,27^. Interestingly, previous work showed an inverse relationship between *P. destructans* positive bats and bacteria that inhibited growth of *P. destructans*^24^, suggesting that antagonistic interactions might drive the shift to Clementsian metacommunity structure in microbial assemblages of positive bats.

The lack of a distance-decay relationship in microbial assemblages suggests either a lack of dispersal limitation or absence of species sorting mechanisms driving the assembly of bat cutaneous microbial assemblages. Since bat host environment (ecoregion) had a significant impact on average assemblage structure and there was no significant distance-decay relationship, we can conclude that dispersal limitation does not have a predominant role in the assembly of the cutaneous microbial assemblages of *P. subflavus*. Bacterial dispersal limitation is consistent with previous work that has shown a lack of population structure in Appalachian bat species^33,34^. This suggests that frequent roost switching and host dispersal may provide opportunities for microbial dispersal, and therefore, homogenization of bacterial assemblages across the region. Barriers to microbial dispersal between individual bats might be low, suggesting that the level of selection for microbial assemblage formation might be occurring at the colony level rather than the individual level^35^. Other studies have attributed environmental heterogeneity as the underlying driver of distance-decay relationships in microbial assemblages^36–39^. The lack of a distance-decay pattern driven by environmental heterogeneity could be due to the similarity of cave environments across our study system, as bats were sampled during the winter hibernation period, and not on a variety of summer/winter roost sites. Alternatively, variation within cave environments across the study system could result in a patchy distribution, rather than a geographically constrained gradient of environmental heterogeneity. The overall influence of host environment and species sorting mechanisms have been observed in the literature, as other studies have shown an influence of site on cutaneous microbial assemblage structure for a variety of host taxa^12,40,41^. Our results suggest the role of the host environment in shaping microbial communities through species sorting regardless of *P. destructans* status. The presence of *P. destructans* does not alter the rate (slope) of distance-decay in microbial assemblages across geographic space. In the context of community assembly, we found a lack of a *P. destructans* mediated change in dispersal limitation and/or species sorting in bacterial assemblage formation. Previous work has suggested an inverse pattern showing that as levels of disturbance increase, the rate of turnover within assemblages decreases, suggesting that disturbances can act as ecological filters^42^.

Results of permutational models indicated the role of the environment in shaping the bat cutaneous microbial assemblage. The PERMANOVA analysis indicates that the presence of *P. destructans* correlates with a difference in average community composition. Additionally, analysis of multivariate dispersion indicates that there is a significant interaction between *P. destructans* status and the environment, which suggests that the presence of *P. destructans* can alter the relationship between community structure and structuring variables, specifically the ecoregion where a bat is located. Of particular interest is the lack of significant differences in dispersion across ecoregions for *P. destructans* positive bats, despite significant differences for negative bats. In general, *P. destructans* positive bats have higher dispersion than negative bats. The presence of *P. destructans* within the Interior Plateau correlates with increased dispersion in the turnover component of beta diversity compared to negative bats within that ecoregion. When the analysis of multivariate dispersion is coupled with the lack of a distance-decay relationship, it suggests that local processes (such as antagonistic interactions or species sorting) may be stronger in the presence of *P. destructans*. Previous work has suggested that disturbance increases the importance of species sorting mechanisms through the filtering of species that cannot persist within the disturbed environment^43^.

We assessed metacommunity structure in cutaneous assemblages as they responded to the progression of fungal disease. There was no significant relationship between community similarity and fungal load, which serves as a proxy for disease progression. This suggests that the presence of *P. destructans* alone might be enough to alter the average microbial assemblage. Previous work has shown the opposite pattern with increasing fungal load being positively correlated with assemblage dissimilarity^44^. However, this study was done on salamanders infected with a chytrid fungus in a mesocosm setting, which could explain the conflicting results. Alternatively, due to the hierarchical structure of our data (bats nested within caves), the patterns we observed could be a result of site level averages rather than being representative of bacterial-fungal interactions on individual bats. While this is a valid concern, it has been shown that colony-level dynamics rather than individual identity better explain bat cutaneous microbial assemblage structure^35^, suggesting valid ecological patterns observed during this study. The presence of *P. destructans* may drive the formation of a unique assemblage through deterministic processes, but within a disease state category (*P. destructans* positive or negative) the variation might be best explained by stochastic or species-specific factors. While our study failed to find a relationship between assemblage-level similarity and fungal load, previous work has shown a correlation between pathogenic fungal load and specific bacterial taxa on bats^24^. *Pseudogymnoascus destructans* load may induce OTU-specific abundance responses but not influence overall assemblage similarity in terms of species presence. Future research at a fine-scale temporal resolution and quantifying microbial relative abundances is necessary to understand the effects of disease progression on microbial assemblage structure.

In order to understand interactions between host-associated microbial assemblages and pathogens it is important to take scale into account. The goal of this study was to elucidate if the presence of a fungal pathogen correlates with changes in metacommunity structure and the variables that structure these communities as they relate to assembly mechanisms. Results suggest that invasion of these communities by a fungal pathogen correlates with a shift in metacommunity structure likely driven by intrinsic factors that alter community assembly mechanisms. We hypothesize that the change in community structure is caused by increased strength of local processes within assemblages. Understanding how the host cutaneous microbial assemblage interacts with a fungal pathogen, specifically within a metacommunity context, is important for elucidating how this assemblage potentially protects hosts from pathogens. Future work should aim to better understand potential antagonistic interactions within microbial assemblages as they may help describe observed shifts in metacommunity structure and role in pathogen defense. Increased understanding of these antagonistic interactions has potential conservation implications, with recent interest in augmenting host cutaneous microbial assemblages with antifungal bacterial taxa, as a way to protect declining host populations^24,30^. Additionally, future experimental work in a controlled setting is required to confirm the community assembly processes structuring bat skin assemblages. Lastly, this study was conducted exclusively on bacterial communities, future work should incorporate other members of the host associated microbiome including viruses and fungi.

## Methods

### Sample collection

Swabs from 369 individuals of adult *P. subflavus* were collected during statewide surveys between December 2016 and March 2019 across 57 cave sites in Tennessee. After bioinformatics processing and quality control, 249 *P. subflavus* from 48 sites were statistically analyzed (see methods below and supplemental file 1D for metadata). Each bat had its cutaneous microbial assemblage sampled following the protocol outlined in Grisnik et al.^24^. Briefly we took five swab (sterile Puritan polyester tipped swabs, Puritan, Guilford Maine) strokes of each bat muzzle/ear and five from wings/fur while avoiding the mouth using one sterile swab per bat individual. Due to the conservation status of *P. subflavus*, when possible, bats were left hanging attached to their roost and swabbed without disturbing torpor. All samples were stored on ice in the field and permanently at − 20 °C until processing. This study was approved by the Tennessee Technological University Institutional Animal Care and Use Committee (TTU-16-17-003) and USFWS (2009-038). All methods were carried out following relevant guidelines and regulations. We isolated DNA from 369 bats using the Qiagen DNeasy PowerSoil HTP 96 kit following the manufacturer’s protocol. Each plate of 96 samples contained a single DNA extraction blank (n = 8 total blanks) to filter out kit-based contamination during bioinformatics processing and quantitative PCR reactions (see below). When setting up each DNA extraction plate and subsequent library preparation, the location of samples on each 96 well plate was randomized, in order to reduce biased effects of well-to-well contamination^45^. Extracted DNA was then used for molecular characterization of the microbial community, as well as, qPCR for the detection of *P. destructans*.

### Characterization of microbial community

Each step of library preparation (DNA isolation, PCR setup, and post PCR processes) was separated into specific PCR cabinet hoods with designated pipettes to minimize environmental and/or cross-contamination. Pipettes were autoclaved, and UV crosslinked periodically throughout library preparation. Once isolated, DNA was concentrated, using an Eppendorf Vacufuge plus, to a final volume of ~ 25 µL. After concentration, PCR amplification and high-throughput sequencing was performed following a modified version of the Illumina 16S Metagenomic Sequencing Library Preparation protocol. Specifically, we targeted the V4 region of 16S rRNA marker using primers 806R/515F (ref.^46^). Each PCR reaction contained 12.5 µL MCLAB I-5 Hi-Fi taq mastermix, 1 µL of 806R (10 µM), 1 µL of 515F (10 µM), 5.5 µL PCR grade water, and 5 µL DNA template. PCR amplification was performed with an initial denaturation at 95 °C for 2 min, followed by 35 cycles of 98 °C for 10 s, 55 °C for 15 s, and 72 °C for 5 s, with a final extension cycle of 72 °C for 5 min. MAGBIO High-prep magnetic beads were used to remove primer/adapter dimers after amplicon PCR and indexing steps. Samples were quantified with a Promega Quantus Fluorometer then normalized, pooled at a 4 picomolar concentration, and loaded onto an Illumina MiSeq v2 flow cell. Sequencing was performed in eight separate runs each using a 500-cycle reagent kit (paired-end, 2 × 250 bp reads).

### Quantitative PCR

To determine the presence or absence of *P. destructans* within a sample we followed the protocol outlined by Muller et al.^47^ to amplify the fungal intergenic spacer region (IGS). Each reaction was run in triplicate on an Agilent AriaMx Real-Time PCR system, and contained 5 µL 2 × Primetime MasterMix, 0.4 µL forward primer (20 µM), 0.4 µL reverse primer (20 µM), 0.1 µL probe (20 µM), 3.1 µL PCR grade water, and 1 µL sample DNA for a total of 10 µL per reaction. Thermocycling conditions included a 3-min activation step at 95 °C, then 50 cycles of 95 °C for 3 s and 60 °C for 30 s. Each plate included both a known concentration of synthetically made *P. destructans* DNA (gBlocks; Integrated DNA Technologies) to serve as a positive control and a no template negative control (run in triplicate) to account for within plate contamination. A positive sample was indicated by exponential amplification in triplicate with a C_t_ value of less than 40^47,48^. If samples did not test positive in triplicate, they were re-tested, and were considered positive if there was amplification in at least one of the three subsequent reactions^49^. In order to quantify *P. destructans* fungal load, qPCR reactions of a serial dilution of synthetic DNA was used to generate the standard curve equation y = − 0.2936x + 11.439, with x being the average C_t_ value for each sample run in triplicate, and y being the log DNA copy number.

### Bioinformatic analysis

Amplicon sequencing reads were processed using mothur v1.42.1 (ref.^50^). A total of 48,442,995 raw data sequence reads were obtained from eight sequencing runs. Paired-end reads were assembled into contigs, and sequences containing homopolymers greater than 8 nucleotides or any ambiguous base calls were removed. We identified unique sequences and aligned them to the SILVA v123 bacterial reference database^51^. After alignment, sequences were trimmed to the V4 region and *pre*-*clustered* allowing for two-nucleotide differences between clusters. Chimera removal was then done using the *vsearch* function in mothur^52^. Sequences were classified into taxonomic lineages and reads identified as Archaea, Eukaryota, chloroplast, mitochondria, and unknown were removed. The *cluster.split* command in mothur was used to cluster sequences into operational taxonomic units at 97% similarity^53^. OTUs that appeared less than five times were considered rare and were removed from the dataset. Additionally, OTUs that were found within the DNA extraction blanks were also removed (n = 1669 OTUs). OTUs were selected as the focal taxonomic level rather than ASVs (amplicon sequence variants), as previous work has shown that there is negligible difference in ecological patterns observed when OTU or ASV data are analyzed^54^. In total 5,701,307 sequences (11.7%) passed all quality control steps. We compared final library sizes across all samples and found that they were significantly different (Kruskal–Wallis: χ^2^[^2^] = 83.98, *p* < 0.05). Therefore, the data were rarefied by subsampling each library at 1200 sequence reads^55^. Data were subsampled as previous work has shown that it is an effective way to account for variation in library size^55^. The final OTU × sample matrix included 268 samples of *P. subflavus*. Since we were interested in observing variation between *P. destructans* positive and negative bats over geographic distance we standardized the data so that geographic distances between sample sites were equal for *P. destructans* positive and negative *P. subflavus*. This resulted in a total of 249 *P. subflavus* (40 *P. destructans* negative and 209 positive) used for the statistical analysis described below*.* All mothur commands are included in the Supplemental File 1A for reproducibility purposes.

### Statistical analyses

Previous work has shown that rare OTUs can skew the results of elements of metacommunity structure (EMS) analysis^5^. Prior to conducting analyses, all OTUs that summed to less than two were removed resulting in 12,603 OTUs in the complete OTU × sample matrix. All analyses were conducted in R 3.4.2 (ref.^56^) using α = 0.05 unless multiple comparisons were made, and thus Bonferroni adjusted.

We used the *metacom* package (version 1.5.3) in R^26^ to determine if the presence of *P. destructans* correlated with changes in metacommunity structure of cutaneous microbial assemblages as outlined in Leibold and Mikkelson^6^ and Presley et al.^5^ following the Elements of Metacommunity Structure (EMS) framework. We evaluated three EMS metrics (coherence, turnover, and boundary clumping) using a site-by-species presence/absence matrix to determine metacommunity structure. *Coherence* was assessed as the number of embedded species absences, or the number of gaps/interruptions in species distributions, within an ordinated community matrix (Fig. 1). The number of observed absences was compared to an expected number of absences determined through the formulation of a null distribution created from simulated matrices with 1000 iterations. Negative coherence describes a pattern of significantly more observed embedded absences than predicted by the null model, and a metacommunity perceived with a “checkerboard” appearance (Fig. 1a). A random metacommunity is identified when there is a non-significant difference between observed and expected embedded absences. Significantly less embedded absences indicate positive coherence, which is suggestive of species responding to a structuring gradient. The latter pattern requires further analysis of *turnover* and *boundary clumping* for more specific designation of metacommunity structure. *Turnover*, was assessed to describe the number of times a species is replaced by another between two sites. As with coherence, the number of turnover events observed is compared to the number of expected events using a null model prediction. If there are significantly more replacements than expected by chance, this represents positive turnover and signals a Clementsian (Fig. 1e) or evenly spaced (Fig. 1d) metacommunity structure. If there are significantly less replacements than expected, this represents negative turnover and signals a nested metacommunity structure. *Boundary clumping* was evaluated using Morisita’s index to describe how distinct blocks of species are clumped along a range boundary. A Morisita’s index significantly greater than one, indicates clumped species loss (positive turnover: Fig. 1e; negative turnover: Fig. 1c), whereas, an index value significantly fewer than one indicates evenly spaced, i.e. hyperdispersed, species loss (positive turnover: Fig. 1d; negative turnover: Fig. 1b). To assess significance for each EMS metric we used the default fixed-proportional null model (“r1”), 1000 permutations, and allowed for null matrices to have empty rows and columns^26^. EMS analysis was performed using the *metacommunity* function^26^ and analyses for *P. destructans* positive and negative samples were run separately. Metacommunity patterns were visualized using the *Imagine* function within the *metacom* package.

In order to further describe how *P. destructans* status correlated with changes in community structure we performed an indicator analysis using the *multipatt* function in R package *indicspecies* (version 1.7.9;^57^) on the presence-absence transformed OTU data table. We used a generalized linear mixed-effects model (GLMM) with the *glmer* function (package *lme4*;^58^) assuming a Poisson error structure, with site set as the random effect to account for nested data, to compare the rarefied abundance of significant indicator taxa of *P. destructans* negative bats across all samples. Additionally, we compared the amount of *P. destructans* present (number of copies using qPCR) to the rarefied abundance of indicator taxa using a GLMM assuming a Poisson error structure, with site set as the random effect. In order to explore the compositional nature of our dataset^59^, we repeated the above analyses following the pipeline outlined by Gloor et al.^59^ using a clr transformed dataset. Complete methods and code for this analysis are included in Supplemental File 1F.

To determine if fungal load influenced community dissimilarity, we converted fungal copy number to a distance matrix representing differences between samples using the *dist* function with Euclidean distances. This allowed for us to determine if bats having more similar fungal loads have more similar microbial assemblages. The resulting distance matrix was compared to total beta diversity (Sørensen dissimilarity: SOR), the turnover (Simpson dissimilarity: SIM), and nested (nestedness: SNE) components of total beta diversity (package *betapart;*^60^). To address the nested structure of the data, a dummy variable was created to describe the pairwise site level comparisons by grouping the samples by geographic distances into a categorical “site contrast” variable. Due to issues resulting in singular fit of mixed models, we then averaged both fungal load and beta diversity (SOR, SIM, and SNE) by the “site contrast” variable resulting in an average dissimilarity between two samples, thus removing the nested structure of the data (Supplemental File 1B). We then used a GLM (function *glm)* to compare average fungal load difference to average beta diversity metrics. The GLM was run assuming a binomial distribution with log transformed fungal load dissimilarity set as a fixed effect.

To assess how the presence of *P. destructans* correlated with differences in the rate of turnover and patterns of distance-decay, we compared total beta diversity (SOR), the turnover (SIM), and nestedness (SNE) components of Sørensen diversity across geographic distances. Pairwise geographic distances between samples were computed as the Euclidian distance between sample points using the *dist* function in the package *vegan*^61^. Similar to the analysis comparing assemblage dissimilarity to fungal load difference, beta diversity (SOR, SIM, and SNE) was averaged by site contrast, in order to remove the nested structure of the dataset, and to accommodate issues of singular fit in the mixed models. The relationship between average community dissimilarity and average geographic distance (distance-decay relationship) was determined using a generalized linear model (GLM). GLM was performed assuming a binomial distribution using the *glm* function with geographic distance, *P. destructans* status, and the interaction between these variables being set as fixed effects and a Bonferroni adjusted p-value of 0.016. The analysis was conducted using type II sum of squares with the *Anova* function in the package *car*^62^ to account for unequal sample sizes across groups.

To elucidate how environmental variables influenced beta diversity across *P. destructans* status we compared variation in beta diversity, measured as multivariate dispersion (function *betadisper*, package *vegan*), across *P. destructans* status, ecoregion (specifically ecoregion level 3, as delineated by the Environmental Protection Agency), and *P. destructans* × ecoregion interaction. Ecoregion was selected as the environmental variable as it represents a composite variable encompassing multiple fine scale environmental factors. A Tukey’s post hoc test was then used to determine pairwise differences between the groups of the interactive effect. We used permutational multivariate analysis of variance (PERMANOVA) stratified by site with 999 permutations using the *adonis* function (package *vegan*) on SOR, SIM, and SNE dissimilarity metrics to assess the influence of ecoregion on average assemblage similarity. Explanatory variables included ecoregion, *P. destructans* status, the *P. destructans* × ecoregion interaction, as well as year and site as covariates accounting for data structure. The PERMANOVA assumption of homogeneity of variance was violated, however previous work^63^ has shown that PERMANOVA is robust to violations of this assumption when the variable with the greater sample size has a larger variance, as seen with our data. All R code is included in the Supplemental File 1C for reproducibility purposes.

## Supplementary Information

Supplementary Information 1.

Supplementary Information 2.

## Data Availability

All sequence data were submitted to GenBank SRA under the accession number PRJNA691025. All mothur code and all R code has been made publicly accessible in the supplemental file.
