# Supplementary material for: The presence of Pseudogymnoascus destructans, a fungal pathogen of bats, correlates with changes in microbial metacommunity structure
Source: Sci Rep. 2021 Jun 3;11:11685. doi: 10.1038/s41598-021-91118-1 (PMC8175404; doi:10.1038/s41598-021-91118-1)
Supplement: Supplementary file 1 — Supplementary Information 1. [file 41598_2021_91118_MOESM1_ESM.pdf]

Supplemental File

Title: The presence of Pseudogymnoascus destructans, a fungal pathogen of bats, correlates with changes in microbial metacommunity structure

Matthew Grisnik[1], Joshua B Grinath[2], Donald M Walker[1*]
[1]Middle Tennessee State University, Department of Biology, Murfreesboro, Tennessee 37132, USA
[2]Idaho State University, Department of Biological Sciences, Pocatello, Idaho 83209, USA
*Corresponding author
Donald M. Walker, PhD
Donald.Walker@mtsu.edu
615-904-8382

Table of Contents

Supplemental File 1A mothur code	2
Supplemental File 1B Sampling Scheme	4
Supplemental File 1C R code	5
Supplemental File 1D Metadata	66
Supplemental File 1E Modeling Results	72
Supplemental File 1F Compositional Analysis	80
Supplemental File 1G Betadisper Results	88

Supplemental File 1A mothur code

```
Linux version
Using ReadLine
mothur v.1.42.1
Last updated: 01/09/2020
by
Patrick D. Schloss
Department of Microbiology & Immunology
University of Michigan
http://www.mothur.org
When using, please cite:
Schloss, P.D., et al., Introducing mothur: Open-source, platform-independent, community-supported software for describing and comparing microbial communities. Appl Environ Microbiol, 2009. 75(23):7537-41.
Distributed under the GNU General Public License
Type 'help()' for information on the commands that are available
For questions and analysis support, please visit our forum at https://forum.mothur.org
Type 'quit()' to exit program
[NOTE]: Setting random seed to 19760620.
Interactive Mode
 mothur > make.contigs(file=200109_stability.txt, processors=30)
 mothur > pcr.seqs(fasta=current, group=current, oligos=oligos.txt, pdiffs=1, rdoiffs=3)
 mothur > pcr.seqs(fasta=current, group=current, oligos=oligos.txt, pdiffs=1, rdiffs=3)
mothur > summary.seqs(fasta=current)
 mothur > screen.seqs(fasta=current, group=current, maxambig=0, maxlength=256, maxhomop=8, minlength=248)
 mothur > summary.seqs(fasta=current)
mothur > unique.seqs(fasta=current)
 mothur > summary.seqs(fasta=current)
 mothur > count.seqs(name=current, group=current)
 mothur > summary.seqs(count=current)
 mothur > pcr.seqs(fasta=silva.bacteria.fasta, start=11894, end=25319, keepdots=F)
 mothur > system(mv silva.bacteria.pcr.fasta silva.v4.fast)
 mothur > system(mv silva.bacteria.pcr.fasta silva.v4.fasta)
 mothur > system(mv silva.v4.fast  silva.v4.fasta)
mothur > align.seqs(fasta=200109_stability.trim.contigs.pcr.good.unique.fasta, reference=silva.v4.fasta)
 mothur > summary.seqs(fasta=current, count=current)
 mothur > screen.seqs(fasta=current, count=current, start=1968, end=11550)
mothur > summary.seqs(fasta=current, count=current)
 mothur > filter.seqs(fasta=current, vertical=T, trump=.)
 mothur > summary.seqs(fasta=current, count=current)
 mothur > unique.seqs(fasta=current, count=current)
mothur > set.current(count=200109_stability.trim.contigs.pcr.good.good.count_table, fasta=200109_stability.trim.contigs.pcr.good.unique.good.filter.fasta, processors=24)
 mothur > unique.seqs(fasta=current, count=current)
 mothur > summary.seqs(fasta=current, count=current)
```

```
mothur > pre.cluster(fasta=current, count=current, diffs=2)
mothur > summary.seqs(fasta=current, count=current)
mothur > chimera.uchime(fast=current, count=current, dereplicate=t)
mothur > chimera.uchime(fasta=current, count=current, dereplicate=t)
mothur > summary.seqs(count=current)
mothur > remove.seqs(fasta=current, accnos=current)
mothur > summary.seqs(fasta=current, count=current)
mothur > classify.seqs(fasta=current, count=current, reference=silva.nr_v132.pcr.align,
taxonomy=silva.nr_v132.tax, cutoff=80)
mothur > summary.seqs(fasta=current, count=current)
mothur > remove.lineage(fasta=current, count=current, taxonomy=current, taxon=Chloroplast-
Mitochondria-unknown-Archaea-Eukaryota)
mothur > summary.tax(taxonomy=current, count=current)
mothur > cluster.split(fasta=current, count=current, taxonomy=current, splitmethod=classify,
taxlevel=4, cutoff=0.03)
 mothur > summary.seqs(fasta=current, count=current)
mothur > make.shared(list=current, count=current, label=0.03)
 mothur > get.groups(shared=current, groups=Negativelib1-Negativeplate9-Negativeplate8-
Negativeplate6-Negativeplate5-Negativeplate4-Negativelib2-Negativelib3)
mothur > remove.rare(shared=current, nseqs=5)
mothur > classify.otu(list=current, count=current, taxonomy=current, label=0.03)
mothur > count.groups(shared=current)
mothur > sub.sample(shared=current, size=1200)
```

Supplemental File 1B Sampling Scheme

Analysis	Sample type
Metacommunity Diagnosis	Individual Samples
Indicator Analysis	Individual Samples
Fungal Load	Site Average
Distance-Decay	Site Average
Environmental Variables	Individual Samples

Supplemental File 1C R code

```
library(vegan)
library(metacom)
library(betapart)
library(rcompanion)
library(geosphere)
library(nortest)
library(ggplot2)
library(reshape)
library(car)
library(tidyverse)
library(dplyr)
library(nlme)
library(lme4)
library(Hmisc)
library(indicspecies)

stability<-read.table(file= "stability", header=TRUE)
metadata<-read.csv(file="PESU_site_5.csv")
data<-merge(metadata,stability, by="Group")
rownames(data)<-data$Group
set.seed(1138)
##########################################
#EMS analysis
##########################################
####DataPrep####
##Positive
###
positive<-subset(data, pd=="1")
posmeta<-positive[1:23]
pos_otus<-positive[24:23869]
#Remove zeros
colSums(pos_otus)
i<-(colSums(pos_otus, na.rm=T)!=0)
posotusnonzero<-pos_otus[,i]
posotuzeros<-pos_otus[,!i]
colSums(posotuzeros)
posoturemovezero<-posotusnonzero
#Remove 1s
colSums(posoturemovezero)
i<-(colSums(posoturemovezero, na.rm=T)!=1)
posotusnonzero<-posoturemovezero[,i]
posotuzeros<-posoturemovezero[,!i]
colSums(posotuzeros)
posoturemovezero<-posotusnonzero
```

```
colSums(posoturemovezero)
PA_pos<-decostand(x=posoturemovezero, method="pa")
####Metacom####
metacom_pos_1<-(Metacommunity(PA_pos, scores = 1, method = "r1",
                    turnoverMethod = "EMS", sims = 99, order = TRUE,
                    allowEmpty = TRUE, binary = TRUE, verbose = TRUE, seed = 1))

Imagine(comm=PA_pos, col=c(0,1), order=TRUE, scores=1, fill=TRUE, xlab="", ylab="", yline=2, xline=2,
sitenames=rownames(PA_pos), speciesnames=colnames(posoturemovezero),  binary=TRUE)
####end####
###
##Negative
###
negative<-subset(data, pd=="0")
neg_otus<-negative[24:23869]
negmeta<-negative[1:24]
colSums(neg_otus)
i<-(colSums(neg_otus, na.rm=T)!=0)
negotusnonzero<-neg_otus[,i]
negotuzeros<-neg_otus[,!i]
colSums(negotuzeros)
negoturemovezero<-negotusnonzero
#Remove 1s
colSums(negoturemovezero)
i<-(colSums(negoturemovezero, na.rm=T)!=1)
negotusnonzero<-negoturemovezero[,i]
negotuzeros<-negoturemovezero[,!i]
colSums(negotuzeros)
negoturemovezero<-negotusnonzero


PA_neg<-decostand(x=negoturemovezero, method="pa")
####Metacom####
metacom_neg<-(Metacommunity(PA_neg, scores = 1, method = "r1",
                   turnoverMethod = "EMS", sims = 1000, order = TRUE,
                   allowEmpty = TRUE, binary = TRUE, verbose = TRUE, seed = 1))

Imagine(comm=PA_neg, col=c(0,1), order=TRUE, scores=1, fill=TRUE, xlab="", ylab="", yline=2, xline=2,
sitenames=rownames(PA_neg), speciesnames=colnames(negoturemovezero),  binary=TRUE)
####end####
####end####
###########################
#Indicator analysis
###########################
#PESU + vs PESU -
####Indicator####
indicator1<-subset(data, sp=="PESU")
meta<-indicator1[1:23]
```

```
indicator1_otus<-indicator1[24:23871]
colSums(indicator1_otus)
i<-(colSums(indicator1_otus, na.rm=T)!=0)
indicator1otusnonzero<-indicator1_otus[,i]
indicator1otuzeros<-indicator1_otus[,!i]
colSums(indicator1otuzeros)
indicator1oturemovezero<-indicator1otusnonzero
indicator1test<-multipatt(indicator1oturemovezero, indicator1$pd, func="IndVal.g",
control=how(nperm=999))
options(max.print = 10000)
summary(indicator1test)

indicator1<-select(data, "Group", "site", "copies",
"pd","text","Otu000193","Otu000201","Otu000728","Otu000342","Otu000298","Otu000290","Otu0002
51","Otu000310","Otu000266","Otu000455","Otu001016","Otu001058","Otu000362","Otu001460","Ot
u000604","Otu000374","Otu000453","Otu002066","Otu001333","Otu002979","Otu002334","Otu00147
3","Otu001616","Otu001376","Otu000836","Otu000730","Otu000524","Otu002926","Otu001091","Otu
002137","Otu001568","Otu001338","Otu001102","Otu001965","Otu002231","Otu001449","Otu001551
","Otu001304","Otu001894","Otu002378","Otu000722","Otu004822","Otu001012","Otu002086","Otu0
02685","Otu000613","Otu006285","Otu000820","Otu003320","Otu005831","Otu001040","Otu001640",
"Otu001934","Otu006773","Otu001759","Otu000785","Otu002088","Otu000823","Otu001527","Otu00
3764","Otu001355","Otu001876","Otu003886","Otu002069","Otu008301","Otu002270","Otu024140","
Otu002122","Otu001644","Otu001878","Otu003334","Otu003827","Otu004693","Otu001590","Otu003
178","Otu006940","Otu001186","Otu002874","Otu002278","Otu004546","Otu002244","Otu007867","O
tu002110","Otu003053","Otu006512","Otu002862","Otu001097","Otu000941","Otu001235","Otu0022
54","Otu002074","Otu003678","Otu000817","Otu003610","Otu006579","Otu001936","Otu002934","Ot
u001078","Otu006625","Otu001669","Otu002286","Otu002551","Otu002927","Otu004983","Otu00563
5","Otu005735","Otu006585","Otu008100","Otu008375","Otu011970","Otu012267","Otu012378","Otu
012487","Otu012796","Otu013386","Otu013497","Otu026010","Otu001345","Otu005377","Otu004051
","Otu003846","Otu003530","Otu005240","Otu005178","Otu002191","Otu002936","Otu003738","Otu0
03731","Otu004537","Otu011269","Otu004690","Otu003477","Otu006098","Otu007219","Otu013061",
"Otu006380","Otu003210","Otu006586","Otu006835","Otu002892","Otu007438","Otu007537","Otu00
7877","Otu008994","Otu015437","Otu021286","Otu030657","Otu002202","Otu002656","Otu001007","
Otu003104","Otu012840","Otu004713","Otu004699","Otu003116","Otu000606","Otu003683","Otu003
020","Otu007440","Otu004061","Otu004100","Otu005432","Otu011986","Otu001507","Otu001781","O
tu004004","Otu005354","Otu005434","Otu012302","Otu001301","Otu002336","Otu004336","Otu0025
54","Otu003538","Otu013536","Otu008454","Otu003074","Otu002185","Otu002813","Otu004334","Ot
u006508","Otu008303","Otu002233","Otu004768","Otu003654","Otu004531","Otu001150","Otu00243
4","Otu002978","Otu003134","Otu003151","Otu003450","Otu003455","Otu003818","Otu003916","Otu
004083","Otu004163","Otu004211","Otu004234","Otu004251","Otu004261","Otu004302","Otu004335
","Otu004374","Otu004406","Otu004631","Otu004702","Otu005013","Otu005374","Otu005446","Otu0
05547","Otu005603","Otu006051","Otu006078","Otu006145","Otu006745","Otu006826","Otu006862",
"Otu006987","Otu007047","Otu007120","Otu007300","Otu007324","Otu007472","Otu007510","Otu00
7528","Otu007547","Otu007763","Otu007766","Otu007767","Otu007778","Otu008285","Otu008302","
Otu008525","Otu008584","Otu008611","Otu008668","Otu008880","Otu008953","Otu008984","Otu009
022","Otu009317","Otu009641","Otu009689","Otu009713","Otu009753","Otu009862","Otu009908","O
tu010061","Otu010332","Otu010718","Otu011401","Otu011409","Otu011662","Otu011795","Otu0119
92","Otu012118","Otu012171","Otu012494","Otu012496","Otu012677","Otu013395","Otu013581","Ot
```

```
u013618","Otu013688","Otu013963","Otu014325","Otu014464","Otu014507","Otu014905","Otu01496
4","Otu015954","Otu016384","Otu016751","Otu016858","Otu016879","Otu017179","Otu017462","Otu
018197","Otu018633","Otu019079","Otu019205","Otu019713","Otu019890","Otu020007","Otu020311
","Otu020381","Otu020439","Otu020522","Otu020537","Otu021171","Otu021727","Otu021934","Otu0
22008","Otu023417","Otu023420","Otu023681","Otu025394","Otu025430","Otu025699","Otu025721",
"Otu027655","Otu028075","Otu028228","Otu030143","Otu030720","Otu031864","Otu033370","Otu03
3768","Otu003245","Otu002766","Otu002886","Otu002414","Otu002678","Otu001492","Otu003031","
Otu003728","Otu003029","Otu002426","Otu004166","Otu000428","Otu005664","Otu001390","Otu003
325","Otu009979","Otu004737","Otu008979","Otu004329","Otu006638","Otu011384","Otu002792","O
tu005727","Otu007451","Otu004043","Otu009296","Otu005994","Otu007538","Otu020898","Otu0027
23","Otu003894","Otu006240","Otu001493","Otu013985","Otu018786","Otu003727","Otu006632","Ot
u003408","Otu002479","Otu007571","Otu010106","Otu010185","Otu010472","Otu003964","Otu00516
9","Otu014317","Otu002265","Otu004962","Otu009234","Otu003635","Otu006280",
"Otu003550","Otu005750","Otu016062")
indicatortext<-indicator1$text
test<-colSums(indicator1[6:368])
summary(test)

indicatorpd<-indicator1$pd
indicatorsite<-indicator1$site
rowSums(indicator1[6:368])
indicatorabund<-rowSums(indicator1[6:368])
boxplot(indicatorabund~indicatorpd)

plotNormalHistogram(indicatorabund[indicatorpd=="1"])
plotNormalHistogram(indicatorabund[indicatorpd=="0"])
logabund<-log(indicatorabund+1)
plotNormalHistogram(logabund[indicatorpd=="1"])
plotNormalHistogram(logabund[indicatorpd=="0"])
shapiro.test(logabund[indicatorpd=="1"])
shapiro.test(logabund[indicatorpd=="0"])
bartlett.test(indicatorabund~indicatorpd)
#GLMM compairason of indicator abunadnce between +/- bats
m1<-glmer(indicatorabund~indicatorpd+(1|indicatorsite), family=poisson)
op<-par(mfrow=c(1,2))
qqnorm(resid(m1))
qqline(resid(m1))
plot(residuals(m1)~fitted(m1), main="residuals vs fitted")
op<-par(mfrow=c(1,1))
summary(m1)
m2<-glmer.nb(indicatorabund~indicatorpd+(1|indicatorsite), data=indicator1, nAGQ = 25,
	control=glmerControl(optimizer="bobyqa"))#Produces singular fit

boxplot(indicatorabund~indicatorpd, data=indicator1, xlab = "Pd status", ylab = "Pd (-) Indicator Taxa
Abundance")
stripchart(indicatorabund~indicatorpd, data=indicator1,vertical=TRUE, method="jitter", add=TRUE,
pch=20, col="black")
indicatortext<-indicator1$text
```

```
p<-ggplot(indicator1, aes(x=indicatortext, y=indicatorabund, group=text))+
  geom_boxplot()+
  labs(x="P. destructans Status", y="P. destructans (-) Indicator Taxa Abundance", caption = "A" )+
  theme( plot.caption = element_text(size=28, face="bold"),
        #panel.border = element_blank(),
        #panel.grid.major = element_blank(),
        #panel.grid.minor=element_blank(),
        panel.background  = element_blank(),
        axis.line=element_line(size=0.5, colour="black"))

p

#Copy # analysis
indicatorcopiesneg<-subset(indicator1, pd=="1", header=T)
logcopiesindicatorneg<-log(indicatorcopiesneg$copies+1)
indicatorcopiesnegabund<-rowSums(indicatorcopiesneg[6:368])
indicatorcopiesnegsite<-indicatorcopiesneg$site
plot(logcopiesindicatorneg, indicatorcopiesnegabund)

m4<-glmer(indicatorcopiesnegabund~logcopiesindicatorneg+(1|indicatorcopiesnegsite), family =
poisson)
op<-par(mfrow=c(1,2))
qqnorm(resid(m4))
qqline(resid(m4))
plot(residuals(m4)~fitted(m4), main="residuals vs fitted")
op<-par(mfrow=c(1,1))
summary(m4)

m4nb<-glmer.nb(indicatorcopiesnegabund~logcopiesindicatorneg+(1|indicatorcopiesnegsite),
data=indicator1)
#Singular fit

indicatorcopiesnegdata<-indicatorcopiesneg[6:368]
B1 <- glm(indicatorcopiesnegabund~logcopiesindicatorneg, family=poisson,
data=indicatorcopiesnegdata)
summary(B1)
plot(indicatorcopiesnegabund~logcopiesindicatorneg, data=indicatorcopiesnegdata, type='n', bty="n",
xlim=c(0,10), ylim=c(0,275),xlab="(Log) Fungal Load", ylab="Indicator Abundance")
points(logcopiesindicatorneg, indicatorcopiesnegabund, pch=19, cex=1.5, col="black", bg="black")
axis(side=1, lwd=2)
axis(side=2, lwd=2)
title("B", adj=1, line=-26, cex.main=2)
pred <-seq (0,10.4,0.01)
B1.pred <- predict(B1, list(logcopiesindicatorneg=pred), type="response", data=indicatorcopiesnegdata)
lines(pred, B1.pred, lty=1, col="blue", lwd=5)
```

```
####end####
##################################################################
#Distance Decay
##################################################################
####DDR####
##
#Positive
##
betapart.core_pos<-betapart.core(PA_pos)
pair_pos<-beta.pair(betapart.core_pos, index.family="sorensen")

SOR_pos<-(pair_pos$beta.sor)
SIM_pos<-(pair_pos$beta.sim)
SNE_pos<-(pair_pos$beta.sne)

logSOR_pos<-log(SOR_pos+1)
logSIM_pos<-log(SIM_pos+1)
logSNE_pos<-log(SNE_pos+1)

geo_pos = data.frame(positive$Longitude, positive$Latitude)
posdist<-dist(geo_pos, method="euclidean")
logposdist<-log(posdist+1)

plot(posdist, SOR_pos)
plot(logposdist, SOR_pos)

#Make dataframe for modeling approach - need data in columns
#posdf<-as.matrix(SIM_pos)
#posdf[upper.tri(posdf)] <- NA
#SIM_posmelt<-melt(posdf)
#write.csv(SIM_posmelt, file="200612_SIM_posmelt.csv")#Make sure to relabel columns-relabel so that
#the first column is ‘g’
#posdfSOR<-as.matrix(SOR_pos)
#posdfSOR[upper.tri(posdfSOR)] <- NA
#SOR_posmelt<-melt(posdfSOR)
#write.csv(SOR_posmelt, file="200612_SOR_posmelt.csv")
#posdfSNE<-as.matrix(SNE_pos)
#posdfSNE[upper.tri(posdfSNE)] <- NA
#SNE_posmelt<-melt(posdfSNE)
#write.csv(SNE_posmelt, file="200612_SNE_posmelt.csv")

#posdistdf<-as.matrix(posdist)
#posdistdf[upper.tri(posdistdf)] <- NA
#rownames(posdistdf)<-positive$Group
#colnames(posdistdf)<-positive$Group
#posdistmelt<-melt(posdistdf)
#write.csv(posdistmelt, file="200713_posdistmelttest.csv")
#GeoPos<-read.csv("200612_posdistmelt.csv")
```

```
#SIMPos<-read.csv("200612_SIM_posmelt.csv")
#SORPos<-read.csv("200612_SOR_posmelt.csv")
#SNEPos<-read.csv("200612_SNE_posmelt.csv")
#posSIM_Geo<-merge(GeoPos,SIMPos, by="g")
#posSOR_Geo<-merge(SORPos, SNEPos, by="g")
#totalpos<-merge(posSIM_Geo, posSOR_Geo, by="g")
#write.csv(totalpos, file="200612_totalgeodist.csv")

##
#Negative
##
betapart.core_neg<-betapart.core(PA_neg)
pair_neg<-beta.pair(betapart.core_neg, index.family="sor")
SOR_neg<-(pair_neg$beta.sor)
SIM_neg<-(pair_neg$beta.sim)
SNE_neg<-(pair_neg$beta.sne)

logSOR_neg<-log(SOR_neg+1)
logSIM_neg<-log(SIM_neg+1)
logSNE_neg<-log(SNE_neg+1)

geo_neg = data.frame(negative$Longitude, negative$Latitude)
rownames(geo_neg)<-negative$Group
negdist<-dist(geo_neg, method="euclidean") #1 ~~ 100km
lognegdist<-log(negdist+1)

#SIM_negdf<-as.matrix(SIM_neg)
#SIM_negdf[upper.tri(SIM_negdf)] <- NA
#SIM_negmelt<-melt(SIM_negdf)
#write.csv(SIM_negmelt, file="200612_SIM_negmelt.csv")#Make sure to relabel columns-relabel so that
#the first column is g
#SOR_negdf<-as.matrix(SOR_neg)
#SOR_negdf[upper.tri(SOR_negdf)] <- NA
#SOR_negmelt<-melt(SOR_negdf)
#write.csv(SOR_negmelt, file="200612_SOR_negmelt.csv")
#SNE_negdf<-as.matrix(SNE_neg)
#SNE_negdf[upper.tri(SNE_negdf)] <- NA
#SNE_negmelt<-melt(SNE_negdf)
#write.csv(SNE_negmelt, file="200612_SNE_negmelt.csv")
#negdistdf<-as.matrix(negdist)
#negdistdf[upper.tri(negdistdf)] <- NA
#negdistmelt<-melt(negdistdf)
#rownames(negdistdf)<-negative$Group
#colnames(negdistdf)<-negative$Group
#write.csv(negdistmelt, file="200612_Negdistmelt.csv")

#GeoNeg<-read.csv("200612_Negdistmelt.csv")
#SIMneg<-read.csv("200612_SIM_negmelt.csv")
```

```
#SORneg<-read.csv("200612_SOR_negmelt.csv")
#SNEneg<-read.csv("200612_SNE_negmelt.csv")

#negSIM_Geo<-merge(GeoNeg,SIMneg, by="g")
#negSOR_Geo<-merge(SORneg,SNEneg, by="g")
#totalneg<-merge(negSIM_Geo, negSOR_Geo, by="g")
#write.csv(totalneg, file="200612_totalneg_geodist.csv")

#df <- read.csv("200612_Sim_bygeo.csv", header = TRUE, row.names = NULL)

#dist.unique<-unique(df$GeoDist2)
#write.csv(dist.unique, file = "test2.csv")
####Site contrasts ####
df %>%
  mutate(contrastID = case_when(
    GeoDist==0~"1",
    GeoDist==1.271903372~"2",
    GeoDist==0.85368171~"3",
    GeoDist==0.740304456~"4",
    GeoDist==1.153182792~"5",
    GeoDist==1.313440762~"6",
    GeoDist==0.407680362~"7",
    GeoDist==2.431202204~"8",
    GeoDist==0.505322856~"9",
    GeoDist==1.284026106~"10",
    GeoDist==1.465905806~"11",
    GeoDist==1.425243087~"12",
    GeoDist==0.877550124~"13",
    GeoDist==1.236631568~"14",
    GeoDist==1.029505762~"15",
    GeoDist==1.002261517~"16",
    GeoDist==1.010503693~"17",
    GeoDist==0.964223147~"18",
    GeoDist==1.398601493~"19",
    GeoDist==0.9145334~"20",
    GeoDist==1.110908381~"21",
    GeoDist==1.59397345~"22",
    GeoDist==1.700349417~"23",
    GeoDist==0.341663772~"24",
    GeoDist==0.459780738~"25",
    GeoDist==0.976872935~"26",
    GeoDist==1.16087469~"27",
    GeoDist==1.889791076~"28",
    GeoDist==1.563729516~"29",
    GeoDist==0.377409325~"30",
    GeoDist==0.166063942~"31",
    GeoDist==1.14069024~"32",
    GeoDist==0.401233338~"33",
```

GeoDist==0.469186449~"34",
GeoDist==0.336467093~"35",
GeoDist==0.51999389~"36",
GeoDist==1.135025435~"37",
GeoDist==1.134794724~"38",
GeoDist==0.482234183~"39",
GeoDist==0.51555363~"40",
GeoDist==0.766645657~"41",
GeoDist==1.233017438~"42",
GeoDist==2.216942632~"43",
GeoDist==1.031671489~"44",
GeoDist==0.541554476~"45",
GeoDist==1.251644424~"46",
GeoDist==0.728677246~"47",
GeoDist==1.247097729~"48",
GeoDist==3.57170676~"49",
GeoDist==1.776716236~"50",
GeoDist==0.786130753~"51",
GeoDist==2.438924313~"52",
GeoDist==2.640902005~"53",
GeoDist==1.616218665~"54",
GeoDist==2.357506619~"55",
GeoDist==0.745910787~"56",
GeoDist==0.786406682~"57",
GeoDist==0.776060382~"58",
GeoDist==0.809592161~"59",
GeoDist==2.612321942~"60",
GeoDist==1.828934569~"61",
GeoDist==2.182943807~"62",
GeoDist==2.817564725~"63",
GeoDist==2.850846701~"64",
GeoDist==1.319866302~"65",
GeoDist==0.812270325~"66",
GeoDist==0.476932273~"67",
GeoDist==1.161776894~"68",
GeoDist==3.079460617~"69",
GeoDist==2.787954104~"70",
GeoDist==1.397579778~"71",
GeoDist==1.382913362~"72",
GeoDist==0.755686683~"73",
GeoDist==0.984849232~"74",
GeoDist==1.74107356~"75",
GeoDist==1.325191915~"76",
GeoDist==0.751950205~"77",
GeoDist==1.170443216~"78",
GeoDist==1.169817308~"79",
GeoDist==1.269541037~"80",
GeoDist==0.996404482~"81",

GeoDist==1.120920451~"82",
GeoDist==0.308193063~"83",
GeoDist==0.945320786~"84",
GeoDist==0.802054431~"85",
GeoDist==1.706852972~"86",
GeoDist==1.522216348~"87",
GeoDist==1.162886638~"88",
GeoDist==3.240492136~"89",
GeoDist==1.236838722~"90",
GeoDist==1.537375075~"91",
GeoDist==2.319575727~"92",
GeoDist==2.20463378~"93",
GeoDist==1.6706015~"94",
GeoDist==2.075592877~"95",
GeoDist==1.334174213~"96",
GeoDist==1.339083638~"97",
GeoDist==1.338823605~"98",
GeoDist==1.324493267~"99",
GeoDist==2.1809561~"100",
GeoDist==1.755662278~"101",
GeoDist==1.961552143~"102",
GeoDist==2.358408059~"103",
GeoDist==2.517460799~"104",
GeoDist==1.148609515~"105",
GeoDist==0.6811341~"106",
GeoDist==1.095467305~"107",
GeoDist==1.665888573~"108",
GeoDist==2.678154814~"109",
GeoDist==2.3284851~"110",
GeoDist==1.20712953~"111",
GeoDist==1.019550261~"112",
GeoDist==1.422396584~"113",
GeoDist==0.952102163~"114",
GeoDist==1.189772992~"115",
GeoDist==0.623306924~"116",
GeoDist==0.684182945~"117",
GeoDist==1.653082016~"118",
GeoDist==1.652600224~"119",
GeoDist==0.463631356~"120",
GeoDist==0.346626222~"121",
GeoDist==0.132370109~"122",
GeoDist==1.20618611~"123",
GeoDist==1.834944586~"124",
GeoDist==0.958348196~"125",
GeoDist==0.730815489~"126",
GeoDist==0.720608595~"127",
GeoDist==3.046361466~"128",
GeoDist==1.241800742~"129",

GeoDist==0.738676899~"130",
GeoDist==1.942257592~"131",
GeoDist==2.101053879~"132",
GeoDist==1.144756753~"133",
GeoDist==1.82960507~"134",
GeoDist==0.534038062~"135",
GeoDist==0.543398537~"136",
GeoDist==0.541670302~"137",
GeoDist==0.534923562~"138",
GeoDist==2.072612284~"139",
GeoDist==1.329273545~"140",
GeoDist==1.661557752~"141",
GeoDist==2.277223305~"142",
GeoDist==2.320914402~"143",
GeoDist==0.784450002~"144",
GeoDist==0.290105293~"145",
GeoDist==0.307332707~"146",
GeoDist==0.898667097~"147",
GeoDist==2.543755234~"148",
GeoDist==2.247526549~"149",
GeoDist==0.862549846~"150",
GeoDist==0.8425968~"151",
GeoDist==0.620369135~"152",
GeoDist==0.449898611~"153",
GeoDist==1.208144917~"154",
GeoDist==0.853700246~"155",
GeoDist==0.231793249~"156",
GeoDist==0.892175891~"157",
GeoDist==0.891621576~"158",
GeoDist==0.846780341~"159",
GeoDist==0.598288301~"160",
GeoDist==0.831887407~"161",
GeoDist==0.510064177~"162",
GeoDist==1.484650426~"163",
GeoDist==0.641326688~"164",
GeoDist==0.767652536~"165",
GeoDist==2.613450267~"166",
GeoDist==1.441690879~"167",
GeoDist==0.564785632~"168",
GeoDist==1.410984598~"169",
GeoDist==1.903913356~"170",
GeoDist==0.647344754~"171",
GeoDist==1.507189912~"172",
GeoDist==0.505765528~"173",
GeoDist==0.467055337~"174",
GeoDist==0.476557953~"175",
GeoDist==0.451015762~"176",
GeoDist==1.875125575~"177",

GeoDist==0.90961362~"178",
GeoDist==1.310379212~"179",
GeoDist==2.076238959~"180",
GeoDist==1.967177528~"181",
GeoDist==0.868677022~"182",
GeoDist==1.030086378~"183",
GeoDist==0.79036764~"184",
GeoDist==0.10295182~"185",
GeoDist==2.237203174~"186",
GeoDist==2.05183139~"187",
GeoDist==0.895763989~"188",
GeoDist==1.099843883~"189",
GeoDist==0.514740437~"190",
GeoDist==0.80474225~"191",
GeoDist==1.438282294~"192",
GeoDist==1.462239289~"193",
GeoDist==1.022670193~"194",
GeoDist==0.083028479~"195",
GeoDist==0.083643073~"196",
GeoDist==1.556627321~"197",
GeoDist==1.409057727~"198",
GeoDist==1.695681726~"199",
GeoDist==0.971278002~"200",
GeoDist==1.974802208~"201",
GeoDist==1.062264256~"202",
GeoDist==3.215234048~"203",
GeoDist==1.74871363~"204",
GeoDist==0.076814033~"205",
GeoDist==2.020748402~"206",
GeoDist==2.416751496~"207",
GeoDist==1.204750267~"208",
GeoDist==2.054876971~"209",
GeoDist==0.291333197~"210",
GeoDist==0.330083722~"211",
GeoDist==0.31860752~"212",
GeoDist==0.374081583~"213",
GeoDist==2.387310202~"214",
GeoDist==1.461894232~"215",
GeoDist==1.860707935~"216",
GeoDist==2.594822404~"217",
GeoDist==2.537570467~"218",
GeoDist==1.16778086~"219",
GeoDist==0.966164021~"220",
GeoDist==0.427187547~"221",
GeoDist==0.538478673~"222",
GeoDist==2.796938409~"223",
GeoDist==2.56781213~"224",
GeoDist==1.229152336~"225",

GeoDist==1.34251454~"226",
GeoDist==0.186563122~"227",
GeoDist==0.914313194~"228",
GeoDist==1.728862072~"229",
GeoDist==1.528854222~"230",
GeoDist==0.923624561~"231",
GeoDist==0.561137447~"232",
GeoDist==0.560580317~"233",
GeoDist==1.556111351~"234",
GeoDist==1.324273485~"235",
GeoDist==1.561515228~"236",
GeoDist==0.420564816~"237",
GeoDist==1.33509152~"238",
GeoDist==2.326772881~"239",
GeoDist==0.700678169~"240",
GeoDist==1.014923773~"241",
GeoDist==1.234437778~"242",
GeoDist==1.40745183~"243",
GeoDist==0.507720679~"244",
GeoDist==1.110895216~"245",
GeoDist==0.772005426~"246",
GeoDist==0.732303045~"247",
GeoDist==0.743657836~"248",
GeoDist==0.688620099~"249",
GeoDist==1.378322912~"250",
GeoDist==0.627431088~"251",
GeoDist==0.940952619~"252",
GeoDist==1.586060483~"253",
GeoDist==1.603826619~"254",
GeoDist==0.10845097~"255",
GeoDist==0.549868749~"256",
GeoDist==0.835049024~"257",
GeoDist==0.791730694~"258",
GeoDist==1.834483096~"259",
GeoDist==1.557179655~"260",
GeoDist==0.168130201~"261",
GeoDist==0.332193305~"262",
GeoDist==0.876551794~"263",
GeoDist==0.274564945~"264",
GeoDist==0.688716043~"265",
GeoDist==0.740720122~"266",
GeoDist==0.589139644~"267",
GeoDist==0.762619727~"268",
GeoDist==0.762496136~"269",
GeoDist==0.870681213~"270",
GeoDist==0.818525548~"271",
GeoDist==1.10431629~"272",
GeoDist==1.110782182~"273",

GeoDist==2.16949064~"274",
GeoDist==2.005506117~"275",
GeoDist==3.144499556~"276",
GeoDist==1.202767834~"277",
GeoDist==1.045719284~"278",
GeoDist==2.012695867~"279",
GeoDist==1.216987717~"280",
GeoDist==2.971650227~"281",
GeoDist==2.923054939~"282",
GeoDist==2.936304999~"283",
GeoDist==2.881759218~"284",
GeoDist==1.066452383~"285",
GeoDist==1.764513579~"286",
GeoDist==1.389153923~"287",
GeoDist==0.919394814~"288",
GeoDist==0.73161459~"289",
GeoDist==2.264510024~"290",
GeoDist==2.834788898~"291",
GeoDist==3.13260012~"292",
GeoDist==2.710319427~"293",
GeoDist==0.580020515~"294",
GeoDist==0.946240645~"295",
GeoDist==2.186579536~"296",
GeoDist==2.274324111~"297",
GeoDist==3.043910811~"298",
GeoDist==2.596768695~"299",
GeoDist==2.052823403~"300",
GeoDist==2.618519807~"301",
GeoDist==2.886459929~"302",
GeoDist==2.680594995~"303",
GeoDist==2.681025627~"304",
GeoDist==2.785176254~"305",
GeoDist==2.932342473~"306",
GeoDist==3.125923628~"307",
GeoDist==3.401897272~"308",
GeoDist==4.474073447~"309",
GeoDist==1.707189656~"310",
GeoDist==1.21444534~"311",
GeoDist==0.967952012~"312",
GeoDist==0.943187679~"313",
GeoDist==0.886296062~"314",
GeoDist==1.457536879~"315",
GeoDist==1.421838839~"316",
GeoDist==1.43226328~"317",
GeoDist==1.379417315~"318",
GeoDist==0.944126772~"319",
GeoDist==0.832435325~"320",
GeoDist==0.828551446~"321",

GeoDist==1.124914596~"322",
GeoDist==1.288800999~"323",
GeoDist==0.592241945~"324",
GeoDist==0.964973662~"325",
GeoDist==1.456857493~"326",
GeoDist==1.480483854~"327",
GeoDist==1.441347134~"328",
GeoDist==1.094782485~"329",
GeoDist==0.54896562~"330",
GeoDist==0.412364965~"331",
GeoDist==1.56589945~"332",
GeoDist==0.850934089~"333",
GeoDist==0.047350803~"334",
GeoDist==0.613688084~"335",
GeoDist==1.025074427~"336",
GeoDist==1.449652682~"337",
GeoDist==1.449610188~"338",
GeoDist==0.779693468~"339",
GeoDist==0.945952473~"340",
GeoDist==1.120508855~"341",
GeoDist==1.722485878~"342",
GeoDist==2.721946553~"343",
GeoDist==1.948514933~"344",
GeoDist==2.355523942~"345",
GeoDist==1.135855916~"346",
GeoDist==1.989332733~"347",
GeoDist==0.254525895~"348",
GeoDist==0.285642571~"349",
GeoDist==0.27579855~"350",
GeoDist==0.327775936~"351",
GeoDist==2.326106897~"352",
GeoDist==1.394486318~"353",
GeoDist==1.794530505~"354",
GeoDist==2.533219087~"355",
GeoDist==2.470149569~"356",
GeoDist==1.121676207~"357",
GeoDist==0.957560105~"358",
GeoDist==0.444136563~"359",
GeoDist==0.461891976~"360",
GeoDist==2.730936406~"361",
GeoDist==2.506460142~"362",
GeoDist==1.180478642~"363",
GeoDist==1.304459841~"364",
GeoDist==0.143379302~"365",
GeoDist==0.883069683~"366",
GeoDist==1.688981804~"367",
GeoDist==1.512626554~"368",
GeoDist==0.918648007~"369",

GeoDist==0.484911034~"370",
GeoDist==0.484359203~"371",
GeoDist==1.548211865~"372",
GeoDist==1.324444343~"373",
GeoDist==1.570433067~"374",
GeoDist==0.478570777~"375",
GeoDist==1.411903641~"376",
GeoDist==0.766581449~"377",
GeoDist==0.83172713~"378",
GeoDist==0.404574939~"379",
GeoDist==1.789869703~"380",
GeoDist==1.741043977~"381",
GeoDist==1.754234876~"382",
GeoDist==1.701610184~"383",
GeoDist==0.74904653~"384",
GeoDist==0.613055534~"385",
GeoDist==0.385987091~"386",
GeoDist==0.877308772~"387",
GeoDist==0.620589047~"388",
GeoDist==1.203405273~"389",
GeoDist==1.780711337~"390",
GeoDist==1.978488037~"391",
GeoDist==1.508496537~"392",
GeoDist==0.896976154~"393",
GeoDist==0.866433455~"394",
GeoDist==1.132309716~"395",
GeoDist==1.300174716~"396",
GeoDist==1.854591491~"397",
GeoDist==1.50776415~"398",
GeoDist==1.255034209~"399",
GeoDist==1.74492339~"400",
GeoDist==1.822934487~"401",
GeoDist==1.47915676~"402",
GeoDist==1.47960015~"403",
GeoDist==1.914935889~"404",
GeoDist==1.978638224~"405",
GeoDist==2.229122204~"406",
GeoDist==2.238492708~"407",
GeoDist==3.307348869~"408",
GeoDist==1.256658636~"409",
GeoDist==0.424894247~"410",
GeoDist==2.141400392~"411",
GeoDist==2.095586338~"412",
GeoDist==2.108384487~"413",
GeoDist==2.051503555~"414",
GeoDist==0.029456655~"415",
GeoDist==0.996258452~"416",
GeoDist==0.608595845~"417",

GeoDist==0.179027712~"418",
GeoDist==0.38151491~"419",
GeoDist==1.323716454~"420",
GeoDist==1.858793307~"421",
GeoDist==2.242221867~"422",
GeoDist==1.983880462~"423",
GeoDist==0.473699992~"424",
GeoDist==0.151149783~"425",
GeoDist==1.247173828~"426",
GeoDist==1.279934336~"427",
GeoDist==2.23253424~"428",
GeoDist==1.656981983~"429",
GeoDist==1.014911736~"430",
GeoDist==1.581338684~"431",
GeoDist==1.914894603~"432",
GeoDist==1.951094644~"433",
GeoDist==1.951355315~"434",
GeoDist==1.745365968~"435",
GeoDist==1.908492334~"436",
GeoDist==2.086893339~"437",
GeoDist==2.517913643~"438",
GeoDist==3.574387635~"439",
GeoDist==0.863139413~"440",
GeoDist==0.960605794~"441",
GeoDist==0.911839658~"442",
GeoDist==0.925077407~"443",
GeoDist==0.871493558~"444",
GeoDist==1.227921213~"445",
GeoDist==0.263555892~"446",
GeoDist==0.666310432~"447",
GeoDist==1.42894965~"448",
GeoDist==1.335529661~"449",
GeoDist==0.538244543~"450",
GeoDist==1.038667487~"451",
GeoDist==1.149621026~"452",
GeoDist==0.730006118~"453",
GeoDist==1.600018309~"454",
GeoDist==1.404488882~"455",
GeoDist==0.503557709~"456",
GeoDist==0.744653478~"457",
GeoDist==1.031291581~"458",
GeoDist==0.748193035~"459",
GeoDist==0.956798081~"460",
GeoDist==1.211960316~"461",
GeoDist==1.068145305~"462",
GeoDist==0.69760636~"463",
GeoDist==0.697909901~"464",
GeoDist==1.358378774~"465",

GeoDist==1.326056323~"466",
GeoDist==1.610330888~"467",
GeoDist==1.407018832~"468",
GeoDist==2.475698895~"469",
GeoDist==1.791783621~"470",
GeoDist==1.74423104~"471",
GeoDist==1.757356682~"472",
GeoDist==1.701140174~"473",
GeoDist==0.399847805~"474",
GeoDist==0.599633475~"475",
GeoDist==0.196917975~"476",
GeoDist==0.580697864~"477",
GeoDist==0.494133804~"478",
GeoDist==1.047633173~"479",
GeoDist==1.620730737~"480",
GeoDist==1.927741539~"481",
GeoDist==1.592759535~"482",
GeoDist==0.742359849~"483",
GeoDist==0.560010745~"484",
GeoDist==0.969777832~"485",
GeoDist==1.073963045~"486",
GeoDist==1.874808405~"487",
GeoDist==1.37987817~"488",
GeoDist==0.931328583~"489",
GeoDist==1.469029108~"490",
GeoDist==1.671281825~"491",
GeoDist==1.56050811~"492",
GeoDist==1.560822104~"493",
GeoDist==1.640458374~"494",
GeoDist==1.749272882~"495",
GeoDist==1.971861251~"496",
GeoDist==2.200544153~"497",
GeoDist==3.270200202~"498",
GeoDist==0.048825736~"499",
GeoDist==0.035656295~"500",
GeoDist==0.090643716~"501",
GeoDist==2.111945154~"502",
GeoDist==1.20839196~"503",
GeoDist==1.600399485~"504",
GeoDist==2.320075383~"505",
GeoDist==2.280010983~"506",
GeoDist==0.876946435~"507",
GeoDist==0.718244719~"508",
GeoDist==0.293462828~"509",
GeoDist==0.418213219~"510",
GeoDist==2.533872383~"511",
GeoDist==2.292547254~"512",
GeoDist==0.939399681~"513",

GeoDist==1.052263041~"514",
GeoDist==0.111195641~"515",
GeoDist==0.628555469~"516",
GeoDist==1.438051753~"517",
GeoDist==1.263216606~"518",
GeoDist==0.684930433~"519",
GeoDist==0.424951379~"520",
GeoDist==0.424326415~"521",
GeoDist==1.30660578~"522",
GeoDist==1.094824359~"523",
GeoDist==1.354312224~"524",
GeoDist==0.473648934~"525",
GeoDist==1.521347964~"526",
GeoDist==0.013250151~"527",
GeoDist==0.044369653~"528",
GeoDist==2.066129731~"529",
GeoDist==1.160005534~"530",
GeoDist==1.552561163~"531",
GeoDist==2.274170836~"532",
GeoDist==2.232064809~"533",
GeoDist==0.838165294~"534",
GeoDist==0.709401733~"535",
GeoDist==0.324853283~"536",
GeoDist==0.383687436~"537",
GeoDist==2.48641659~"538",
GeoDist==2.246735954~"539",
GeoDist==0.899073744~"540",
GeoDist==1.018877476~"541",
GeoDist==0.144399979~"542",
GeoDist==0.601065366~"543",
GeoDist==1.403380562~"544",
GeoDist==1.245055694~"545",
GeoDist==0.679656085~"546",
GeoDist==0.387749893~"547",
GeoDist==0.387129765~"548",
GeoDist==1.294446619~"549",
GeoDist==1.089800022~"550",
GeoDist==1.354467982~"551",
GeoDist==0.519514851~"552",
GeoDist==1.569948301~"553",
GeoDist==0.056905081~"554",
GeoDist==2.078928005~"555",
GeoDist==1.173243727~"556",
GeoDist==1.565731513~"557",
GeoDist==2.286988291~"558",
GeoDist==2.245251951~"559",
GeoDist==0.849306935~"560",
GeoDist==0.712701825~"561",

GeoDist==0.316869688~"562",
GeoDist==0.391818077~"563",
GeoDist==2.499527587~"564",
GeoDist==2.259534269~"565",
GeoDist==0.910597514~"566",
GeoDist==1.028692948~"567",
GeoDist==0.133642958~"568",
GeoDist==0.609270374~"569",
GeoDist==1.413548863~"570",
GeoDist==1.250962255~"571",
GeoDist==0.68198234~"572",
GeoDist==0.396729357~"573",
GeoDist==0.396107275~"574",
GeoDist==1.298772735~"575",
GeoDist==1.092186626~"576",
GeoDist==1.355499965~"577",
GeoDist==0.507488551~"578",
GeoDist==1.5569359~"579",
GeoDist==2.022047163~"580",
GeoDist==1.118161381~"581",
GeoDist==1.5097858~"582",
GeoDist==2.230117626~"583",
GeoDist==2.189426127~"584",
GeoDist==0.794736792~"585",
GeoDist==0.684649038~"586",
GeoDist==0.340115078~"587",
GeoDist==0.374226723~"588",
GeoDist==2.443235198~"589",
GeoDist==2.202653281~"590",
GeoDist==0.855190218~"591",
GeoDist==0.977568721~"592",
GeoDist==0.187943434~"593",
GeoDist==0.56338169~"594",
GeoDist==1.361388278~"595",
GeoDist==1.212603435~"596",
GeoDist==0.657858174~"597",
GeoDist==0.374567603~"598",
GeoDist==0.373960895~"599",
GeoDist==1.266210513~"600",
GeoDist==1.067261149~"601",
GeoDist==1.335830289~"602",
GeoDist==0.551252933~"603",
GeoDist==1.607615156~"604",
GeoDist==0.967784297~"605",
GeoDist==0.581531788~"606",
GeoDist==0.208413073~"607",
GeoDist==0.390690891~"608",
GeoDist==1.294849736~"609",

GeoDist==1.831071603~"610",
GeoDist==2.213039177~"611",
GeoDist==1.954898842~"612",
GeoDist==0.497239441~"613",
GeoDist==0.180606302~"614",
GeoDist==1.218237852~"615",
GeoDist==1.25259091~"616",
GeoDist==2.203083203~"617",
GeoDist==1.62826623~"618",
GeoDist==0.991186946~"619",
GeoDist==1.557725307~"620",
GeoDist==1.887054591~"621",
GeoDist==1.922102139~"622",
GeoDist==1.92236128~"623",
GeoDist==1.722290869~"624",
GeoDist==1.883369444~"625",
GeoDist==2.063726219~"626",
GeoDist==2.488722476~"627",
GeoDist==3.545491992~"628",
GeoDist==0.402880439~"629",
GeoDist==1.167055105~"630",
GeoDist==1.075720946~"631",
GeoDist==0.611354526~"632",
GeoDist==1.177066939~"633",
GeoDist==1.374496061~"634",
GeoDist==0.993486355~"635",
GeoDist==1.337821359~"636",
GeoDist==1.143039605~"637",
GeoDist==0.548628219~"638",
GeoDist==0.756987843~"639",
GeoDist==1.285091882~"640",
GeoDist==0.89807746~"641",
GeoDist==0.857675891~"642",
GeoDist==1.229189691~"643",
GeoDist==1.216317019~"644",
GeoDist==0.961128184~"645",
GeoDist==0.961434495~"646",
GeoDist==1.390630115~"647",
GeoDist==1.409536703~"648",
GeoDist==1.678410688~"649",
GeoDist==1.64006398~"650",
GeoDist==2.712075655~"651",
GeoDist==0.772397676~"652",
GeoDist==0.679897006~"653",
GeoDist==0.887555445~"654",
GeoDist==1.467062009~"655",
GeoDist==1.745163769~"656",
GeoDist==1.395845837~"657",

GeoDist==0.936407858~"658",
GeoDist==0.750152072~"659",
GeoDist==0.811115361~"660",
GeoDist==0.945186226~"661",
GeoDist==1.681599352~"662",
GeoDist==1.213774786~"663",
GeoDist==0.869447387~"664",
GeoDist==1.371982878~"665",
GeoDist==1.514895717~"666",
GeoDist==1.363603097~"667",
GeoDist==1.363917943~"668",
GeoDist==1.543103541~"669",
GeoDist==1.626460587~"670",
GeoDist==1.864984694~"671",
GeoDist==2.016323208~"672",
GeoDist==3.087635405~"673",
GeoDist==0.3606545~"674",
GeoDist==1.501549324~"675",
GeoDist==2.032020398~"676",
GeoDist==2.420946194~"677",
GeoDist==2.157649944~"678",
GeoDist==0.339631322~"679",
GeoDist==0.030274755~"680",
GeoDist==1.425238011~"681",
GeoDist==1.45185097~"682",
GeoDist==2.410822468~"683",
GeoDist==1.834250206~"684",
GeoDist==1.171226044~"685",
GeoDist==1.735619627~"686",
GeoDist==2.088611987~"687",
GeoDist==2.124956969~"688",
GeoDist==2.125229511~"689",
GeoDist==1.896708445~"690",
GeoDist==2.069682125~"691",
GeoDist==2.23822047~"692",
GeoDist==2.696655581~"693",
GeoDist==3.752197537~"694",
GeoDist==1.537170109~"695",
GeoDist==2.104206531~"696",
GeoDist==2.421638542~"697",
GeoDist==2.057954517~"698",
GeoDist==0.277959482~"699",
GeoDist==0.368954118~"700",
GeoDist==1.459076424~"701",
GeoDist==1.542830837~"702",
GeoDist==2.359912164~"703",
GeoDist==1.871016513~"704",
GeoDist==1.33608678~"705",

GeoDist==1.898120557~"706",
GeoDist==2.15642098~"707",
GeoDist==2.026501961~"708",
GeoDist==2.026864604~"709",
GeoDist==2.066848808~"710",
GeoDist==2.204121982~"711",
GeoDist==2.405855334~"712",
GeoDist==2.694086~"713",
GeoDist==3.764184863~"714",
GeoDist==0.580717731~"715",
GeoDist==0.92482297~"716",
GeoDist==0.896754051~"717",
GeoDist==1.759955735~"718",
GeoDist==1.472294581~"719",
GeoDist==0.078122909~"720",
GeoDist==0.236184201~"721",
GeoDist==0.982540605~"722",
GeoDist==0.335239041~"723",
GeoDist==0.580688256~"724",
GeoDist==0.678007537~"725",
GeoDist==0.627410109~"726",
GeoDist==0.867130971~"727",
GeoDist==0.86702929~"728",
GeoDist==0.820404287~"729",
GeoDist==0.801985279~"730",
GeoDist==1.079046757~"731",
GeoDist==1.200017092~"732",
GeoDist==2.25068455~"733",
GeoDist==0.569515965~"734",
GeoDist==0.996597508~"735",
GeoDist==2.31310694~"736",
GeoDist==2.001975306~"737",
GeoDist==0.656120478~"738",
GeoDist==0.581307141~"739",
GeoDist==0.822499546~"740",
GeoDist==0.29091546~"741",
GeoDist==0.928862721~"742",
GeoDist==0.572229916~"743",
GeoDist==0.060437154~"744",
GeoDist==0.981027393~"745",
GeoDist==0.980582241~"746",
GeoDist==0.590695551~"747",
GeoDist==0.385004796~"748",
GeoDist==0.666092158~"749",
GeoDist==0.79841956~"750",
GeoDist==1.757174211~"751",
GeoDist==0.708518309~"752",
GeoDist==2.662163923~"753",

GeoDist==2.392134075~"754",
GeoDist==0.998621694~"755",
GeoDist==1.045055622~"756",
GeoDist==0.34657368~"757",
GeoDist==0.608869435~"758",
GeoDist==1.429057867~"759",
GeoDist==1.141707852~"760",
GeoDist==0.519067638~"761",
GeoDist==0.712181496~"762",
GeoDist==0.711564387~"763",
GeoDist==1.149014074~"764",
GeoDist==0.905336995~"765",
GeoDist==1.134885723~"766",
GeoDist==0.275733583~"767",
GeoDist==1.342621985~"768",
GeoDist==2.326367173~"769",
GeoDist==2.132714173~"770",
GeoDist==0.931653418~"771",
GeoDist==1.121868089~"772",
GeoDist==0.416778849~"773",
GeoDist==0.793162269~"774",
GeoDist==1.473976448~"775",
GeoDist==1.458436809~"776",
GeoDist==0.983490397~"777",
GeoDist==0.033041368~"778",
GeoDist==0.032859519~"779",
GeoDist==1.542841674~"780",
GeoDist==1.379875719~"781",
GeoDist==1.662256965~"782",
GeoDist==0.876212619~"783",
GeoDist==1.872387628~"784",
GeoDist==0.366227266~"785",
GeoDist==1.682059273~"786",
GeoDist==1.739470357~"787",
GeoDist==2.617150285~"788",
GeoDist==2.095194744~"789",
GeoDist==1.488382729~"790",
GeoDist==2.054893319~"791",
GeoDist==2.367627211~"792",
GeoDist==2.294593005~"793",
GeoDist==2.294938058~"794",
GeoDist==2.219065174~"795",
GeoDist==2.379545273~"796",
GeoDist==2.560592482~"797",
GeoDist==2.936436151~"798",
GeoDist==4.002715866~"799",
GeoDist==1.396076394~"800",
GeoDist==1.421734305~"801",

GeoDist==2.383647525~"802",
GeoDist==1.804806358~"803",
GeoDist==1.141120469~"804",
GeoDist==1.705642256~"805",
GeoDist==2.058607016~"806",
GeoDist==2.099983723~"807",
GeoDist==2.100251691~"808",
GeoDist==1.866885537~"809",
GeoDist==2.039471806~"810",
GeoDist==2.208418476~"811",
GeoDist==2.667855~"812",
GeoDist==3.722889098~"813",
GeoDist==0.242808508~"814",
GeoDist==1.043110947~"815",
GeoDist==0.41318864~"816",
GeoDist==0.542901329~"817",
GeoDist==0.709098984~"818",
GeoDist==0.703884735~"819",
GeoDist==0.900979124~"820",
GeoDist==0.900926931~"821",
GeoDist==0.85963868~"822",
GeoDist==0.860914404~"823",
GeoDist==1.132264019~"824",
GeoDist==1.274140329~"825",
GeoDist==2.327284661~"826",
GeoDist==1.161954331~"827",
GeoDist==0.438605052~"828",
GeoDist==0.387752403~"829",
GeoDist==0.474594278~"830",
GeoDist==0.639113392~"831",
GeoDist==1.093349844~"832",
GeoDist==1.093205318~"833",
GeoDist==0.633662267~"834",
GeoDist==0.681375982~"835",
GeoDist==0.930589481~"836",
GeoDist==1.312359991~"837",
GeoDist==2.327191769~"838",
GeoDist==0.73969763~"839",
GeoDist==1.547088665~"840",
GeoDist==1.372419259~"841",
GeoDist==0.786413197~"842",
GeoDist==0.43171364~"843",
GeoDist==0.4311~"844",
GeoDist==1.412359605~"845",
GeoDist==1.194952137~"846",
GeoDist==1.448490185~"847",
GeoDist==0.461230244~"848",
GeoDist==1.47135787~"849",

GeoDist==0.825651761~"850",
GeoDist==0.667170202~"851",
GeoDist==0.32122307~"852",
GeoDist==0.771273893~"853",
GeoDist==0.770942101~"854",
GeoDist==0.75195859~"855",
GeoDist==0.623976559~"856",
GeoDist==0.916147737~"857",
GeoDist==0.879985176~"858",
GeoDist==1.918624047~"859",
GeoDist==0.566552574~"860",
GeoDist==0.9891341~"861",
GeoDist==1.443542543~"862",
GeoDist==1.443479799~"863",
GeoDist==0.732363793~"864",
GeoDist==0.90057068~"865",
GeoDist==1.073230145~"866",
GeoDist==1.692836584~"867",
GeoDist==2.686022348~"868",
GeoDist==0.624927301~"869",
GeoDist==1.435236236~"870",
GeoDist==1.434937961~"871",
GeoDist==0.17157501~"872",
GeoDist==0.362501016~"873",
GeoDist==0.507780728~"874",
GeoDist==1.363156176~"875",
GeoDist==2.247543761~"876",
GeoDist==0.96970312~"877",
GeoDist==0.969232002~"878",
GeoDist==0.632997674~"879",
GeoDist==0.410205242~"880",
GeoDist==0.680782818~"881",
GeoDist==0.741393199~"882",
GeoDist==1.696954495~"883",
GeoDist==0.000626099~"884",
GeoDist==1.522102842~"885",
GeoDist==1.363285257~"886",
GeoDist==1.647061657~"887",
GeoDist==0.888592956~"888",
GeoDist==1.893277137~"889",
GeoDist==1.521751013~"890",
GeoDist==1.362857905~"891",
GeoDist==1.646613434~"892",
GeoDist==0.887969838~"893",
GeoDist==1.892697456~"894",
GeoDist==0.273275338~"895",
GeoDist==0.341619209~"896",
GeoDist==1.345795509~"897",

GeoDist==2.168596096~"898",
GeoDist==0.292243112~"899",
GeoDist==1.083739652~"900",
GeoDist==1.898738004~"901",
GeoDist==1.271097195~"902",
GeoDist==1.951354833~"903",
GeoDist==1.072254026~"904",
GeoDist==1.086759623~"905",
GeoDist==3.520950423~"906",
GeoDist==4.396587752~"907",
GeoDist==4.558199079~"908",
GeoDist==1.319218707~"909",
GeoDist==1.023397519~"910",
GeoDist==3.080282467~"911",
GeoDist==3.958302943~"912",
GeoDist==4.093715932~"913",
GeoDist==1.170436789~"914",
GeoDist==0.170064221~"915",
GeoDist==2.277767813~"916",
GeoDist==3.150440087~"917",
GeoDist==3.313792929~"918",
GeoDist==0.088672995~"919",
GeoDist==2.434368044~"920",
GeoDist==0.933595394~"921",
GeoDist==3.310005072~"922",
GeoDist==3.471477646~"923",
GeoDist==0.64813897~"924",
GeoDist==1.73509856~"925",
GeoDist==0.233708011~"926",
GeoDist==0.696003697~"927",
GeoDist==0.789840145~"928",
GeoDist==1.766943888~"929",
GeoDist==0.657224324~"930",
GeoDist==1.528516145~"931",
GeoDist==0.105062039~"932",
GeoDist==0.757551859~"933",
GeoDist==0.23268047~"934",
GeoDist==3.174972352~"935",
GeoDist==0.883914907~"936",
GeoDist==1.037707391~"937",
GeoDist==2.931907709~"938",
GeoDist==0.724180018~"939",
GeoDist==2.201085236~"940",
GeoDist==3.107060444~"941",
GeoDist==2.765841073~"942",
GeoDist==0.707937871~"943",
GeoDist==2.042086688~"944",
GeoDist==0.917244952~"945",

```
    GeoDist==2.536165124~"946",
    GeoDist==1.894972504~"947",
    GeoDist==2.202313767~"948",
    GeoDist==4.013162186~"949",
    GeoDist==4.189191327~"950",
    GeoDist==1.103533713~"951",
    GeoDist==0.191559026~"952",
    GeoDist==3.782153433~"953",
    GeoDist==1.607348621~"954",
    GeoDist==3.077550147~"955",
    GeoDist==3.973620779~"956",
    GeoDist==3.58624667~"957",
    GeoDist==1.552677235~"958",
    GeoDist==2.877643939~"959",
    GeoDist==1.800882351~"960",
    GeoDist==3.412771144~"961",
    GeoDist==2.776530678~"962",
    GeoDist==3.077417658~"963",
    GeoDist==3.954886945~"964",
    GeoDist==1.75494032~"965",
    GeoDist==3.238374144~"966",
    GeoDist==4.140440634~"967",
    GeoDist==3.76559333~"968",
    GeoDist==1.723050114~"969",
    GeoDist==3.053339253~"970",
    GeoDist==1.953226622~"971",
    GeoDist==3.573539397~"972",
    GeoDist==2.91822551~"973",
    GeoDist==3.239211834~"974",
    GeoDist==0.820973015~"975",
    GeoDist==1.505908434~"976",
    GeoDist==0.029649368~"977",
    GeoDist==0.91720047~"978",
    GeoDist==0.870803968~"979",
    GeoDist==1.534523784~"980",
    GeoDist==0.511762328~"981",
    GeoDist==1.298693753~"982",
    GeoDist==0.336499166~"983",
    GeoDist==0.604778431~"984",)) -> df

#### end ####
#write.csv(df, file = "200612_testvalues.csv") -> df

simbygeo<-read.csv("200617_testvalues2.csv")

SIM_LME<-simbygeo$SIM
SOR_LME<-simbygeo$SOR
SNE_LME<-simbygeo$SNE
```

```
GeoDist_LME<-simbygeo$GeoDist
Pd_LME<-as.factor(simbygeo$Pd)
sitecontrast_LME<-as.factor(simbygeo$contrastID)

#######
#Avg
#######
#In order to remove nested data structure average data within comparisons
neg_LME<-subset(simbygeo, Pd=="0")
pos_LME<-subset(simbygeo, Pd=="1")

avgSIMvaluesneg<-aggregate(neg_LME$SIM~neg_LME$contrastID, neg_LME, mean)
#write.csv(avgSIMvaluesneg, file="200713_neg_avgSIM_values.csv")
avgSIMvaluespos<-aggregate(pos_LME$SIM~pos_LME$contrastID, pos_LME, mean)
#write.csv(avgSIMvaluespos, file="200713_pos_avgSIM_values.csv")
avgvaluegeoPos<-aggregate(pos_LME$GeoDist~pos_LME$contrastID, pos_LME, mean)
#write.csv(avgvaluegeoPos, file="200713_pos_avg_valuesGeo.csv")
avgvaluegeoneg<-aggregate(neg_LME$GeoDist~neg_LME$contrastID, neg_LME, mean)
#write.csv(avgvaluegeoneg, file="200713_neg_avg_valuesGeo.csv")
avgSORvaluesneg<-aggregate(neg_LME$SOR~neg_LME$contrastID, neg_LME, mean)
#write.csv(avgSORvaluesneg, file="200713_neg_avgSOR_values.csv")
avgSORvaluespos<-aggregate(pos_LME$SOR~pos_LME$contrastID, pos_LME, mean)
#write.csv(avgSORvaluespos, file="200713_pos_avgSOR_values.csv")
avgSNEvaluesneg<-aggregate(neg_LME$SNE~neg_LME$contrastID, neg_LME, mean)
#write.csv(avgSNEvaluesneg, file="200713_neg_avgSNE_values.csv")
avgSNEvaluespos<-aggregate(pos_LME$SNE~pos_LME$contrastID, pos_LME, mean)
#write.csv(avgSNEvaluespos, file="200713_pos_avgSNE_values.csv")

#avgSIMvaluespos<-read.csv("200713_pos_avgSIM_values.csv")
#avgvaluegeoPos<-read.csv("200713_pos_avg_valuesGeo.csv")
#avgSORvaluespos<-read.csv("200713_pos_avgSOR_values.csv")
#avgSNEvaluespos<-read.csv("200713_pos_avgSNE_values.csv")
#half<-merge(avgSIMvaluespos,avgvaluegeoPos, by="contrastID" )
#other<-merge(avgSORvaluespos,avgSNEvaluespos, by="contrastID" )
#pos_avgs<-merge(half, other, by="contrastID")
#write.csv(pos_avgs, file="200713_pos_avg.csv")

#avgSIMvaluesneg<-read.csv("200713_neg_avgSIM_values.csv")
#avgvaluegeoneg<-read.csv("200713_neg_avg_valuesGeo.csv")
#avgSORvaluesneg<-read.csv("200713_neg_avgSOR_values.csv")
#avgSNEvaluesneg<-read.csv("200713_neg_avgSNE_values.csv")
#halfneg<-merge(avgSIMvaluesneg,avgvaluegeoneg, by="contrastID" )
#otherneg<-merge(avgSORvaluesneg,avgSNEvaluesneg, by="contrastID" )
#neg_avgs<-merge(halfneg, otherneg, by="contrastID")
#write.csv(neg_avgs, file="200713_neg_avg.csv")
```

```
totalavgs<-read.csv("200713_totalAvg.csv")
AvgSIM<-totalavgs$AvgSIM
AvgSOR<-totalavgs$AvgSOR
AvgSNE<-totalavgs$AvgSNE
avgdist<-totalavgs$AvgGeoDist
avgpd<-as.factor(totalavgs$Pd)
avgsitecontrast<-as.factor(totalavgs$contrastID)

#Graphing
DDRPos<-subset(totalavgs, Pd=="1")
DDRNeg<-subset(totalavgs, Pd=="0")
PosAVGSOR<-DDRPos$AvgSOR
PosAVGSIM<-DDRPos$AvgSIM
PosAVGSNE<-DDRPos$AvgSNE
Posdist<-DDRPos$AvgGeoDist
NegAVGSOR<-DDRNeg$AvgSOR
NegAVGSIM<-DDRNeg$AvgSIM
NegAVGSNE<-DDRNeg$AvgSNE
Negdist<-DDRNeg$AvgGeoDist



mod4<-glm(AvgSIM~avgdist*avgpd,family=binomial)
summary(mod4)
Anova(mod4, type=2)
par(mfrow=c(1,2))
plot(residuals(mod4)~fitted(mod4), main="residuals vs fitted")
qqnorm(residuals(mod4))
qqline(residuals(mod4))
par(mfrow=c(1,1))

B2 <- glm(PosAVGSOR~Posdist, family=binomial, data=DDRPos)
summary(B2)
B2_1<-glm(NegAVGSOR~Negdist, family=binomial, data=DDRNeg)
summary(B2_1)
plot(PosAVGSOR~Posdist, data=DDRPos, type='n', bty="n", xlim=c(0,5), ylim=c(0.8,1),xlab="(log)
Geographic Distance", ylab="Average SOR")
points(Posdist, PosAVGSOR, pch=19, cex=0.75, col="black", bg="black")
points(Negdist, NegAVGSOR, pch=19, cex=0.75, col="grey", bg="grey")
axis(side=1, lwd=2)
axis(side=2, lwd=2)
title("Total Beta Diversity", adj=0, line=0.25, cex.main=1.5)
title("B", adj=1, line=-17, cex.main=2)
pred <-seq (0,5,0.01)
B2.pred <- predict(B2, list(Posdist=pred), type="response", data=DDRPos)
lines(pred, B2.pred, lty=2, col="blue", lwd=5)
B2_1.pred<-predict(B2_1, list(Negdist=pred), type="response", data=DDRNeg)
lines(pred, B2_1.pred, lty=1, col="blue", lwd=5)
```

```
mod4_1<-glm(AvgSOR~avgdist*avgpd,family=binomial)# Warning of non-interger #successess in a binomial glm is ok, just telling us that we have set it to binomial
summary(mod4_1)
Anova(mod4_1, type=2)
par(mfrow=c(1,2))
plot(residuals(mod4_1)~fitted(mod4_1), main="residuals vs fitted")
qqnorm(residuals(mod4_1))
qqline(residuals(mod4_1))
par(mfrow=c(1,1))

B3 <- glm(PosAVGSIM~Posdist, family=binomial, data=DDRPos)
summary(B3)
B3_1<-glm(NegAVGSIM~Negdist, family=binomial, data=DDRNeg)
summary(B3_1)
plot(PosAVGSIM~Posdist, data=DDRPos, type='n', bty="n", xlim=c(0,5), xlab="(log) Geographic Distance", ylab="Average SIM")
points(Posdist, PosAVGSIM, pch=19, cex=0.75, col="black", bg="black")
points(Negdist, NegAVGSIM, pch=19, cex=0.75, col="grey", bg="grey")
axis(side=1, lwd=2)
axis(side=2, lwd=2)
title("Turnover", adj=0, line=0.25, cex.main=1.5)
title("C", adj=1, line=-17, cex.main=2)
pred <-seq (0,5,0.01)
B3.pred <- predict(B3, list(Posdist=pred), type="response", data=DDRPos)
lines(pred, B3.pred, lty=2, col="blue", lwd=5)
B3_1.pred<-predict(B3_1, list(Negdist=pred), type="response", data=DDRNeg)
lines(pred, B3_1.pred, lty=1, col="blue", lwd=5)

mod4_2<-glm(AvgSNE~avgdist*avgpd,family=binomial)# Warning of non-interger #successess in a binomial glm is ok, just telling us that we have set it to binomial
summary(mod4_2)
Anova(mod4_2, type=2)
par(mfrow=c(1,2))
plot(residuals(mod4_2)~fitted(mod4_2), main="residuals vs fitted")
qqnorm(residuals(mod4_2))
qqline(residuals(mod4_2))
par(mfrow=c(1,1))

B4 <- glm(PosAVGSNE~Posdist, family=binomial, data=DDRPos)
summary(B3)
B4_1<-glm(NegAVGSNE~Negdist, family=binomial, data=DDRNeg)
summary(B4_1)
plot(PosAVGSNE~Posdist, data=DDRPos, type='n', bty="n", xlim=c(0,5), ylim=c(0,0.15),xlab="(log) Geographic Distance", ylab="Average SNE")
points(Posdist, PosAVGSNE, pch=16, cex=0.75, col="black", bg="black")
points(Negdist, NegAVGSNE, pch=19, cex=0.75, col="grey", bg="grey")
axis(side=1, lwd=2)
```

```
axis(side=2, lwd=2)
title("Nestedness", adj=0, line=0.25, cex.main=1.5)
title("D", adj=1, line=-17, cex.main=2)
pred <-seq (0,5,0.01)
B4.pred <- predict(B4, list(Posdist=pred), type="response", data=DDRPos)
lines(pred, B4.pred, lty=2, col="blue", lwd=5)
B4_1.pred<-predict(B4_1, list(Negdist=pred), type="response", data=DDRNeg)
lines(pred, B4_1.pred, lty=1, col="blue", lwd=5)
legend(3.5, 0.16, legend=c("Negative", "Positive"),
    col=c("blue", "blue"), lty=1:2, cex=0.8,box.lty=0)
####end####
############
#Copies
############
####Copies####
copies<-positive$copies
logcopies<-log(copies+1)
copiesdist<-dist(copies, method="euclidean")
logcopiesdist<-dist(logcopies)

####Copies Dataframe code ####
#posdfSIM<-as.matrix(SIM_pos)
#posdfSIM[upper.tri(posdfSIM)] <- NA
#posdfSOR<-as.matrix(SOR_pos)
#posdfSOR[upper.tri(posdfSOR)] <- NA
#posdfSNE<-as.matrix(SNE_pos)
#posdfSNE[upper.tri(posdfSNE)] <- NA
#logcopiesmat<-as.matrix(logcopiesdist)
#logcopiesmat[upper.tri(logcopiesmat)] <- NA
#rownames(logcopiesmat)<-positive$Group
#colnames(logcopiesmat)<-positive$Group
#SIM_posmelt<-melt(posdfSIM)
#SOR_posmelt<-melt(posdfSOR)
#SNE_posmelt<-melt(posdfSNE)
#posdistdf<-as.matrix(posdist)
#rownames(posdistdf)<-positive$Group
#colnames(posdistdf)<-positive$Group
#posdistdf[upper.tri(posdistdf)] <- NA
#posdistmelt<-melt(posdistdf)
#logcopiesmelt<-melt(logcopiesmat)
#write.csv(posdistmelt, file="2006_20posdistmelt.csv")
#write.csv(SIM_posmelt, file="2006_20_SIM_posmelt.csv")
#write.csv(SOR_posmelt, file="2006_20_SOR_posmelt.csv")
#write.csv(SNE_posmelt, file="2006_20_SNE_posmelt.csv")
#write.csv(logcopiesmelt, file="2006_20_copies_posmelt.csv")

#GeoPos<-read.csv("2006_20posdistmelt.csv")
#SIMPos<-read.csv("2006_20_SIM_posmelt.csv")
```

```
#SNEPos<-read.csv("2006_20_SNE_posmelt.csv")
#SORPos<-read.csv("2006_20_SOR_posmelt.csv")
#copiesPos<-read.csv("2006_20_copies_posmelt.csv")
#Total<-read.csv("200612_totalgeodist.csv")

#posSIM_database1<-merge(GeoPos,SIMPos, by="g")
#posSIM_database2<-merge(SORPos,SNEPos, by="g")
#posSIM_database3<-merge(copiesPos,posSIM_database2, by="g")
#posSIM_database4<-merge(posSIM_database3,posSIM_database1, by="g")

#write.csv(posSIM_database4, file="200621_Pos_database.csv")

#df <- read.csv("200621_Pos_database.csv", header = TRUE, row.names = NULL)

#dist.unique<-unique(df$GeoDist)
#write.csv(dist.unique, file = "200621_testunique.csv")
#### Contrasts ####
df %>%
  mutate(contrastID = case_when( GeoDist==0~"1",
                              GeoDist==1.5097858~"2",
                              GeoDist==0.402880439~"3",
                              GeoDist==1.791783621~"4",
                              GeoDist==0.996404482~"5",
                              GeoDist==0.581531788~"6",
                              GeoDist==2.066129731~"7",
                              GeoDist==2.054876971~"8",
                              GeoDist==0.967784297~"9",
                              GeoDist==2.182943807~"10",
                              GeoDist==0.944126772~"11",
                              GeoDist==0.420564816~"12",
                              GeoDist==0.291333197~"13",
                              GeoDist==1.227921213~"14",
                              GeoDist==0.013250151~"15",
                              GeoDist==0.31860752~"16",
                              GeoDist==0.863139413~"17",
                              GeoDist==0.390690891~"18",
                              GeoDist==0.473648934~"19",
                              GeoDist==0.330083722~"20",
                              GeoDist==1.74871363~"21",
                              GeoDist==1.626460587~"22",
                              GeoDist==1.757356682~"23",
                              GeoDist==0.263555892~"24",
                              GeoDist==0.611354526~"25",
                              GeoDist==2.200544153~"26",
                              GeoDist==1.067261149~"27",
                              GeoDist==0.871493558~"28",
                              GeoDist==1.722485878~"29",
                              GeoDist==0.887555445~"30",
```

GeoDist==1.389153923~"31",
GeoDist==1.118161381~"32",
GeoDist==2.416751496~"33",
GeoDist==1.552561163~"34",
GeoDist==2.022047163~"35",
GeoDist==0.374081583~"36",
GeoDist==1.457536879~"37",
GeoDist==0.044369653~"38",
GeoDist==1.294849736~"39",
GeoDist==0.399847805~"40",
GeoDist==1.831071603~"41",
GeoDist==0.9145334~"42",
GeoDist==2.108384487~"43",
GeoDist==2.072612284~"44",
GeoDist==0.666310432~"45",
GeoDist==1.33509152~"46",
GeoDist==0.519514851~"47",
GeoDist==0.886296062~"48",
GeoDist==0.911839658~"49",
GeoDist==2.488722476~"50",
GeoDist==2.232064809~"51",
GeoDist==1.701140174~"52",
GeoDist==2.387310202~"53",
GeoDist==0.828551446~"54",
GeoDist==0.925077407~"55",
GeoDist==0.809592161~"56",
GeoDist==2.20463378~"57",
GeoDist==1.600399485~"58",
GeoDist==1.144756753~"59",
GeoDist==1.160005534~"60",
GeoDist==1.42894965~"61",
GeoDist==0.794736792~"62",
GeoDist==1.43226328~"63",
GeoDist==0.035656295~"64",
GeoDist==0.641326688~"65",
GeoDist==1.565731513~"66",
GeoDist==0.943187679~"67",
GeoDist==2.078928005~"68",
GeoDist==1.409536703~"69",
GeoDist==1.421838839~"70",
GeoDist==0.996258452~"71",
GeoDist==0.424894247~"72",
GeoDist==0.51555363~"73",
GeoDist==1.256658636~"74",
GeoDist==2.612321942~"75",
GeoDist==0.613055534~"76",
GeoDist==0.254525895~"77",
GeoDist==0.838165294~"78",

GeoDist==1.749272882~"79",
GeoDist==2.286988291~"80",
GeoDist==0.945952473~"81",
GeoDist==2.850846701~"82",
GeoDist==2.095586338~"83",
GeoDist==0.048825736~"84",
GeoDist==0.709401733~"85",
GeoDist==1.110908381~"86",
GeoDist==0.964223147~"87",
GeoDist==0.385987091~"88",
GeoDist==1.204750267~"89",
GeoDist==0.196917975~"90",
GeoDist==1.892697456~"91",
GeoDist==1.661557752~"92",
GeoDist==1.700349417~"93",
GeoDist==1.398601493~"94",
GeoDist==1.903913356~"95",
GeoDist==1.002261517~"96",
GeoDist==0.74904653~"97",
GeoDist==0.786130753~"98",
GeoDist==1.860707935~"99",
GeoDist==1.883369444~"100",
GeoDist==1.449610188~"101",
GeoDist==0.560580317~"102",
GeoDist==2.016323208~"103",
GeoDist==0.029456655~"104",
GeoDist==1.5569359~"105",
GeoDist==1.089800022~"106",
GeoDist==0.857675891~"107",
GeoDist==2.051503555~"108",
GeoDist==0.3606545~"109",
GeoDist==0.056905081~"110",
GeoDist==0.608595845~"111",
GeoDist==0.679897006~"112",
GeoDist==0.83172713~"113",
GeoDist==1.828934569~"114",
GeoDist==1.728862072~"115",
GeoDist==1.075720946~"116",
GeoDist==0.538244543~"117",
GeoDist==1.271903372~"118",
GeoDist==1.92236128~"119",
GeoDist==0.786406682~"120",
GeoDist==1.083739652~"121",
GeoDist==0.285642571~"122",
GeoDist==1.794530505~"123",
GeoDist==0.620589047~"124",
GeoDist==0.534923562~"125",
GeoDist==2.721946553~"126",

GeoDist==1.173243727~"127",
GeoDist==1.706852972~"128",
GeoDist==1.741043977~"129",
GeoDist==1.324444343~"130",
GeoDist==1.374496061~"131",
GeoDist==0.505765528~"132",
GeoDist==0.718244719~"133",
GeoDist==1.948514933~"134",
GeoDist==1.326056323~"135",
GeoDist==0.507488551~"136",
GeoDist==1.961552143~"137",
GeoDist==1.092186626~"138",
GeoDist==2.438924313~"139",
GeoDist==1.167055105~"140",
GeoDist==1.407018832~"141",
GeoDist==0.961434495~"142",
GeoDist==1.310379212~"143",
GeoDist==2.517913643~"144",
GeoDist==1.467062009~"145",
GeoDist==2.111945154~"146",
GeoDist==3.215234048~"147",
GeoDist==1.073963045~"148",
GeoDist==0.030274755~"149",
GeoDist==2.189426127~"150",
GeoDist==1.461894232~"151",
GeoDist==0.647344754~"152",
GeoDist==0.396107275~"153",
GeoDist==1.569948301~"154",
GeoDist==2.712075655~"155",
GeoDist==0.476557953~"156",
GeoDist==0.887969838~"157",
GeoDist==2.245251951~"158",
GeoDist==0.561137447~"159",
GeoDist==0.957560105~"160",
GeoDist==0.090643716~"161",
GeoDist==1.780711337~"162",
GeoDist==0.38151491~"163",
GeoDist==1.425243087~"164",
GeoDist==1.441690879~"165",
GeoDist==2.326106897~"166",
GeoDist==1.363917943~"167",
GeoDist==0.971278002~"168",
GeoDist==1.74423104~"169",
GeoDist==2.204121982~"170",
GeoDist==2.537570467~"171",
GeoDist==0.712701825~"172",
GeoDist==1.858793307~"173",
GeoDist==1.313440762~"174",

GeoDist==3.574387635~"175",
GeoDist==1.338823605~"176",
GeoDist==1.978638224~"177",
GeoDist==0.966164021~"178",
GeoDist==0.27579855~"179",
GeoDist==1.707189656~"180",
GeoDist==1.701610184~"181",
GeoDist==2.470149569~"182",
GeoDist==1.754234876~"183",
GeoDist==1.319866302~"184",
GeoDist==1.329273545~"185",
GeoDist==1.030086378~"186",
GeoDist==1.507189912~"187",
GeoDist==0.308193063~"188",
GeoDist==1.284026106~"189",
GeoDist==2.101053879~"190",
GeoDist==1.149621026~"191",
GeoDist==1.362857905~"192",
GeoDist==0.076814033~"193",
GeoDist==0.542901329~"194",
GeoDist==0.551252933~"195",
GeoDist==0.290105293~"196",
GeoDist==1.047633173~"197",
GeoDist==0.467055337~"198",
GeoDist==2.640902005~"199",
GeoDist==2.141400392~"200",
GeoDist==0.387129765~"201",
GeoDist==0.960605794~"202",
GeoDist==0.801985279~"203",
GeoDist==1.978488037~"204",
GeoDist==1.537170109~"205",
GeoDist==0.697909901~"206",
GeoDist==1.757174211~"207",
GeoDist==0.368954118~"208",
GeoDist==1.236631568~"209",
GeoDist==1.324273485~"210",
GeoDist==0.324853283~"211",
GeoDist==2.020748402~"212",
GeoDist==1.379417315~"213",
GeoDist==1.339083638~"214",
GeoDist==0.730815489~"215",
GeoDist==1.335529661~"216",
GeoDist==1.560822104~"217",
GeoDist==0.967952012~"218",
GeoDist==0.494133804~"219",
GeoDist==1.974802208~"220",
GeoDist==1.745163769~"221",
GeoDist==1.465905806~"222",

GeoDist==0.580717731~"223",
GeoDist==1.62826623~"224",
GeoDist==0.728677246~"225",
GeoDist==0.564785632~"226",
GeoDist==1.522216348~"227",
GeoDist==0.327775936~"228",
GeoDist==0.599633475~"229",
GeoDist==1.59397345~"230",
GeoDist==2.274170836~"231",
GeoDist==1.038667487~"232",
GeoDist==1.875125575~"233",
GeoDist==1.16778086~"234",
GeoDist==1.216317019~"235",
GeoDist==3.087635405~"236",
GeoDist==0.341663772~"237",
GeoDist==0.958348196~"238",
GeoDist==0.541670302~"239",
GeoDist==0.478570777~"240",
GeoDist==0.832435325~"241",
GeoDist==1.914894603~"242",
GeoDist==0.684649038~"243",
GeoDist==1.072254026~"244",
GeoDist==2.238492708~"245",
GeoDist==1.324493267~"246",
GeoDist==1.10431629~"247",
GeoDist==0.543398537~"248",
GeoDist==0.952102163~"249",
GeoDist==1.094824359~"250",
GeoDist==0.849306935~"251",
GeoDist==1.82960507~"252",
GeoDist==1.066452383~"253",
GeoDist==1.755662278~"254",
GeoDist==0.179027712~"255",
GeoDist==2.213039177~"256",
GeoDist==1.355499965~"257",
GeoDist==3.545491992~"258",
GeoDist==1.241800742~"259",
GeoDist==1.951354833~"260",
GeoDist==0.784450002~"261",
GeoDist==1.64006398~"262",
GeoDist==2.694086~"263",
GeoDist==2.475698895~"264",
GeoDist==1.898738004~"265",
GeoDist==1.681599352~"266",
GeoDist==2.242221867~"267",
GeoDist==0.79841956~"268",
GeoDist==2.005506117~"269",
GeoDist==2.517460799~"270",

GeoDist==0.772397676~"271",
GeoDist==1.908492334~"272",
GeoDist==2.104206531~"273",
GeoDist==1.521347964~"274",
GeoDist==0.340115078~"275",
GeoDist==2.1809561~"276",
GeoDist==0.877550124~"277",
GeoDist==3.270200202~"278",
GeoDist==0.580697864~"279",
GeoDist==1.967177528~"280",
GeoDist==1.203405273~"281",
GeoDist==0.879985176~"282",
GeoDist==1.449652682~"283",
GeoDist==2.923054939~"284",
GeoDist==1.561515228~"285",
GeoDist==2.277223305~"286",
GeoDist==0.208413073~"287",
GeoDist==2.596768695~"288",
GeoDist==1.354312224~"289",
GeoDist==1.834944586~"290",
GeoDist==0.914313194~"291",
GeoDist==2.280010983~"292",
GeoDist==1.378322912~"293",
GeoDist==1.028692948~"294",
GeoDist==1.135855916~"295",
GeoDist==0.772005426~"296",
GeoDist==1.285091882~"297",
GeoDist==0.510064177~"298",
GeoDist==2.817564725~"299",
GeoDist==1.922102139~"300",
GeoDist==0.802054431~"301",
GeoDist==0.534038062~"302",
GeoDist==1.010503693~"303",
GeoDist==1.121868089~"304",
GeoDist==2.063726219~"305",
GeoDist==0.666092158~"306",
GeoDist==2.357506619~"307",
GeoDist==0.89807746~"308",
GeoDist==0.598288301~"309",
GeoDist==0.766581449~"310",
GeoDist==2.012695867~"311",
GeoDist==1.031291581~"312",
GeoDist==1.887054591~"313",
GeoDist==0.083643073~"314",
GeoDist==2.230117626~"315",
GeoDist==2.355523942~"316",
GeoDist==1.288800999~"317",
GeoDist==3.144499556~"318",

GeoDist==1.692836584~"319",
GeoDist==0.993486355~"320",
GeoDist==1.927741539~"321",
GeoDist==1.6706015~"322",
GeoDist==1.942257592~"323",
GeoDist==0.6811341~"324",
GeoDist==1.607615156~"325",
GeoDist==0.569515965~"326",
GeoDist==1.656981983~"327",
GeoDist==0.738676899~"328",
GeoDist==1.110782182~"329",
GeoDist==0.451015762~"330",
GeoDist==1.216987717~"331",
GeoDist==1.143039605~"332",
GeoDist==0.132370109~"333",
GeoDist==0.684182945~"334",
GeoDist==2.421638542~"335",
GeoDist==0.427187547~"336",
GeoDist==2.203083203~"337",
GeoDist==0.964973662~"338",
GeoDist==2.086893339~"339",
GeoDist==0.905336995~"340",
GeoDist==1.394486318~"341",
GeoDist==1.162886638~"342",
GeoDist==1.871016513~"343",
GeoDist==0.449898611~"344",
GeoDist==1.484650426~"345",
GeoDist==1.410984598~"346",
GeoDist==1.323716454~"347",
GeoDist==2.058607016~"348",
GeoDist==1.954898842~"349",
GeoDist==1.422396584~"350",
GeoDist==1.20839196~"351",
GeoDist==0.741393199~"352",
GeoDist==0.776060382~"353",
GeoDist==2.069682125~"354",
GeoDist==0.720608595~"355",
GeoDist==0.316869688~"356",
GeoDist==2.026501961~"357",
GeoDist==2.320075383~"358",
GeoDist==0.750152072~"359",
GeoDist==1.616218665~"360",
GeoDist==0.883069683~"361",
GeoDist==1.404488882~"362",
GeoDist==0.80474225~"363",
GeoDist==1.124914596~"364",
GeoDist==1.233017438~"365",
GeoDist==0.961128184~"366",

GeoDist==1.390630115~"367",
GeoDist==4.002715866~"368",
GeoDist==0.976872935~"369",
GeoDist==2.359912164~"370",
GeoDist==0.601065366~"371",
GeoDist==1.411903641~"372",
GeoDist==1.951355315~"373",
GeoDist==2.594822404~"374",
GeoDist==0.850934089~"375",
GeoDist==0.700678169~"376",
GeoDist==0.047350803~"377",
GeoDist==0.396729357~"378",
GeoDist==0.416778849~"379",
GeoDist==1.236838722~"380",
GeoDist==2.358408059~"381",
GeoDist==0.762619727~"382",
GeoDist==0.767652536~"383",
GeoDist==2.168596096~"384",
GeoDist==0.90961362~"385",
GeoDist==1.177066939~"386",
GeoDist==4.474073447~"387",
GeoDist==0.888592956~"388",
GeoDist==1.208144917~"389",
GeoDist==0.923624561~"390",
GeoDist==0.877308772~"391",
GeoDist==0.891621576~"392",
GeoDist==2.420946194~"393",
GeoDist==2.076238959~"394",
GeoDist==0.476932273~"395",
GeoDist==0.56338169~"396",
GeoDist==0.404574939~"397",
GeoDist==1.095467305~"398",
GeoDist==2.971650227~"399",
GeoDist==0.339631322~"400",
GeoDist==2.264510024~"401",
GeoDist==1.610330888~"402",
GeoDist==0.931653418~"403",
GeoDist==1.274140329~"404",
GeoDist==1.764513579~"405",
GeoDist==0.459780738~"406",
GeoDist==1.556111351~"407",
GeoDist==1.592759535~"408",
GeoDist==2.686022348~"409",
GeoDist==1.213774786~"410",
GeoDist==0.991186946~"411",
GeoDist==0.732303045~"412",
GeoDist==0.940952619~"413",
GeoDist==2.319575727~"414",

GeoDist==1.425238011~"415",
GeoDist==1.918624047~"416",
GeoDist==0.632997674~"417",
GeoDist==1.620730737~"418",
GeoDist==2.696655581~"419",
GeoDist==0.548628219~"420",
GeoDist==1.409057727~"421",
GeoDist==1.443479799~"422",
GeoDist==2.405855334~"423",
GeoDist==0.92482297~"424",
GeoDist==0.892175891~"425",
GeoDist==0.231793249~"426",
GeoDist==0.43171364~"427",
GeoDist==2.431202204~"428",
GeoDist==2.796938409~"429",
GeoDist==0.628555469~"430",
GeoDist==1.514895717~"431",
GeoDist==1.229152336~"432",
GeoDist==1.653082016~"433",
GeoDist==1.854591491~"434",
GeoDist==0.275733583~"435",
GeoDist==0.54896562~"436",
GeoDist==2.3284851~"437",
GeoDist==2.834788898~"438",
GeoDist==0.916147737~"439",
GeoDist==1.121676207~"440",
GeoDist==1.537375075~"441",
GeoDist==0.461230244~"442",
GeoDist==1.120508855~"443",
GeoDist==1.473976448~"444",
GeoDist==1.834250206~"445",
GeoDist==2.320914402~"446",
GeoDist==1.951094644~"447",
GeoDist==2.026864604~"448",
GeoDist==2.613450267~"449",
GeoDist==0.868677022~"450",
GeoDist==1.014911736~"451",
GeoDist==1.014923773~"452",
GeoDist==1.603826619~"453",
GeoDist==0.444136563~"454",
GeoDist==3.722889098~"455",
GeoDist==0.96970312~"456",
GeoDist==1.153182792~"457",
GeoDist==1.062264256~"458",
GeoDist==0.541554476~"459",
GeoDist==2.932342473~"460",
GeoDist==1.218237852~"461",
GeoDist==1.678410688~"462",

GeoDist==0.29091546~"463",
GeoDist==0.623306924~"464",
GeoDist==0.484911034~"465",
GeoDist==0.9891341~"466",
GeoDist==1.334174213~"467",
GeoDist==0.779693468~"468",
GeoDist==3.401897272~"469",
GeoDist==2.936304999~"470",
GeoDist==0.144399979~"471",
GeoDist==0.846780341~"472",
GeoDist==0.822499546~"473",
GeoDist==2.56781213~"474",
GeoDist==0.756987843~"475",
GeoDist==1.029505762~"476",
GeoDist==1.989332733~"477",
GeoDist==0.743657836~"478",
GeoDist==0.186563122~"479",
GeoDist==0.984849232~"480",
GeoDist==3.752197537~"481",
GeoDist==1.695681726~"482",
GeoDist==0.969232002~"483",
GeoDist==0.862549846~"484",
GeoDist==1.271097195~"485",
GeoDist==0.538478673~"486",
GeoDist==1.21444534~"487",
GeoDist==0.346626222~"488",
GeoDist==1.20618611~"489",
GeoDist==0.180606302~"490",
GeoDist==0.387749893~"491",
GeoDist==0.401233338~"492",
GeoDist==0.931328583~"493",
GeoDist==1.528854222~"494",
GeoDist==0.79036764~"495",
GeoDist==1.37987817~"496",
GeoDist==2.208418476~"497",
GeoDist==1.234437778~"498",
GeoDist==2.001975306~"499",
GeoDist==1.413548863~"500",
GeoDist==1.335830289~"501",
GeoDist==2.057954517~"502",
GeoDist==0.17157501~"503",
GeoDist==1.864984694~"504",
GeoDist==0.748193035~"505",
GeoDist==2.327284661~"506",
GeoDist==1.312359991~"507",
GeoDist==1.570433067~"508",
GeoDist==0.69760636~"509",
GeoDist==2.533219087~"510",

GeoDist==0.385004796~"511",
GeoDist==0.609270374~"512",
GeoDist==0.740304456~"513",
GeoDist==1.14069024~"514",
GeoDist==0.831887407~"515",
GeoDist==1.872387628~"516",
GeoDist==0.424326415~"517",
GeoDist==0.143379302~"518",
GeoDist==0.412364965~"519",
GeoDist==0.876551794~"520",
GeoDist==2.075592877~"521",
GeoDist==2.667855~"522",
GeoDist==1.354467982~"523",
GeoDist==1.33608678~"524",
GeoDist==1.200017092~"525",
GeoDist==1.363603097~"526",
GeoDist==0.000626099~"527",
GeoDist==0.983490397~"528",
GeoDist==2.881759218~"529",
GeoDist==1.148609515~"530",
GeoDist==1.50776415~"531",
GeoDist==0.424951379~"532",
GeoDist==1.896708445~"533",
GeoDist==0.627431088~"534",
GeoDist==1.563729516~"535",
GeoDist==0.900926931~"536",
GeoDist==2.327191769~"537",
GeoDist==0.623976559~"538",
GeoDist==0.549868749~"539",
GeoDist==2.229122204~"540",
GeoDist==0.032859519~"541",
GeoDist==1.547088665~"542",
GeoDist==1.789869703~"543",
GeoDist==2.125229511~"544",
GeoDist==1.337821359~"545",
GeoDist==1.025074427~"546",
GeoDist==0.939399681~"547",
GeoDist==0.335239041~"548",
GeoDist==1.229189691~"549",
GeoDist==1.202767834~"550",
GeoDist==1.068145305~"551",
GeoDist==2.681025627~"552",
GeoDist==3.046361466~"553",
GeoDist==0.811115361~"554",
GeoDist==1.298772735~"555",
GeoDist==0.876946435~"556",
GeoDist==0.507720679~"557",
GeoDist==0.73161459~"558",

GeoDist==0.083028479~"559",
GeoDist==1.25259091~"560",
GeoDist==2.506460142~"561",
GeoDist==1.20712953~"562",
GeoDist==2.23822047~"563",
GeoDist==2.246735954~"564",
GeoDist==2.23253424~"565",
GeoDist==1.459076424~"566",
GeoDist==1.161954331~"567",
GeoDist==0.899073744~"568",
GeoDist==1.804806358~"569",
GeoDist==3.764184863~"570",
GeoDist==0.391818077~"571",
GeoDist==1.647061657~"572",
GeoDist==0.755686683~"573",
GeoDist==0.293462828~"574",
GeoDist==2.16949064~"575",
GeoDist==0.620369135~"576",
GeoDist==0.75195859~"577",
GeoDist==1.696954495~"578",
GeoDist==0.307332707~"579",
GeoDist==2.15642098~"580",
GeoDist==1.665888573~"581",
GeoDist==1.194952137~"582",
GeoDist==1.456857493~"583",
GeoDist==1.279934336~"584",
GeoDist==1.671281825~"585",
GeoDist==0.187943434~"586",
GeoDist==0.387752403~"587",
GeoDist==0.766645657~"588",
GeoDist==0.51999389~"589",
GeoDist==1.211960316~"590",
GeoDist==1.501549324~"591",
GeoDist==0.474594278~"592",
GeoDist==0.374567603~"593",
GeoDist==1.363285257~"594",
GeoDist==0.41318864~"595",
GeoDist==1.189772992~"596",
GeoDist==2.202653281~"597",
GeoDist==1.045719284~"598",
GeoDist==0.514740437~"599",
GeoDist==1.247173828~"600",
GeoDist==2.132714173~"601",
GeoDist==0.060437154~"602",
GeoDist==1.132264019~"603",
GeoDist==1.043110947~"604",
GeoDist==2.785176254~"605",
GeoDist==1.56589945~"606",

GeoDist==2.032020398~"607",
GeoDist==2.157649944~"608",
GeoDist==2.410822468~"609",
GeoDist==1.47915676~"610",
GeoDist==1.403380562~"611",
GeoDist==0.703884735~"612",
GeoDist==0.85963868~"613",
GeoDist==1.893277137~"614",
GeoDist==0.85368171~"615",
GeoDist==1.521751013~"616",
GeoDist==0.869447387~"617",
GeoDist==0.742359849~"618",
GeoDist==1.542830837~"619",
GeoDist==0.581307141~"620",
GeoDist==1.983880462~"621",
GeoDist==1.40745183~"622",
GeoDist==0.835049024~"623",
GeoDist==0.866433455~"624",
GeoDist==1.018877476~"625",
GeoDist==2.05183139~"626",
GeoDist==1.480483854~"627",
GeoDist==0.667170202~"628",
GeoDist==0.68198234~"629",
GeoDist==0.410205242~"630",
GeoDist==0.910597514~"631",
GeoDist==0.503557709~"632",
GeoDist==2.124956969~"633",
GeoDist==1.586060483~"634",
GeoDist==2.088611987~"635",
GeoDist==2.247526549~"636",
GeoDist==3.307348869~"637",
GeoDist==0.688620099~"638",
GeoDist==0.560010745~"639",
GeoDist==3.13260012~"640",
GeoDist==0.895763989~"641",
GeoDist==0.34657368~"642",
GeoDist==2.039471806~"643",
GeoDist==0.133642958~"644",
GeoDist==0.744653478~"645",
GeoDist==0.274564945~"646",
GeoDist==1.251644424~"647",
GeoDist==0.900979124~"648",
GeoDist==0.918648007~"649",
GeoDist==2.054893319~"650",
GeoDist==2.247543761~"651",
GeoDist==0.812270325~"652",
GeoDist==1.094782485~"653",
GeoDist==1.093205318~"654",

GeoDist==0.373960895~"655",
GeoDist==0.151149783~"656",
GeoDist==1.722290869~"657",
GeoDist==1.971861251~"658",
GeoDist==0.919394814~"659",
GeoDist==0.592241945~"660",
GeoDist==1.135025435~"661",
GeoDist==2.259534269~"662",
GeoDist==0.712181496~"663",
GeoDist==1.776716236~"664",
GeoDist==2.326772881~"665",
GeoDist==1.822934487~"666",
GeoDist==1.898120557~"667",
GeoDist==0.770942101~"668",
GeoDist==0.292243112~"669",
GeoDist==1.443542543~"670",
GeoDist==1.171226044~"671",
GeoDist==0.762496136~"672",
GeoDist==1.472294581~"673",
GeoDist==3.57170676~"674",
GeoDist==2.216942632~"675",
GeoDist==0.786413197~"676",
GeoDist==0.383687436~"677",
GeoDist==0.608869435~"678",
GeoDist==0.613688084~"679",
GeoDist==0.980582241~"680",
GeoDist==1.358378774~"681",
GeoDist==0.32122307~"682",
GeoDist==1.438282294~"683",
GeoDist==2.292547254~"684",
GeoDist==0.111195641~"685",
GeoDist==3.043910811~"686",
GeoDist==1.543103541~"687",
GeoDist==0.946240645~"688",
GeoDist==1.134885723~"689",
GeoDist==1.745365968~"690",
GeoDist==1.448490185~"691",
GeoDist==0.8425968~"692",
GeoDist==0.366227266~"693",
GeoDist==1.180478642~"694",
GeoDist==0.624927301~"695",
GeoDist==0.505322856~"696",
GeoDist==0.981027393~"697",
GeoDist==1.652600224~"698",
GeoDist==0.751950205~"699",
GeoDist==0.876212619~"700",
GeoDist==0.771273893~"701",
GeoDist==1.379875719~"702",

GeoDist==2.678154814~"703",
GeoDist==1.110895216~"704",
GeoDist==0.945186226~"705",
GeoDist==1.47135787~"706",
GeoDist==1.045055622~"707",
GeoDist==1.866885537~"708",
GeoDist==1.34251454~"709",
GeoDist==1.640458374~"710",
GeoDist==0.90057068~"711",
GeoDist==0.956798081~"712",
GeoDist==0.681375982~"713",
GeoDist==0.377409325~"714",
GeoDist==2.680594995~"715",
GeoDist==1.141120469~"716",
GeoDist==2.066848808~"717",
GeoDist==2.099983723~"718",
GeoDist==0.656120478~"719",
GeoDist==1.438051753~"720",
GeoDist==1.022670193~"721",
GeoDist==0.4311~"722",
GeoDist==0.679656085~"723",
GeoDist==1.56050811~"724",
GeoDist==0.473699992~"725",
GeoDist==0.497239441~"726",
GeoDist==2.383647525~"727",
GeoDist==0.680782818~"728",
GeoDist==0.341619209~"729",
GeoDist==2.392134075~"730",
GeoDist==0.898667097~"731",
GeoDist==1.488382729~"732",
GeoDist==0.463631356~"733",
GeoDist==3.240492136~"734",
GeoDist==0.684930433~"735",
GeoDist==0.853700246~"736",
GeoDist==0.657858174~"737",
GeoDist==0.730006118~"738",
GeoDist==2.787954104~"739",
GeoDist==2.886459929~"740",
GeoDist==1.132309716~"741",
GeoDist==0.855190218~"742",
GeoDist==0.969777832~"743",
GeoDist==0.078122909~"744",
GeoDist==1.342621985~"745",
GeoDist==0.407680362~"746",
GeoDist==1.031671489~"747",
GeoDist==0.860914404~"748",
GeoDist==0.867130971~"749",
GeoDist==0.168130201~"750",

GeoDist==1.735619627~"751",
GeoDist==0.418213219~"752",
GeoDist==1.874808405~"753",
GeoDist==0.627410109~"754",
GeoDist==0.519067638~"755",
GeoDist==1.371982878~"756",
GeoDist==0.818525548~"757",
GeoDist==2.618519807~"758",
GeoDist==1.396076394~"759",
GeoDist==0.10845097~"760",
GeoDist==0.998621694~"761",
GeoDist==1.250962255~"762",
GeoDist==2.100251691~"763",
GeoDist==0.362501016~"764",
GeoDist==1.397579778~"765",
GeoDist==3.125923628~"766",
GeoDist==1.600018309~"767",
GeoDist==0.982540605~"768",
GeoDist==0.438605052~"769",
GeoDist==0.566552574~"770",
GeoDist==1.170443216~"771",
GeoDist==2.367627211~"772",
GeoDist==1.542841674~"773",
GeoDist==1.705642256~"774",
GeoDist==1.099843883~"775",
GeoDist==0.791730694~"776",
GeoDist==2.274324111~"777",
GeoDist==1.45185097~"778",
GeoDist==0.73969763~"779",
GeoDist==1.052263041~"780",
GeoDist==2.499527587~"781",
GeoDist==2.379545273~"782",
GeoDist==0.507780728~"783",
GeoDist==1.441347134~"784",
GeoDist==1.294446619~"785",
GeoDist==0.484359203~"786",
GeoDist==1.688981804~"787",
GeoDist==2.186579536~"788",
GeoDist==1.508496537~"789",
GeoDist==2.294593005~"790",
GeoDist==1.079046757~"791",
GeoDist==0.242808508~"792",
GeoDist==1.395845837~"793",
GeoDist==2.730936406~"794",
GeoDist==0.273275338~"795",
GeoDist==2.052823403~"796",
GeoDist==0.033041368~"797",
GeoDist==0.996597508~"798",

GeoDist==1.557725307~"799",
GeoDist==0.945320786~"800",
GeoDist==2.936436151~"801",
GeoDist==1.646613434~"802",
GeoDist==1.304459841~"803",
GeoDist==0.469186449~"804",
GeoDist==1.74492339~"805",
GeoDist==0.277959482~"806",
GeoDist==1.30660578~"807",
GeoDist==1.019550261~"808",
GeoDist==1.345795509~"809",
GeoDist==0.732363793~"810",
GeoDist==0.745910787~"811",
GeoDist==2.543755234~"812",
GeoDist==1.556627321~"813",
GeoDist==0.589139644~"814",
GeoDist==0.928862721~"815",
GeoDist==0.10295182~"816",
GeoDist==1.266210513~"817",
GeoDist==1.372419259~"818",
GeoDist==1.914935889~"819",
GeoDist==0.633662267~"820",
GeoDist==1.073230145~"821",
GeoDist==0.639113392~"822",
GeoDist==1.47960015~"823",
GeoDist==0.590695551~"824",
GeoDist==2.25068455~"825",
GeoDist==1.247097729~"826",
GeoDist==1.682059273~"827",
GeoDist==1.581338684~"828",
GeoDist==0.793162269~"829",
GeoDist==1.412359605~"830",
GeoDist==1.269541037~"831",
GeoDist==1.429057867~"832",
GeoDist==1.421734305~"833",
GeoDist==0.977568721~"834",
GeoDist==0.461891976~"835",
GeoDist==1.469029108~"836",
GeoDist==0.825651761~"837",
GeoDist==2.533872383~"838",
GeoDist==1.120920451~"839",
GeoDist==2.219065174~"840",
GeoDist==1.557179655~"841",
GeoDist==0.936407858~"842",
GeoDist==0.482234183~"843",
GeoDist==1.134794724~"844",
GeoDist==2.326367173~"845",
GeoDist==1.361388278~"846",

GeoDist==0.374226723~"847",
GeoDist==0.688716043~"848",
GeoDist==0.336467093~"849",
GeoDist==0.572229916~"850",
GeoDist==1.512626554~"851",
GeoDist==1.255034209~"852",
GeoDist==1.149014074~"853",
GeoDist==1.300174716~"854",
GeoDist==1.462239289~"855",
GeoDist==2.31310694~"856",
GeoDist==1.093349844~"857",
GeoDist==1.363156176~"858",
GeoDist==0.166063942~"859",
GeoDist==1.245055694~"860",
GeoDist==1.169817308~"861",
GeoDist==0.820404287~"862",
GeoDist==0.896754051~"863",
GeoDist==0.711564387~"864",
GeoDist==1.548211865~"865",
GeoDist==0.740720122~"866",
GeoDist==0.709098984~"867",
GeoDist==1.141707852~"868",
GeoDist==1.889791076~"869",
GeoDist==2.662163923~"870",
GeoDist==0.580020515~"871",
GeoDist==2.294938058~"872",
GeoDist==0.708518309~"873",
GeoDist==0.678007537~"874",
GeoDist==1.522102842~"875",
GeoDist==2.48641659~"876",
GeoDist==1.325191915~"877",
GeoDist==0.332193305~"878",
GeoDist==2.237203174~"879",
GeoDist==1.161776894~"880",
GeoDist==0.896976154~"881",
GeoDist==0.236184201~"882",
GeoDist==1.74107356~"883",
GeoDist==0.86702929~"884",
GeoDist==1.212603435~"885",
GeoDist==1.263216606~"886",
GeoDist==1.16087469~"887",
GeoDist==2.443235198~"888",
GeoDist==0.580688256~"889",
GeoDist==1.382913362~"890",
GeoDist==3.079460617~"891",
GeoDist==1.834483096~"892",
GeoDist==0.870681213~"893",
GeoDist==2.710319427~"894",

```
                    GeoDist==1.759955735~"895",
                    GeoDist==1.458436809~"896",
                    GeoDist==1.662256965~"897",
                    GeoDist==1.739470357~"898",
                    GeoDist==2.617150285~"899",
                    GeoDist==2.095194744~"900",
                    GeoDist==2.560592482~"901",
                    GeoDist==0.930589481~"902",
                    GeoDist==1.435236236~"903",
                    GeoDist==1.434937961~"904",)) -> df

write.csv(df, file = "200621_copiestest.csv")
#### end ####

copiesdf<-read.csv("200621_copiestest.csv")
copiesSIM<-copiesdf$SIM
copiesSOR<-copiesdf$SOR
copiesSNE<-copiesdf$SNE
logcopiescopies<-copiesdf$copies
copiesgeo<-copiesdf$GeoDist
copiesID<-copiesdf$contrastID

mod6<-glmer(copiesSIM~logcopiescopies+(1|copiesID),
      family=binomial, data=copiesdf, nAGQ = 9, control=glmerControl(optimizer="bobyqa"))#Singular
fit
summary(mod6)
Anova(mod6, type=(2))
par(mfrow=c(1,2))
plot(residuals(mod6)~fitted(mod6), main="residuals vs fitted")
qqnorm(residuals(mod6))
par(mfrow=c(1,1))
mod7<-glmer(copiesSOR~logcopiescopies+(1|copiesID),
      family=binomial, data=copiesdf, nAGQ = 9, control=glmerControl(optimizer="bobyqa"))#Singular
fit
summary(mod7)
Anova(mod7, type=(2))
par(mfrow=c(1,2))
plot(residuals(mod7)~fitted(mod7), main="residuals vs fitted")
qqnorm(residuals(mod7))
par(mfrow=c(1,1))

ggplot(, aes(y=copiesSOR, x=logcopiescopies))+
  geom_point(aes(color= ))+
  geom_smooth(method="glm")+
  theme_classic()
#Average copy number and diversity metric by site comparison
#avgcopies<-aggregate(copiesdf$copies~copiesdf$contrastID, copiesdf, mean)
#avgsim<-aggregate(copiesdf$SIM~copiesdf$contrastID, copiesdf, mean)
```

```
#avgsor<-aggregate(copiesdf$SOR~copiesdf$contrastID, copiesdf, mean)
#avgsne<-aggregate(copiesdf$SNE~copiesdf$contrastID, copiesdf, mean)
#one<-merge(avgsim, avgcopies, by="copiesdf$contrastID")
#two<-merge(avgsor, avgsne, by="copiesdf$contrastID")
#final<-merge(one, two, by="copiesdf$contrastID")
#write.csv(final, file="200729_copiesavg.csv")
finalcopiesdf<-read.csv("200729_copiesavg.csv")

avgsimlme<-finalcopiesdf$SIM
avgcopieslme<-finalcopiesdf$copies
avgsorlme<-finalcopiesdf$SOR
avgsnelme<-finalcopiesdf$SNE


mod10<-glm(avgsimlme~avgcopieslme,family=binomial, data=finalcopiesdf)
summary(mod10)
Anova(mod10)

ggplot(, aes(y=avgsimlme, x=avgcopieslme))+
  geom_point(aes(color= ))+
  geom_smooth(method="glm", method.args=list(family='binomial'))+
  labs(x="Average (log) number of Pd copies per Contrast", y="Average SIM per contrast")+
  theme_classic()

B5 <- glm(avgsnelme~avgcopieslme, family=binomial, data=finalcopiesdf)
summary(B5)
plot(avgsnelme~avgcopieslme, data=finalcopiesdf, type='n', bty="n", xlim=c(0,5),ylim=c(0, 0.1),
xlab="Average (Log) fungal load per Contrast ", ylab="Average SNE")
points(avgcopieslme, avgsnelme, pch=19, cex=0.75, col="black", bg="black")
axis(side=1, lwd=2)
axis(side=2, lwd=2)
title("C", adj=1, line=-26, cex.main=2)

pred <-seq (0,5,0.01)
B5.pred <- predict(B5, list(avgcopieslme=pred), type="response", data=DDRPos)
lines(pred, B5.pred, lty=2, col="blue", lwd=5)
mod11<-glm(avgsorlme~avgcopieslme,family=binomial, data=finalcopiesdf)
summary(mod11)
Anova(mod11)

ggplot(, aes(y=avgsorlme, x=avgcopieslme))+
  geom_point(aes(color= ))+
  geom_smooth(method="glm", method.args=list(family='binomial'))+
  labs(x="Average (log) number of Pd copies per Contrast", y="Average SOR per contrast")+
  theme_classic()

mod12<-glm(avgsnelme~avgcopieslme,family=binomial, data=finalcopiesdf)
summary(mod12)
```

```
Anova(mod12)

ggplot(, aes(y=avgsnelme, x=avgcopieslme))+
  geom_point(aes(color= ))+
  geom_smooth(method="glm", method.args=list(family='binomial'))+
  labs(x="Average (log) number of Pd copies per Contrast", y="Average SNE per contrast")+
  theme_classic()

####end####
##################################################################
#Betadiversity-Betadisper full
##################################################################
####DataPrep####
meta<-data[1:23]
otus<-data[24:23871]
colSums(data[24:23871])

colSums(otus)
i<-(colSums(otus, na.rm=T)!=0)
otusnonzero<-otus[,i]
otuzeros<-otus[,!i]
colSums(otuzeros)
colSums(otusnonzero)
oturemovezero<-otusnonzero
s<-colSums(oturemovezero)
summary(s)
#Remove 1s
colSums(oturemovezero)
i<-(colSums(oturemovezero, na.rm=T)!=1)
otusnon1<-oturemovezero[,i]
otu1<-oturemovezero[,!i]
colSums(otu1)
oturemoveones<-otusnon1
s2<-colSums(oturemoveones)
summary(s2)

PAotus<-decostand(x=oturemoveones, method="pa")
rownames(PAotus)<-data$Group
pair<-beta.pair(PAotus, index.family="sor")
SOR<-(pair$beta.sor)
SIM<-(pair$beta.sim)
SNE<-(pair$beta.sne)
pd<-as.factor(data$pd)
site<-data$site
year<-data$year
Eco3<-meta$Level3_Region
RegionPd<-meta$region_pd
####end####
```

```
####Betadisper####
betadisper_SOR_pd <-betadisper(SOR, pd, type= c("centroid"), bias.adjust=FALSE, add=FALSE)
plot(betadisper_SOR_pd, axes=c(1,2), cex= 0.7, col = NULL, hull = TRUE, ellipse = FALSE, segments = TRUE, seg.col = "grey", label = FALSE, label.cex = 1, main= "")
(permutest.betadisper<-permutest(betadisper_SOR_pd, pairwise=FALSE, permutations=999, parallel=getOption("mc.cores"),))
boxplot(betadisper_SOR_pd)
#Sig Pd larger spread
betadisper_SIM_pd <-betadisper(SIM, pd, type= c("centroid"), bias.adjust=FALSE, add=FALSE)
plot(betadisper_SIM_pd, axes=c(1,2), cex= 0.7, col = NULL, hull = TRUE, ellipse = FALSE, segments = TRUE, seg.col = "grey", label = FALSE, label.cex = 1, main= "")
(permutest.betadisper<-permutest(betadisper_SIM_pd, pairwise=FALSE, permutations=999, parallel=getOption("mc.cores"),))
boxplot(betadisper_SIM_pd)
#Non sig
betadisper_SNE_pd <-betadisper(SNE, pd, type= c("centroid"), bias.adjust=FALSE, add=FALSE)
plot(betadisper_SNE_pd, axes=c(1,2), cex= 0.7, col = NULL, hull = TRUE, ellipse = FALSE, segments = TRUE, seg.col = "grey", label = FALSE, label.cex = 1, main= "")
(permutest.betadisper<-permutest(betadisper_SNE_pd, pairwise=FALSE, permutations=999, parallel=getOption("mc.cores"),))
boxplot(betadisper_SNE_pd)
#not sig
betadisper_SOR_regionpd <-betadisper(SOR, RegionPd, type= c("centroid"), bias.adjust=FALSE, add=FALSE)
plot(betadisper_SOR_regionpd, axes=c(1,2), cex= 0.7, col = NULL, hull = TRUE, ellipse = FALSE, segments = TRUE, seg.col = "grey", label = TRUE, label.cex = 1, main= "")
(permutest.betadisper<-permutest(betadisper_SOR_regionpd, pairwise=FALSE, permutations=999, parallel=getOption("mc.cores"),))
boxplot(betadisper_SOR_regionpd)
boxplot(betadisper_SOR_regionpd, xlab = "Ecoregion:Pd status", ylab = "Distance to Centroid", main="SOR")

#Sig
Tukey1<-TukeyHSD(betadisper_SOR_regionpd)
Tukey1
plot(Tukey1)
plot(betadisper_SOR_regionpd$distance~ betadisper_SOR_regionpd$group)
df<-data.frame(cbind(betadisper_SOR_regionpd$group, betadisper_SOR_regionpd$distances))
p<-ggplot(df, aes(x=betadisper_SOR_regionpd$group, y=betadisper_SOR_regionpd$distances))+
  stat_boxplot(geom='errorbar', linetype=1, width=0.5)+
  geom_boxplot()+
  ylim(0.55,0.8)+
  geom_text(x=1, y=0.75, label="A", size=8)+
  geom_text(x=2, y=0.75, label="BC", size=8)+
  geom_text(x=3, y=0.75, label="B", size=8)+
  geom_text(x=4, y=0.75, label="C", size=8)+
  geom_text(x=5, y=0.75, label="C", size=8)+
  geom_text(x=6, y=0.75, label="BC", size=8)+
```

```
  xlab("Ecoregion Pd interaction")+
  ylab("Distance to Centroid")+
  theme_classic()+
  ggtitle("Total Beta Diversity")+
  theme(plot.title=element_text(size = 30))
p


betadisper_SIM_regionpd <-betadisper(SIM, RegionPd, type= c("centroid"), bias.adjust=FALSE,
add=FALSE)
plot(betadisper_SIM_regionpd, axes=c(1,2), cex= 0.7, col = NULL, hull = TRUE, ellipse = FALSE, segments
= TRUE, seg.col = "grey", label = FALSE, label.cex = 1, main= "")
(permutest.betadisper<-permutest(betadisper_SIM_regionpd, pairwise=FALSE, permutations=999,
parallel=getOption("mc.cores"),))
boxplot(betadisper_SIM_regionpd)
boxplot(betadisper_SIM_regionpd, xlab = "Ecoregion:Pd status", ylab = "Distance to Centroid",
main="SIM")

#  sig
Tukey2<-TukeyHSD(betadisper_SIM_regionpd)
Tukey2
plot(Tukey2)
df2<-data.frame(cbind(betadisper_SIM_regionpd$group, betadisper_SIM_regionpd$distances))
p<-ggplot(df2, aes(x=betadisper_SIM_regionpd$group, y=betadisper_SIM_regionpd$distances))+
  stat_boxplot(geom='errorbar', linetype=1, width=0.5)+
  geom_boxplot()+
  ylim(0.48,0.8)+
  geom_text(x=1, y=0.75, label="A", size=8)+
  geom_text(x=2, y=0.75, label="B", size=8)+
  geom_text(x=3, y=0.75, label="B", size=8)+
  geom_text(x=4, y=0.75, label="B", size=8)+
  geom_text(x=5, y=0.75, label="B", size=8)+
  geom_text(x=6, y=0.75, label="B", size=8)+
  xlab("Ecoregion Pd interaction")+
  ylab("Distance to Centroid")+
  theme_classic()+
  ggtitle("B")+
  theme(plot.title=element_text(size = 30))
p

betadisper_SNE_regionpd <-betadisper(SNE, RegionPd, type= c("centroid"), bias.adjust=FALSE,
add=FALSE)
plot(betadisper_SNE_regionpd, axes=c(1,2), cex= 0.7, col = NULL, hull = TRUE, ellipse = FALSE, segments
= TRUE, seg.col = "grey", label = FALSE, label.cex = 1, main= "")
(permutest.betadisper<-permutest(betadisper_SNE_regionpd, pairwise=FALSE, permutations=999,
parallel=getOption("mc.cores"),))
boxplot(betadisper_SNE_regionpd)
```

```
boxplot(betadisper_SNE_regionpd, xlab = "Ecoregion:Pd status", ylab = "Distance to Centroid",
main="SNE")
Tukey3<-TukeyHSD(betadisper_SNE_regionpd)
Tukey3
plot(Tukey3)
df3<-data.frame(cbind(betadisper_SNE_regionpd$group, betadisper_SNE_regionpd$distances))
p<-ggplot(df2, aes(x=betadisper_SNE_regionpd$group, y=betadisper_SNE_regionpd$distances))+
  stat_boxplot(geom='errorbar', linetype=1, width=0.5)+
  geom_boxplot()+
  ylim(0,0.09)+
  xlab("Ecoregion Pd interaction")+
  ylab("Distance to Centroid")+
  theme_classic()+
  ggtitle("C")+
  theme(plot.title=element_text(size = 30))
p

betadisper_SIM_Pdregion <-betadisper(SIM, Eco3, type= c("centroid"), bias.adjust=FALSE, add=FALSE)
plot(betadisper_SIM_Pdregion, axes=c(1,2), cex= 0.7, col = NULL, hull = TRUE, ellipse = FALSE, segments
= TRUE, seg.col = "grey", label = FALSE, label.cex = 1, main= "")
(permutest.betadisper<-permutest(betadisper_SIM_Pdregion, pairwise=FALSE, permutations=999,
parallel=getOption("mc.cores"),))
boxplot(betadisper_SIM_Pdregion, main="SIM-PdRegion")
#non sig
betadisper_SOR_Pdregion <-betadisper(SOR, Eco3, type= c("centroid"), bias.adjust=FALSE, add=FALSE)
plot(betadisper_SOR_Pdregion, axes=c(1,2), cex= 0.7, col = NULL, hull = TRUE, ellipse = FALSE, segments
= TRUE, seg.col = "grey", label = FALSE, label.cex = 1, main= "")
(permutest.betadisper<-permutest(betadisper_SOR_Pdregion, pairwise=FALSE, permutations=999,
parallel=getOption("mc.cores"),))
boxplot(betadisper_SOR_Pdregion, main="SOR-PdRegion")
#Non sig
betadisper_SNE_Pdregion <-betadisper(SNE, Eco3, type= c("centroid"), bias.adjust=FALSE, add=FALSE)
plot(betadisper_SNE_Pdregion, axes=c(1,2), cex= 0.7, col = NULL, hull = TRUE, ellipse = FALSE, segments
= TRUE, seg.col = "grey", label = FALSE, label.cex = 1, main= "")
(permutest.betadisper<-permutest(betadisper_SNE_Pdregion, pairwise=FALSE, permutations=999,
parallel=getOption("mc.cores"),))
boxplot(betadisper_SNE_Pdregion, main="SNE-PdRegion")
#Non sig
test<-read.csv("201017_test.csv")
regionI<-test$region_pd
SORdist<-test$SORdist
SIMdist<-test$SIMdist
SNEdist<-test$SNEdist
plot(regionI, SORdist)
p<-ggplot(test, aes(x=regionI, y=SORdist))+
  stat_boxplot(geom='errorbar', linetype=1, width=0.5)+
  geom_boxplot()+
  ylim(0.55,0.8)+
```

```
  geom_text(x=1, y=0.75, label="a", size=8)+
  geom_text(x=2, y=0.75, label="b", size=8)+
  geom_text(x=3, y=0.75, label="bc", size=8)+
  geom_text(x=4, y=0.75, label="b", size=8)+
  geom_text(x=5, y=0.75, label="c", size=8)+
  geom_text(x=6, y=0.75, label="bc", size=8)+
  xlab("")+
  ylab("Distance to Centroid")+
  theme_classic()+
  labs(title="Total Beta Diversity", subtitle ="A")+
  theme(plot.title=element_text(size = 18),plot.subtitle =element_text(size=20, hjust=1))
p
p1<-p+ scale_x_discrete(labels=c("Interior-"="-", "Interior+"="+", "Ridge and Valley -"="-",
                            "Ridge and Valley +"="+", "South West Appalachians -"= "-",
                            "South West Appalachians +"="+"))
p1+theme(axis.text.x=element_text(size=20))
p<-ggplot(test, aes(x=regionI, y=SIMdist))+
  stat_boxplot(geom='errorbar', linetype=1, width=0.5)+
  geom_boxplot()+
  ylim(0.48,0.8)+
  geom_text(x=1, y=0.75, label="a", size=8)+
  geom_text(x=2, y=0.75, label="b", size=8)+
  geom_text(x=3, y=0.75, label="b", size=8)+
  geom_text(x=4, y=0.75, label="b", size=8)+
  geom_text(x=5, y=0.75, label="b", size=8)+
  geom_text(x=6, y=0.75, label="b", size=8)+
  xlab("")+
  ylab("Distance to Centroid")+
  theme_classic()+
  labs(title="Turnover", subtitle="B")+
  theme(plot.title=element_text(size = 18),plot.subtitle=element_text(size=20, hjust=1))
p

p1<-p+ scale_x_discrete(labels=c("Interior-"="-", "Interior+"="+", "Ridge and Valley -"="-",
                            "Ridge and Valley +"="+", "South West Appalachians -"= "-",
                            "South West Appalachians +"="+"))
p1+theme(axis.text.x=element_text(size=20))

p<-ggplot(test, aes(x=regionI, y=SNEdist))+
  stat_boxplot(geom='errorbar', linetype=1, width=0.5)+
  geom_boxplot()+
  ylim(0,0.1)+
  geom_text(x=1, y=0.75, label="A", size=8)+
  geom_text(x=2, y=0.75, label="BC", size=8)+
  geom_text(x=3, y=0.75, label="B", size=8)+
  geom_text(x=4, y=0.75, label="C", size=8)+
  geom_text(x=5, y=0.75, label="C", size=8)+
  geom_text(x=6, y=0.75, label="BC", size=8)+
```

```
  xlab("Ecoregion P. destructans Interaction")+
  ylab("Distance to Centroid")+
  theme_classic()+
  labs(title="Nestedness", subtitle="C")+
  theme(plot.title=element_text(size = 18), plot.subtitle=element_text(size=20, hjust=1),
        axis.text.x =element_text(size=15, margin=margin(t=0, r=10, b=55, l=0)), axis.title.x
=element_text(size=10, face="bold"))
p
p1<-p+ scale_x_discrete(labels=c("Interior-"="-", "Interior+"="+", "Ridge and Valley -"="-",
                              "Ridge and Valley +"="+", "South West Appalachians -"= "-",
                              "South West Appalachians +"="+"))
p1+theme(axis.text.x=element_text(size=20))

####end####
####Adonis####
adonis(SOR~pd*Eco3+year+site, permutations=999, method='bray', strata=site)
#Eco3 year p <0.01 pd p <0.05
adonis(SIM~pd*Eco3+year+site, permutations=999, method='bray', strata=site)
#Pd year p <0.01 eco3 p <0.05
adonis(SNE~pd*Eco3+year+site, permutations=999, method='bray', strata=site)
#No sig

#NMDS pos
positiveEco3<-positive$Level3_Region
skin.nmdspos<-metaMDS(posoturemovezero, distance = "bray", binary=TRUE, k=4, try=20,
trymax=2000)
data.scorespos <- as.data.frame(scores(skin.nmdspos))
data.scorespos
data.scorespos$pd <- rownames(data.scorespos)
data.scorespos$positiveEco3 <- positiveEco3
data.scorespos$region <- positiveEco3
head(data.scorespos)
tail(data.scorespos)
species.scorespos <- as.data.frame(scores(skin.nmdspos, "species"))
species.scorespos$species <- rownames(species.scorespos)
head(species.scorespos)

f1<-ggplot(data=species.scorespos, aes(x=NMDS1, y=NMDS2))+
  geom_point(data=data.scorespos, aes(x=NMDS1, y=NMDS2, colour=positiveEco3, shape=), size=2)+
  scale_color_discrete(name="Ecoregion", labels=c("Interior", "Ridge and Valley", "South West
Appalachians"))+
  labs(colour="Ecoregion")+
  coord_fixed()+
  theme_classic()+
  stat_ellipse(data=data.scorespos, aes(x=NMDS1, y=NMDS2, colour=positiveEco3), type="t", size=1.5,
linetype=1, show.legend = FALSE, level=0.95)
f1
```

```
posInterior<-data.scorespos[data.scorespos$positiveEco3=="Interior",
][chull(data.scorespos[data.scorespos$positiveEco3=="Interior", c("NMDS1", "NMDS2")]), ]

posSW_apps<-data.scorespos[data.scorespos$positiveEco3=="SW_Apps",
][chull(data.scorespos[data.scorespos$positiveEco3=="SW_Apps", c("NMDS1", "NMDS2")]), ]
posRidge_valley<-data.scorespos[data.scorespos$positiveEco3=="Ridge_Valley",
][chull(data.scorespos[data.scorespos$positiveEco3=="Ridge_Valley", c("NMDS1", "NMDS2")]), ]

poshull<-rbind(posInterior, posSW_apps, posRidge_valley)
poshull


f1<-ggplot(data=species.scorespos, aes(x=NMDS1, y=NMDS2))+
  geom_polygon(data=poshull, aes(x=NMDS1, y=NMDS2, colour=positiveEco3, group=positiveEco3),
fill=NA)+
  geom_point(data=data.scorespos, aes(x=NMDS1, y=NMDS2, colour=positiveEco3, shape=positiveEco3),
size=2)+
  scale_color_discrete(name="Ecoregion", labels=c("Interior", "Ridge and Valley", "South West
Appalachians"))+
  labs(colour="Ecoregion")+
  coord_fixed()+
  xlim(-0.4,0.5)+
  ylim(-0.45,0.4)+
  theme_classic()+
  labs(title="Positive Bats", caption="A")+
  theme(plot.title=element_text(size = 25), plot.caption=element_text(size=20), legend.position =
"none")
f1

#NMDS neg
negativeEco3<-negative$Level3_Region
skin.nmds2<-metaMDS(negoturemovezero, distance = "bray", binary=TRUE, k=4, try=20, trymax=2000)
data.scoresneg <- as.data.frame(scores(skin.nmds2))
data.scoresneg
data.scoresneg$group <- rownames(data.scoresneg)
data.scoresneg$negativeEco3 <- negativeEco3
data.scoresneg$region <- negativeEco3
head(data.scoresneg)
tail(data.scoresneg)
species.scoresneg <- as.data.frame(scores(skin.nmds2, "species"))
species.scoresneg$species <- rownames(species.scoresneg)
head(species.scoresneg)

f1<-ggplot(data=species.scoresneg, aes(x=NMDS1, y=NMDS2))+
  geom_point(data=data.scoresneg, aes(x=NMDS1, y=NMDS2, colour=negativeEco3, shape=), size=2)+
  scale_color_discrete(name="Ecoregion", labels=c("Interior", "Ridge and Valley", "South West
Appalachians"))+
  labs(colour="Ecoregion")+
```

```
  coord_fixed()+
  theme_classic()+
  stat_ellipse(data=data.scoresneg, aes(x=NMDS1, y=NMDS2, colour=negativeEco3), type="t", size=1.5,
linetype=1, show.legend = FALSE, level=0.95)
f1

negInterior<-data.scoresneg[data.scoresneg$negativeEco3=="Interior",
][chull(data.scoresneg[data.scoresneg$negativeEco3=="Interior", c("NMDS1", "NMDS2")]), ]
negSW_apps<-data.scoresneg[data.scoresneg$negativeEco3=="SW_Apps",
][chull(data.scoresneg[data.scoresneg$negativeEco3=="SW_Apps", c("NMDS1", "NMDS2")]), ]
negRidge_valley<-data.scoresneg[data.scoresneg$negativeEco3=="Ridge_Valley",
][chull(data.scoresneg[data.scoresneg$negativeEco3=="Ridge_Valley", c("NMDS1", "NMDS2")]), ]
neghull<-rbind(negInterior, negSW_apps, negRidge_valley)
neghull

f1<-ggplot(data=species.scoresneg, aes(x=NMDS1, y=NMDS2))+
  geom_polygon(data=neghull, aes(x=NMDS1, y=NMDS2, colour=negativeEco3, group=negativeEco3),
fill=NA)+
  geom_point(data=data.scoresneg, aes(x=NMDS1, y=NMDS2, colour=negativeEco3,
shape=negativeEco3), size=2)+
  scale_color_discrete(name="Ecoregion", labels=c("Interior Plateau", "Ridge and Valley", "South West
Appalachians"))+
  labs(colour="Ecoregion")+
  coord_fixed()+
  xlim(-0.4,0.5)+
  ylim(-0.45,0.4)+
  theme_classic()+
  labs(title="Negative Bats", caption="B")+
  theme(plot.title=element_text(size = 25),plot.caption=element_text(size=20))
f1

####end####
```

Supplemental File 1D Metadata

Group	Date	site	Level3_Region	pd
CCB277	170316	crumpton_creek_slp	Interior	0
CCB034	170104	fountain	Interior	0
CCB742	180108	hazel_warp	Interior	0
CCB043	170105	mason	Interior	0
CCB003	161220	ament_cave	Interior	1
CCB769	180126	cane_sink	Interior	1
CCB054	170103	ESPY	Interior	1
CCB764	180126	cane_sink	Interior	1
CCB254	170201	cripps_mill_cave	Interior	1
CCB1869	190109	ESPY	Interior	1
CCB858	180124	knob_creek	Interior	1
CCB033	170104	fountain	Interior	1
CCB844	180122	ward_cave	Interior	1
CCB843	180122	ward_cave	Interior	1
CCB856	180124	knob_creek	Interior	1
CCB041	170105	mason	Interior	1
CCB1870	190109	ESPY	Interior	1
CCB768	180126	cane_sink	Interior	1
CCB697	180118	mason	Interior	1
CCB743	180108	hazel_warp	Interior	1
CCB812	180131	leonard_cave	Interior	1
CCB741	180108	hazel_warp	Interior	1
CCB1868	190109	ESPY	Interior	1
CCB836	180119	aunt_beck_simmons_cave	Interior	1
CCB056	170103	ESPY	Interior	1
CCB851	180124	knob_creek	Interior	1
CCB255	170201	cripps_mill_cave	Interior	1
CCB757	180118	north_spivey_cave	Interior	1
CCB849	180122	ward_cave	Interior	1
CCB692	180118	mason	Interior	1
CCB053	170103	ESPY	Interior	1
CCB850	180122	ward_cave	Interior	1
CCB256	170201	cripps_mill_cave	Interior	1
CCB280	170316	crumpton_creek_slp	Interior	1
CCB765	180126	cane_sink	Interior	1
CCB694	180118	mason	Interior	1
CCB045	170105	mason	Interior	1
CCB001	161220	ament_cave	Interior	1
CCB848	180122	ward_cave	Interior	1
CCB832	180118	bridgewater_cave	Interior	1
CCB854	180124	knob_creek	Interior	1
CCB831	180118	bridgewater_cave	Interior	1
CCB830	180118	bridgewater_cave	Interior	1

CCB857	180124	knob_creek	Interior	1
CCB853	180124	knob_creek	Interior	1
CCB1723	190228	Trusselcave	Interior	1
CCB1718	190227	wet_cave	Interior	1
CCB1721	190228	Trusselcave	Interior	1
CCB533	180213	wet_cave	Interior	1
CCB530	180213	wet_cave	Interior	1
CCB1717	190227	wet_cave	Interior	1
CCB1714	190227	wet_cave	Interior	1
CCB1716	190227	wet_cave	Interior	1
CCB534	180213	wet_cave	Interior	1
CCB1722	190228	Trusselcave	Interior	1
CCB605	180130	norris	Ridge_Valley	0
CCB591	180126	blythe_ferry	Ridge_Valley	0
CCB1191	180313	cherokee_reservior	Ridge_Valley	0
CCB1192	180313	cherokee_reservior	Ridge_Valley	0
CCB1988	190109	Hickory_tree_cave	Ridge_Valley	0
CCB560	180104	marble_dale_blackman_cave	Ridge_Valley	0
CCB564	180104	marble_dale_blackman_cave	Ridge_Valley	0
CCB565	180104	marble_dale_blackman_cave	Ridge_Valley	0
CCB567	180104	marble_dale_blackman_cave	Ridge_Valley	0
CCB568	180104	marble_dale_blackman_cave	Ridge_Valley	0
CCB570	180104	marble_dale_blackman_cave	Ridge_Valley	0
CCB221	170120	oaks_cave	Ridge_Valley	0
CCB222	170120	oaks_cave	Ridge_Valley	0
CCB574	180105	oaks_cave	Ridge_Valley	0
CCB576	180105	oaks_cave	Ridge_Valley	0
CCB714	180112	river_bluff_sculpture	Ridge_Valley	0
CCB715	180112	river_bluff_sculpture	Ridge_Valley	0
CCB716	180112	river_bluff_sculpture	Ridge_Valley	0
CCB1982	190109	river_bluff_sculpture	Ridge_Valley	0
CCB1983	190109	river_bluff_sculpture	Ridge_Valley	0
CCB1985	190109	river_bluff_sculpture	Ridge_Valley	0
CCB1986	190109	river_bluff_sculpture	Ridge_Valley	0
CCB483	180201	indian_grave_point_cave	Ridge_Valley	1
CCB1949	190115	norris	Ridge_Valley	1
CCB604	180130	norris	Ridge_Valley	1
CCB1942	190115	norris	Ridge_Valley	1
CCB1947	190115	norris	Ridge_Valley	1
CCB305	170123	norris	Ridge_Valley	1
CCB1950	190115	norris	Ridge_Valley	1
CCB603	180130	norris	Ridge_Valley	1
CCB306	170123	norris	Ridge_Valley	1
CCB1948	190115	norris	Ridge_Valley	1
CCB302	170123	norris	Ridge_Valley	1

CCB310	170123	norris	Ridge_Valley	1
CCB303	170123	norris	Ridge_Valley	1
CCB301	170123	norris	Ridge_Valley	1
CCB307	170123	norris	Ridge_Valley	1
CCB602	180130	norris	Ridge_Valley	1
CCB2013	190212	marble_dale_blackman_cave	Ridge_Valley	1
CCB2009	190212	marble_dale_blackman_cave	Ridge_Valley	1
CCB779	180227	marble_bluff	Ridge_Valley	1
CCB790	180307	ghost_cave	Ridge_Valley	1
CCB789	180307	ghost_cave	Ridge_Valley	1
CCB569	180104	marble_dale_blackman_cave	Ridge_Valley	1
CCB612	180131	eaves	Ridge_Valley	1
CCB335	170313	marble_dale_blackman_cave	Ridge_Valley	1
CCB562	180104	marble_dale_blackman_cave	Ridge_Valley	1
CCB1964	190213	marble_bluff	Ridge_Valley	1
CCB1677	190206	blythe_ferry	Ridge_Valley	1
CCB573	180105	oaks_cave	Ridge_Valley	1
CCB331	170313	marble_dale_blackman_cave	Ridge_Valley	1
CCB314	170131	marble_bluff	Ridge_Valley	1
CCB2007	190122	oaks_cave	Ridge_Valley	1
CCB585	180105	herdocoons	Ridge_Valley	1
CCB312	170131	marble_bluff	Ridge_Valley	1
CCB613	180131	eaves	Ridge_Valley	1
CCB2010	190212	marble_dale_blackman_cave	Ridge_Valley	1
CCB379	170214	blythe_ferry	Ridge_Valley	1
CCB364	170214	eaves	Ridge_Valley	1
CCB590	180126	blythe_ferry	Ridge_Valley	1
CCB2004	190122	oaks_cave	Ridge_Valley	1
CCB584	180105	herdocoons	Ridge_Valley	1
CCB586	180105	herdocoons	Ridge_Valley	1
CCB333	170313	marble_dale_blackman_cave	Ridge_Valley	1
CCB592	180126	blythe_ferry	Ridge_Valley	1
CCB1674	190206	blythe_ferry	Ridge_Valley	1
CCB1969	190213	marble_bluff	Ridge_Valley	1
CCB1676	190206	blythe_ferry	Ridge_Valley	1
CCB561	180104	marble_dale_blackman_cave	Ridge_Valley	1
CCB380	170214	blythe_ferry	Ridge_Valley	1
CCB785	180227	marble_bluff	Ridge_Valley	1
CCB1965	190213	marble_bluff	Ridge_Valley	1
CCB610	180131	eaves	Ridge_Valley	1
CCB1662	190205	Hill_cave	Ridge_Valley	1
CCB2011	190212	marble_dale_blackman_cave	Ridge_Valley	1
CCB782	180227	marble_bluff	Ridge_Valley	1
CCB1952	190123	eaves	Ridge_Valley	1
CCB2005	190122	oaks_cave	Ridge_Valley	1

CCB572	180105	oaks_cave	Ridge_Valley	1
CCB1675	190206	blythe_ferry	Ridge_Valley	1
CCB611	180131	eaves	Ridge_Valley	1
CCB377	170214	blythe_ferry	Ridge_Valley	1
CCB608	180131	eaves	Ridge_Valley	1
CCB607	180131	eaves	Ridge_Valley	1
CCB137	170206	ballplay_cave	Ridge_Valley	1
CCB332	170313	marble_dale_blackman_cave	Ridge_Valley	1
CCB2006	190122	oaks_cave	Ridge_Valley	1
CCB1665	190205	Hill_cave	Ridge_Valley	1
CCB781	180227	marble_bluff	Ridge_Valley	1
CCB1957	190123	eaves	Ridge_Valley	1
CCB313	170131	marble_bluff	Ridge_Valley	1
CCB787	180307	ghost_cave	Ridge_Valley	1
CCB2003	190122	oaks_cave	Ridge_Valley	1
CCB376	170214	blythe_ferry	Ridge_Valley	1
CCB1688	190212	ghost_cave	Ridge_Valley	1
CCB138	170206	ballplay_cave	Ridge_Valley	1
CCB1962	190213	marble_bluff	Ridge_Valley	1
CCB1164	180315	meyers_mine	Ridge_Valley	1
CCB2012	190212	marble_dale_blackman_cave	Ridge_Valley	1
CCB1690	190212	ghost_cave	Ridge_Valley	1
CCB224	170120	oaks_cave	Ridge_Valley	1
CCB366	170214	eaves	Ridge_Valley	1
CCB783	180227	marble_bluff	Ridge_Valley	1
CCB140	170206	ballplay_cave	Ridge_Valley	1
CCB315	170131	marble_bluff	Ridge_Valley	1
CCB378	170214	blythe_ferry	Ridge_Valley	1
CCB1955	190123	eaves	Ridge_Valley	1
CCB606	180131	eaves	Ridge_Valley	1
CCB361	170214	eaves	Ridge_Valley	1
CCB1664	190205	Hill_cave	Ridge_Valley	1
CCB1692	190212	ghost_cave	Ridge_Valley	1
CCB363	170214	eaves	Ridge_Valley	1
CCB1686	190212	ghost_cave	Ridge_Valley	1
CCB362	170214	eaves	Ridge_Valley	1
CCB334	170313	marble_dale_blackman_cave	Ridge_Valley	1
CCB1963	190213	marble_bluff	Ridge_Valley	1
CCB1666	190205	Hill_cave	Ridge_Valley	1
CCB791	180307	ghost_cave	Ridge_Valley	1
CCB244	170131	lost_creek_cave	SW_Apps	0
CCB108	180104	Bone_cave	SW_Apps	0
CCB750	180110	coriolis	SW_Apps	0
CCB1995	190117	Drycave	SW_Apps	0
CCB762	180115	gastens_moonshine_cave	SW_Apps	0

CCB323	170203	nickajack	SW_Apps	0
CCB324	170203	nickajack	SW_Apps	0
CCB325	170203	nickajack	SW_Apps	0
CCB326	170203	nickajack	SW_Apps	0
CCB327	170203	nickajack	SW_Apps	0
CCB329	170203	nickajack	SW_Apps	0
CCB330	170203	nickajack	SW_Apps	0
CCB620	180119	el_abismo	SW_Apps	1
CCB242	170131	lost_creek_cave	SW_Apps	1
CCB621	180119	el_abismo	SW_Apps	1
CCB628	180129	calfkiller_slp	SW_Apps	1
CCB627	180119	el_abismo	SW_Apps	1
CCB746	180110	coriolis	SW_Apps	1
CCB749	180110	coriolis	SW_Apps	1
CCB1993	190117	Drycave	SW_Apps	1
CCB1312	190124	signature	SW_Apps	1
CCB105	171228	phineasfinncave	SW_Apps	1
CCB682	180109	keith_cove_cave	SW_Apps	1
CCB084	170303	carleton	SW_Apps	1
CCB1703	190226	Solomonstemple	SW_Apps	1
CCB2001	190117	Drycave	SW_Apps	1
CCB1603	190124	east_fork_slp	SW_Apps	1
CCB1994	190117	Drycave	SW_Apps	1
CCB297	170320	signature	SW_Apps	1
CCB1604	190124	east_fork_slp	SW_Apps	1
CCB1705	190226	Solomonstemple	SW_Apps	1
CCB1309	190124	signature	SW_Apps	1
CCB701	180314	carleton	SW_Apps	1
CCB1602	190124	east_fork_slp	SW_Apps	1
CCB1712	190227	Drycave	SW_Apps	1
CCB086	170303	carleton	SW_Apps	1
CCB295	170320	signature	SW_Apps	1
CCB700	180314	carleton	SW_Apps	1
CCB2002	190117	Drycave	SW_Apps	1
CCB1710	190227	Drycave	SW_Apps	1
CCB1706	190226	Solomonstemple	SW_Apps	1
CCB129	170125	east_fork_slp	SW_Apps	1
CCB082	170303	carleton	SW_Apps	1
CCB298	170320	signature	SW_Apps	1
CCB1709	190227	Drycave	SW_Apps	1
CCB089	170303	carleton	SW_Apps	1
CCB698	180411	east_fork_slp	SW_Apps	1
CCB1317	190313	carleton	SW_Apps	1
CCB088	170303	carleton	SW_Apps	1
CCB294	170320	signature	SW_Apps	1

CCB081	170303	carleton	SW_Apps	1
CCB130	170125	east_fork_slp	SW_Apps	1
CCB083	170303	carleton	SW_Apps	1
CCB1707	190226	Solomonstemple	SW_Apps	1
CCB1319	190313	carleton	SW_Apps	1
CCB1711	190227	Drycave	SW_Apps	1
CCB1708	190227	Drycave	SW_Apps	1
CCB1314	190313	carleton	SW_Apps	1
CCB1605	190124	east_fork_slp	SW_Apps	1
CCB1316	190313	carleton	SW_Apps	1
CCB699	180411	east_fork_slp	SW_Apps	1
CCB681	180109	keith_cove_cave	SW_Apps	1
CCB1702	190226	Solomonstemple	SW_Apps	1
CCB805	180411	east_fork_slp	SW_Apps	1
CCB087	170303	carleton	SW_Apps	1
CCB803	180411	east_fork_slp	SW_Apps	1
CCB1315	190313	carleton	SW_Apps	1
CCB1318	190313	carleton	SW_Apps	1
CCB328	170203	nickajack	SW_Apps	1
CCB771	180215	hole_in_wall	SW_Apps	1
CCB074	170227	whiteside	SW_Apps	1
CCB076	170227	whiteside	SW_Apps	1
CCB773	180215	little_cedar_mtn	SW_Apps	1
CCB071	170227	whiteside	SW_Apps	1
CCB106	171228	phineasfinncave	SW_Apps	0
CCB1310	190124	signature	SW_Apps	0

Supplemental File 1E Modeling Results

Indicator taxa and Pd status

Formula: indicatorabund ~ indicatorpd + (1 | indicatorsite)

AIC	BIC	logLik	deviance	df.resid
7089.3	7099.9	-3541.7	7083.3	246

Scaled residuals:

Min	1Q	Median	3Q	Max
-8.307	-3.574	-0.701	2.385	25.139

Random effects:

Groups	Name	Variance	Std.Dev.
indicatorsite	(Intercept)	0.6389	0.7993

Number of obs: 249, groups: indicatorsite, 48

Fixed effects:

	Estimate	Std. Error	z value	Pr(>|z|)
(Intercept)	4.92493	0.11967	41.16	<2e-16 ***
indicatorpd	-1.67301	0.02663	-62.84	<2e-16 ***

Correlation of Fixed Effects:

(Intr)	indicatorpd	-0.158

Results of GLMM analysis comparing the abundance of *P. destructans* negative indicator taxa between *P. destrctans* negative and postive bats. Indicator taxa are significantly more abundant on *P. destructans* negative bats compared to postive bats.

Indicator taxa and log transformed copy number

Formula: indicatorcopiesnegabund ~ logcopiesindicatorneg + (1 | indicatorcopiesnegsite)

AIC	BIC	logLik	deviance	df.resid
5667.2	5677.3	-2830.6	5661.2	206

Scaled residuals:

Min	1Q	Median	3Q	Max
-8.7142	-3.4131	-0.6229	2.4343	24.8495

Random effects:

Groups	Name	Variance	Std.Dev.
indicatorcopiesnegsite	(Intercept)	0.8475	0.9206

Number of obs: 209, groups: indicatorcopiesnegsite, 43

Fixed effects:

Estimate	Std.	Error	z value	Pr(>|z|)
(Intercept)	3.517601	0.147386	23.87	<2e-16 ***
logcopiesindicatorneg	-0.079233	0.007349	-10.78	<2e-16 ***

Correlation of Fixed Effects:

(Intr)

lgcpsndctrn -0.212

Results of GLMM analysis comparing the abundance of *P. destructans* negative indicator taxa and log transformed fungal load. There is a significant negative relationship between fungal load and indicator abundance.

SIM distance decay

glm(formula = AvgSIM ~ avgdist * avgpd, family = binomial)

Deviance Residuals:

Min	1Q	Median	3Q	Max
-0.64621	-0.07054	0.0078	0.08761	0.43755

Coefficients:

Estimate	Std.	Error	z value	Pr(>|z|)
(Intercept)	2.34673	0.46065	5.094	3.5e-07 ***
avgdist	-0.04093	0.23091	-0.177	0.859
avgpd1	0.19987	0.52346	0.382	0.703
avgdist:avgpd1	0.12079	0.28889	0.418	0.676

Null deviance: 20.151 on 1109 degrees of freedom

Residual deviance: 18.427 on 1106 degrees of freedom

AIC: 170.4

Results of GLM analysis comparing the turnover component of beta diversity averaged by site contrast and geographic distance between samples. There is a non significant relationship between turnover and geographic distance between samples.

SOR Distance Decay

glm(formula = AvgSOR ~ avgdist * avgpd, family = binomial)

Deviance Residuals:

Min	1Q	Median	3Q	Max
-0.50989	-0.06133	0.00885	0.08196	0.3442

Coefficients:

	Estimate	Std. Error	z value	Pr(>|z|)
(Intercept)	2.88438	0.57053	5.056	4.29e-07 ***
avgdist	-0.07389	0.28024	-0.264	0.792
avgpd1	0.1119	0.64711	0.173	0.863
avgdist:avgpd1	0.16799	0.35302	0.476	0.634

Null deviance: 15.478 on 1109 degrees of freedom

Residual deviance: 14.318 on 1106 degrees of freedom

AIC: 110.9

Results of GLM analysis comparing total beta diversity averaged by site contrast and geographic distance between samples. There is a non significant relationship between total beta diversity and geographic distance between samples.

SNE distance decay

glm(formula = AvgSNE ~ avgdist * avgpd, family = binomial)

Deviance Residuals:

Min	1Q	Median	3Q	Max
-0.26238	-0.07817	-0.014	0.04531	0.62748

Coefficients:

	Estimate	Std. Error	z value	Pr(>|z|)
(Intercept)	-3.32815	0.73337	-4.538	5.68e-06 ***
avgdist	-0.02315	0.38091	-0.061	0.952
avgpd1	-0.33164	0.83942	-0.395	0.693
avgdist:avgpd1	-0.02499	0.47418	-0.053	0.958

Null deviance: 13.027 on 1109 degrees of freedom

Residual deviance: 12.464 on 1106 degrees of freedom

AIC: 64.707

Results of GLM analysis comparing the nestedness component of beta diversity averaged by site contrast and geographic distance between samples. There is a non significant relationship between nestedness and geographic distance between samples.

SIM copies

glm(formula = avgsimlme ~ avgcopieslme, family = binomial, data = finalcopiesdf)

Deviance Residuals:

Min	1Q	Median	3Q	Max
-0.51222	-0.06772	0.00549	0.08068	0.38425

Coefficients:

	Estimate	Std. Error	Z value	Pr(>|z|)
(Intercept)	2.56694	0.3336	7.695	1.42E-14***
avgcopieslme	0.03613	0.14089	0.256	0.798

Null deviance: 13.743 on 932 degrees of freedom

Residual deviance: 13.676 on 931 degrees of freedom

AIC: 131.88

Results of GLM analysis comparing the between site average turnover component of beta diversity and difference in log transformed fungal load between samples. There is a non significant relationship between site average dissimilarity (SIM) and difference in log transformed fungal load.

SOR copies

glm(formula = avgsorlme ~ avgcopieslme, family = binomial, data = finalcopiesdf)

Deviance Residuals:

Min	1Q	Median	3Q	Max
-0.55529	-0.05877	0.00916	0.07416	0.30793

Coefficients:

	Estimate	Std. Error	z value	Pr(>|z|)
(Intercept)	3.02283	0.41068	7.36	1.83e-13 ***
avgcopieslme	0.04115	0.17386	0.237	0.813

Null deviance: 11.293 on 932 degrees of freedom

Residual deviance: 11.236 on 931 degrees of freedom

AIC: 85.202

Results of GLM analysis comparing the between site total beta diversity and difference in log transformed fungal load between samples. There is a non significant relationship between site average dissimilarity (SOR) and difference in log transformed fungal load.

SNE copies

glm(formula = avgsnelme ~ avgcopieslme, family = binomial, data = finalcopiesdf)

Deviance Residuals:

Min	1Q	Median	3Q	Max
-0.22445	-0.06933	-0.01229	0.04461	0.35614
	**Estimate**	**Std. Error**	**z value**	**Pr(>|z|)**
(Intercept)	-3.6673	0.5463	-6.714	1.9e-11 ***
avgcopieslme	-0.0242	0.2299	-0.105	0.916

Null deviance: 8.1731 on 932 degrees of freedom

Residual deviance: 8.1620 on 931 degrees of freedom

AIC: 48.664

Results of GLM analysis comparing the between average nestedness component of beta diversity and difference in log transformed fungal load between samples. There is a non significant relationship between site average dissimilarity (SNE) and difference in log transformed fungal load.

Supplemental File 1F Compositional Analysis

Methods

Compositional analysis

In order to address issues associated with the compositional nature of microbiome datasets (Gloor et al. 2017), we followed the pipeline outlined by Gloor et al*.* (2017) and clr transformed the presubsampled data set with the *compositions* package in R (Van den Boogaart and Tolosana-Delgado 2008). In total the clr transformed OTU × sample matrix had 33 163 OTUs. Once these data have been clr transformed these values can then be used in regression analysis (Van den Boogaart and Tolosana-Delgado 2013). To explore how treating our data as compositional would impact the result of our analysis on the indicator taxa we re-ran the indicator analysis using the *multipatt* function in R package *indicspecies* (version 1.7.8; De Caceres et al. 2020) on presence absence transformed OTU dataset. We then used a generalized linear mixed-effects model (GLMM) with the *glmer* function (package *lme4*; Bates et al. 2020) and assumed a Poisson error structure. We set site as the random effect to account for nested data, and compared the clr-transformed abundance of significant indicator taxa of *P. destructans* negative bats across all samples. Additionally, we compared the amount of *P. destructans* present (number of copies determined through qPCR) to the abundance of indicator taxa using a GLMM assuming a Poisson error structure, with site set as the random effect.

Results

A total of 728 OTUs were identified as indicator taxa for *P. destructans* negative bats. The group of indicator OTUs for the *P. destructans* negative bats represents the

common taxa occurring across the individual microbial communities that contributed to the nestedness in metacommunity structure. OTUs indicative of *P. destructans* negative bats were significantly more abundant on *P. destructans* negative, relative to *P. destructans* positive bats (GLMM; z = -66.92, p ≤ 0.05; Supplemental Fig. 1A). Additionally, there was a significant negative relationship between log transformed fungal load and indicator taxa abundance (GLMM; z = -49.54, p ≤ 0.05; Supplemental Fig. 1B), with increased fungal load predictive of fewer indicator taxa.

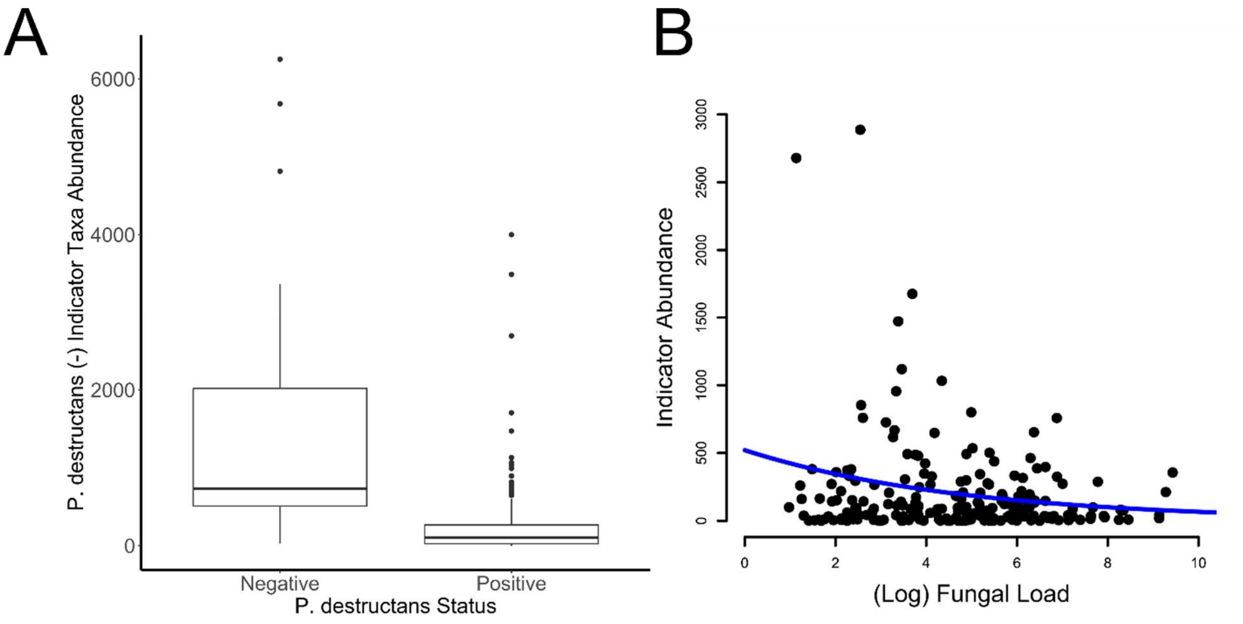

Supplemental Figure 1. A. Comparison of the abundance of *P. destructans* negative indicator taxa between *P. destructans* positive/negative samples. Indicator taxa are significantly more abundant within *P. destructans* negative samples (GLMM; z = -66.92, p ≤ 0.05). B. Comparison of the abundance of *P. destructans* negative indicator taxa by

fungal load. There is a significantly negative relationship between indicator taxa abundance and amount of *P. destructans* present (GLMM; $z = -49.54$, $p \leq 0.05$).

Literature Cited

De Caceres, M., Jansen, F. & De Caceres, M.M. ‘indicspecies’. R package version 1.7.9. https://CRAN.R-project.org/package=indicspecies (2020).

Bates, D., Sarkar, D., Bates, M.D. & Matrix, L. The lme4 package. R package version 1-1.26. https://CRAN.R-project.org/package=lme4 (2020).

Gloor, G.B., Macklaim, J.M., Pawlowsky-Glahn, V. & Egozcue, J.J. Microbiome datasets are compositional: and this is not optional. *Front Microbiol.* **8**, 2224; 10.3389/fmicb.2017.02224 (2017).

Van den Boogaart, K.G. & Tolosana-Delgado, R. Compositions: a unified R package to analyze compositional data. *Comput and Geosci.* **34**, 320-338 (2008).

Van den Boogaart, K.G. & Tolosana-Delgado, R. Analyzing compositional data with R (Springer International Publishing, 2013).

R Code

```
library(rcompanion)
library(compositions)
library(indicspecies)
library(dplyr)
library(ggplot2)
library(lme4)

stability<-read.table(file= "stability2.txt", header=TRUE)
metadata<-read.csv(file="PESU_site_5.csv")
data<-merge(metadata,stability, by="Group")
rownames(data)<-data$Group
set.seed(1138)
clr(stability[2:33186])
data<-merge(metadata, stability)
```

```
test<-colSums(data[24:33206])
indicator1<-subset(data, sp=="PESU")
meta<-indicator1[1:23]
indicator1_otus<-indicator1[24:33186]
colSums(indicator1_otus)
i<-(colSums(indicator1_otus, na.rm=T)!=0)
indicator1otusnonzero<-indicator1_otus[,i]
indicator1otuzeros<-indicator1_otus[,!i]
colSums(indicator1otuzeros)
indicator1oturemovezero<-indicator1otusnonzero


indicator1test<-multipatt(indicator1oturemovezero, indicator1$pd, func="IndVal.g",
control=how(nperm=999))
options(max.print = 10000)
summary(indicator1test)
indicator1<-select(data, "Group", "site", "copies",
"Pd","text","Otu000193","Otu000201","Otu000728","Otu003636","Otu000342","Otu0028
04","Otu002334","Otu000298","Otu000378","Otu003219","Otu001055","Otu006579","Ot
u004338","Otu003811","Otu002137","Otu001934","Otu001058","Otu000730","Otu00101
6","Otu006285","Otu002000","Otu006681","Otu004400","Otu002066","Otu005831","Otu
004475","Otu001091","Otu002391","Otu001537","Otu001460","Otu000362","Otu001473
","Otu000648","Otu005788","Otu001355","Otu003502","Otu001568","Otu005528","Otu0
06773","Otu005310","Otu004537","Otu002910","Otu002086","Otu008100","Otu019066"
,"Otu004162","Otu006669","Otu010716","Otu001333","Otu002683","Otu001551","Otu00
8888","Otu003321","Otu006098","Otu011427","Otu006476","Otu007789","Otu001012","
Otu002250","Otu004100","Otu010370","Otu000940","Otu001876","Otu003796","Otu000
722","Otu003886","Otu007227","Otu006380","Otu005985","Otu000410","Otu001527","
Otu006512","Otu004822","Otu002935","Otu001759","Otu001376","Otu000214","Otu006
940","Otu001102","Otu012378","Otu001304","Otu001640","Otu005278","Otu002685","
Otu002122","Otu004713","Otu002285","Otu002378","Otu005249","Otu010103","Otu004
693","Otu001007","Otu003827","Otu001449","Otu002278","Otu000524","Otu001644","
Otu014179","Otu000941","Otu000561","Otu012846","Otu004546","Otu012796","Otu001
638","Otu022126","Otu023742","Otu011269","Otu003723","Otu005808","Otu010807","
Otu002687","Otu009559","Otu004983","Otu006586","Otu009035","Otu006895","Otu002
110","Otu011986","Otu000875","Otu006101","Otu016392","Otu002244","Otu012904","
Otu006598","Otu008994","Otu012958","Otu002254","Otu003610","Otu006067","Otu008
626","Otu002336","Otu004336","Otu001235","Otu006348","Otu010325","Otu002811","
Otu008995","Otu003258","Otu003764","Otu012822","Otu025700","Otu025984","Otu014
004","Otu016102","Otu005377","Otu002270","Otu017020","Otu013386","Otu010733","
Otu008880","Otu001878","Otu007537","Otu009708","Otu011648","Otu003334","Otu010
472","Otu011773","Otu002069","Otu013570","Otu003178","Otu015437","Otu002934","
Otu002598","Otu003075","Otu001936","Otu012494","Otu015743","Otu009505","Otu008
301","Otu005735","Otu018795","Otu005354","Otu015330","Otu003530","Otu004588","
Otu002874","Otu014090","Otu004004","Otu007867","Otu010937","Otu012949","Otu008
664","Otu006835","Otu012267","Otu012700","Otu024140","Otu012840","Otu007563","
```

Otu003631","Otu017202","Otu001669","Otu002781","Otu003964","Otu010533","Otu019
520","Otu024595","Otu026010","Otu014778","Otu011304","Otu005173","Otu002656","
Otu002078","Otu004334","Otu003037","Otu012481","Otu005953","Otu007866","Otu016
277","Otu012087","Otu012156","Otu009245","Otu012302","Otu012326","Otu014252","
Otu016249","Otu021270","Otu001422","Otu006508","Otu013889","Otu003509","Otu013
674","Otu011970","Otu016090","Otu009986","Otu000817","Otu012053","Otu004768","
Otu003020","Otu013288","Otu015116","Otu008253","Otu003053","Otu003891","Otu013
696","Otu017646","Otu020924","Otu011145","Otu011995","Otu012634","Otu012284","
Otu018197","Otu000651","Otu002551","Otu002927","Otu004631","Otu004991","Otu005
603","Otu006585”,"Otu008525","Otu009383","Otu009908","Otu009937","Otu013724","
Otu013963","Otu014464","Otu017238","Otu019003","Otu019132","Otu020522","Otu021
106”,"Otu021171","Otu021386","Otu022367","Otu022651","Otu024514","Otu024757","
Otu025203","Otu025471","Otu026674","Otu028228","Otu028289","Otu004302",
"Otu004787","Otu005339","Otu003738","Otu013497","Otu006597","Otu005635","Otu00
2202","Otu008375","Otu017653","Otu004051","Otu007300","Otu014407","Otu005664","
Otu012487","Otu019651","Otu010939","Otu002191","Otu007451","Otu003210","Otu017
771","Otu025709","Otu026721","Otu027655","Otu017904","Otu005178","Otu003846","
Otu002944","Otu003804","Otu016312","Otu017595","Otu005787","Otu005317","Otu017
615","Otu021329","Otu025970","Otu027432","Otu030427","Otu001841","Otu013061","
Otu011384","Otu018786","Otu003473","Otu001301","Otu003683","Otu016830","Otu024
795","Otu025093","Otu008291","Otu002936","Otu004702","Otu003116","Otu019287","
Otu013535","Otu025244","Otu018171","Otu011845","Otu010699","Otu012198","Otu015
647","Otu031163","Otu031958","Otu004690","Otu014869","Otu004083","Otu011104","
Otu018689","Otu008349","Otu020024","Otu003245","Otu010830","Otu002419","Otu022
934","Otu023666","Otu026305","Otu028762","Otu030143","Otu010063","Otu012171","
Otu004028","Otu014317","Otu020537","Otu000210","Otu005556","Otu017413","Otu008
979","Otu008946","Otu007440","Otu016384","Otu016438","Otu016474","Otu017023","
Otu018169","Otu020569","Otu022013","Otu022488","Otu026089","Otu006956","Otu002
886","Otu007395","Otu004699","Otu015445","Otu016631","Otu006240","Otu008207","
Otu012730","Otu014216","Otu014819","Otu009943","Otu010768","Otu005778","Otu022
121","Otu025915","Otu026667","Otu012075","Otu012658","Otu008021","Otu001149","
Otu012253","Otu009695","Otu012382","Otu014946","Otu020267","Otu031244","Otu003
708","Otu002448","Otu017802","Otu018305","Otu004555","Otu015949","Otu019625","
Otu020754","Otu024272","Otu028295","Otu029410","Otu010969","Otu015617","Otu005
432","Otu005553","Otu008669","Otu019880","Otu021427","Otu023044","Otu023711","
Otu024654","Otu004615","Otu011721","Otu016356","Otu014804","Otu001689","Otu009
296","Otu013581","Otu013913","Otu013956","Otu014306","Otu016467","Otu017357","
Otu018223","Otu018371","Otu019857","Otu019872","Otu021672","Otu021727","Otu022
017","Otu024651","Otu024713","Otu025187","Otu025240","Otu025890","Otu026140","
Otu026720","Otu030249","Otu030595","Otu030617","Otu032143","Otu032521","Otu006
289","Otu005694","Otu003411","Otu002097","Otu003089","Otu007767","Otu003550","
Otu009588","Otu018211","Otu014807","Otu002233","Otu006516","Otu020362","Otu026
127","Otu030444","Otu009329","Otu010286","Otu022830","Otu001492","Otu005549","
Otu006566","Otu004531","Otu019824","Otu003538","Otu018834","Otu005997","Otu003
134","Otu003450","Otu003455","Otu003818","Otu004251","Otu004333","Otu004406","
Otu004805","Otu004933","Otu005188","Otu005547","Otu005691","Otu006051","Otu006

078","Otu006145","Otu006321","Otu006427","Otu006576","Otu006610","Otu006789","
Otu006851","Otu007120","Otu007191","Otu007305","Otu007324","Otu007444","Otu007
510","Otu007528","Otu007547","Otu008285","Otu008584","Otu008668","Otu008716","
Otu008862","Otu008953","Otu009105","Otu009220","Otu009317","Otu009547","Otu009
641","Otu009689","Otu009713","Otu009779","Otu009898","Otu010061","Otu010131","
Otu010270","Otu010332","Otu010366","Otu010567","Otu011401","Otu011610","Otu011
654","Otu011672","Otu011712","Otu011798","Otu012118","Otu012496","Otu012850","
Otu013364","Otu013767","Otu013792","Otu014129","Otu014325","Otu014338","Otu014
507","Otu014654","Otu014673","Otu014709","Otu015165","Otu015522","Otu015723","
Otu016481","Otu016797","Otu016858","Otu016970","Otu017779","Otu018102","Otu018
521","Otu018632","Otu018633","Otu019178","Otu019363","Otu019970","Otu020123","
Otu020405","Otu020439","Otu021217","Otu021248","Otu021546","Otu021574","Otu021
697","Otu021954","Otu022107","Otu022184","Otu022285","Otu022302","Otu022353","
Otu022444","Otu022609","Otu023483","Otu023943","Otu024227","Otu024448","Otu024
456","Otu024491","Otu024647","Otu025394","Otu025430","Otu025995","Otu026226","
Otu026276","Otu026547","Otu026977","Otu027212","Otu027320","Otu027629","Otu027
769","Otu028297","Otu029048","Otu029354","Otu029406","Otu029606","Otu029791","
Otu029820","Otu029994","Otu030251","Otu030930","Otu030981","Otu031259","Otu031
526","Otu031590","Otu031658","Otu032266","Otu032455","Otu032759","Otu033037","
Otu033189","Otu033200","Otu033271","Otu033360","Otu033458","Otu033514","Otu033
578","Otu033768","Otu034127","Otu034250","Otu001514","Otu004234","Otu002414","
Otu005790","Otu003916","Otu004545","Otu005446","Otu008611","Otu007646","Otu005
857","Otu008922","Otu002766","Otu008743","Otu003151","Otu002678","Otu006998","
Otu012677","Otu005434","Otu018198","Otu006378","Otu013395","Otu012858","Otu006
632","Otu009022","Otu007763","Otu008984","Otu004261","Otu006862","Otu006206","
Otu016461","Otu009979","Otu007107","Otu003031","Otu005781","Otu009862","Otu016
751","Otu012616","Otu016987","Otu003950","Otu006638","Otu007047","Otu016017","
Otu004411","Otu012014","Otu023681","Otu004329","Otu007766","Otu005064","Otu002
920","Otu003477","Otu009753","Otu005548","Otu006646","Otu017179","Otu021708","
Otu011992","Otu006117","Otu009504","Otu021275","Otu022380","Otu023163","Otu026
233","Otu026489","Otu026685","Otu015127","Otu005374","Otu000428","Otu019780","
Otu029164","Otu030325","Otu030737","Otu003607","Otu015216","Otu004226","Otu002
670","Otu016585","Otu007778","Otu018362","Otu020276","Otu021218","Otu022326","
Otu023774","Otu027469","Otu028075","Otu029037","Otu030196","Otu030544","Otu030
793","Otu030963","Otu031209","Otu031864","Otu032006","Otu032877","Otu033167","
Otu033336","Otu033698","Otu003894","Otu006736","Otu021934","Otu022799","Otu024
051","Otu024443","Otu029744","Otu031440","Otu032052","Otu033828","Otu004618","
Otu006528","Otu020344","Otu006947","Otu006987","Otu009234","Otu017360","Otu017
528","Otu022165","Otu007097","Otu011086","Otu004163","Otu019844","Otu020798","
Otu021631","Otu021791","Otu023532","Otu023835","Otu023949","Otu025234","Otu026
590","Otu028477","Otu031375","Otu013182","Otu018631","Otu002997","Otu001468","
Otu002131","Otu004434","Otu011662","Otu013246","Otu017846","Otu023341","Otu025
986","Otu030563","Otu012956","Otu023473","Otu023718","Otu008267","Otu011795","
Otu014942","Otu014019","Otu004374","Otu015631","Otu018774","Otu019665","Otu019
904","Otu020898","Otu025144","Otu027395","Otu027692","Otu031655","Otu032239","
Otu033276","Otu003727","Otu007639","Otu008302","Otu004737","Otu011807","Otu018

```
272","Otu004598","Otu023644","Otu005086","Otu010403","Otu025932","Otu026465","
Otu013410","Otu019268")

indicatortext<-indicator1$text
indicatorpd<-indicator1$pd
test<-colSums(indicator1[6:368])
summary(test)#Zero Otus are not found in at least one sample(colSums min=2)
indicatorpd<-indicator1$Pd
indicatorsite<-indicator1$site
rowSums(indicator1[6:368])
indicatorabund<-rowSums(indicator1[6:799])
indicator1PA<-indicator1[6:799]
indicator1PA[indicator1PA > 0] <- 1
PAabund<-rowSums(indicator1PA)
boxplot(indicatorabund~indicatorpd)
plotNormalHistogram(indicatorabund[indicatorpd=="1"])
plotNormalHistogram(indicatorabund[indicatorpd=="0"])

m1<-glmer(indicatorabund~indicatorpd+(1|indicatorsite), family=poisson)
op<-par(mfrow=c(1,2))
qqnorm(resid(m1))#This fit isn't great but still acceptable
qqline(resid(m1))
plot(residuals(m1)~fitted(m1), main="residuals vs fitted")
op<-par(mfrow=c(1,1))
summary(m1)
svg("FigA.svg")
p<-ggplot(indicator1, aes(x=indicatortext, y=indicatorabund, group=text))+
  geom_boxplot()+
  labs(x="P. destructans Status", y="P. destructans (-) Indicator Taxa Abundance")+
  theme( plot.caption = element_text(size=28, face="bold"),
        #panel.border = element_blank(),
        #panel.grid.major = element_blank(),
        #panel.grid.minor=element_blank(),
        panel.background  = element_blank(),
        axis.text=element_text(size=17),
        axis.title=element_text(size=18),
        axis.line=element_line(size=1, colour="black"))
p
dev.off()
indicatorcopiesneg<-subset(indicator1, Pd=="1", header=T)
logcopiesindicatorneg<-log(indicatorcopiesneg$copies+1)
indicatorcopiesnegabund<-rowSums(indicatorcopiesneg[6:368])
indicatorcopiesnegsite<-indicatorcopiesneg$site
plot(logcopiesindicatorneg, indicatorcopiesnegabund)
indicatorcopyPA<-indicatorcopiesneg[6:368]
indicatorcopyPA[indicatorcopyPA > 0] <- 1
```

```
copyPAabund<-rowSums(indicatorcopyPA)
plot(copyPAabund~logcopiesindicatorneg)

m4<-glmer(indicatorcopiesnegabund~logcopiesindicatorneg+(1|indicatorcopiesnegsite),
family = poisson)
op<-par(mfrow=c(1,2))
qqnorm(resid(m4))
qqline(resid(m4))
plot(residuals(m4)~fitted(m4), main="residuals vs fitted")
op<-par(mfrow=c(1,1))
summary(m4)

svg("FigB.svg")
B1 <- glm(indicatorcopiesnegabund~logcopiesindicatorneg, family=poisson)
summary(B1)
plot(indicatorcopiesnegabund~logcopiesindicatorneg, type='n', bty="n", xlim=c(0,10),
ylim=c(0,3000),xlab="(Log) Fungal Load", ylab="Indicator Abundance",cex.lab=1.7)
points(logcopiesindicatorneg, indicatorcopiesnegabund, pch=19, cex=1.5, col="black",
bg="black")
axis(side=1, lwd=3)
axis(side=2, lwd=3)
#title("B", adj=1, line=-26, cex.main=2)
pred <-seq (0,10.4,0.01)
B1.pred <- predict(B1, list(logcopiesindicatorneg=pred), type="response",
data=indicatorcopiesnegdata)
lines(pred, B1.pred, lty=1, col="blue", lwd=5)
#tiff("test.tiff", width = 4, height = 4, units = 'in', res = 200)
dev.off()
```

Supplemental File 1G Betadisper Results

***P. destructans* Positive and Negative Bats**

**Ecoregion**	**p-value**
Total Beta Diversity	0.884
Turnover	0.97
Nestedness	0.603
***P. destructans* status**	**p-value**
Total Beta Diversity	0.012*
Turnover	0.175
Nestedness	0.383
***P. destructans* status:Ecoregion**	**p-value**
Total Beta Diversity	0.001**
Turnover	0.001**
Nestedness	0.511

Betadisper analysis comparing beta diversity measured as multivariate dispersion across ecoregions, *P. destructans* status, and interactive effect of *P. destructans* status and ecoregion (*P. destructans* status:Ecoregion). ** indicates a significant difference with a p value of 0.001 * indicates a significant difference with a p value of 0.05. Ecoregion corresponds to EPA ecoregion 3 (specifically Interior Plateau, South West Appalachians, and Ridge and Valley).
