# Supplementary material for: The presence of Pseudogymnoascus destructans, a fungal pathogen of bats, correlates with changes in microbial metacommunity structure
Source: Sci Rep. 2021 Jun 3;11:11685. doi: 10.1038/s41598-021-91118-1 (PMC8175404; doi:10.1038/s41598-021-91118-1)
Supplement: Supplementary file 2 — Supplementary Information 2. [file 41598_2021_91118_MOESM2_ESM.pdf]

Title: The presence of a fungal pathogen correlates with changes in microbial metacommunity structure

Matthew Grisnik1, Joshua B Grinath2, Donald M Walker1*
1Middle Tennessee State University, Department of Biology, Murfreesboro, Tennessee 37132, USA
2Idaho State University, Department of Biological Sciences, Pocatello, Idaho 83209, USA
*Corresponding author
Donald M. Walker, PhD
Donald.Walker@mtsu.edu
615-904-8382

P. destructans negative Indicator taxa

OTU	Kingdom	Phylum				
Otu002086	Bacteria	Acidobacteria	Blastocatellia_	11-24_fa	11-24_fa	11-24_ge
Otu002414	Bacteria	Acidobacteria	Blastocatellia_	11-24_fa	11-24_fa	11-24_ge
Otu004546	Bacteria	Acidobacteria	Blastocatellia_	11-24_fa	11-24_fa	11-24_ge
Otu005664	Bacteria	Acidobacteria	Blastocatellia_	11-24_fa	11-24_fa	11-24_ge
Otu007120	Bacteria	Acidobacteria	Blastocatellia_	11-24_fa	11-24_fa	11-24_ge
Otu006638	Bacteria	Acidobacteria	Blastocatellia_	Blastocatellales	Blastocatellaceae	Blastocatellaceae_unclassified
Otu004234	Bacteria	Acidobacteria	Blastocatellia_	Blastocatellia_ _unclassifie	Blastocatellia_ _unclassifie	Blastocatellia_ _unclassified
Otu002874	Bacteria	Acidobacteria	Blastocatellia_	DS-100	DS-100_fa	DS-100_ge
Otu003031	Bacteria	Acidobacteria	FFCH5909	FFCH5909_or	FFCH5909_fa	FFCH5909_ge
Otu000374	Bacteria	Acidobacteria	Blastocatellia_	Pyrinomonadales	Pyrinomonadaceae	RB41
Otu000722	Bacteria	Acidobacteria	Blastocatellia_	Pyrinomonadales	Pyrinomonadaceae	RB41
Otu001333	Bacteria	Acidobacteria	Blastocatellia_	Pyrinomonadales	Pyrinomonadaceae	RB41
Otu001759	Bacteria	Acidobacteria	Blastocatellia_	Pyrinomonadales	Pyrinomonadaceae	RB41
Otu002378	Bacteria	Acidobacteria	Blastocatellia_	Pyrinomonadales	Pyrinomonadaceae	RB41
Otu004302	Bacteria	Acidobacteria	Blastocatellia_	Pyrinomonadales	Pyrinomonadaceae	RB41
Otu009713	Bacteria	Acidobacteria	Blastocatellia_	Pyrinomonadales	Pyrinomonadaceae	RB41
Otu030143	Bacteria	Acidobacteria	Blastocatellia_	Pyrinomonadales	Pyrinomonadaceae	RB41
Otu008525	Bacteria	Acidobacteria	Acidobacteriia	Solibacterales	Solibacteraceae_	Solibacteraceae_ _unclassified
Otu001040	Bacteria	Acidobacteria	Blastocatellia_	Blastocatellales	Blastocatellaceae	Stenotrophobacter
Otu001876	Bacteria	Acidobacteria	Thermoanaerobaculia	Thermoanaerobaculales	Thermoanaerobaculaceae	Subgroup_10
Otu002202	Bacteria	Acidobacteria	Acidobacteriia	Subgroup_13	Subgroup_13_fa	Subgroup_13_ge
Otu007778	Bacteria	Acidobacteria	Subgroup_17	Subgroup_17_or	Subgroup_17_fa	Subgroup_17_ge
Otu008668	Bacteria	Acidobacteria	Subgroup_22	Subgroup_22_or	Subgroup_22_fa	Subgroup_22_ge
Otu001644	Bacteria	Acidobacteria	Subgroup_6	Subgroup_6_or	Subgroup_6_fa	Subgroup_6_ge
Otu010718	Bacteria	Acidobacteria	Subgroup_6	Subgroup_6_or	Subgroup_6_fa	Subgroup_6_ge
Otu003727	Bacteria	Acidobacteria	Subgroup_6	Subgroup_6_unclassified	Subgroup_6_unclassified	Subgroup_6_unclassified
Otu004537	Bacteria	Acidobacteria	Subgroup_6	Subgroup_6_unclassified	Subgroup_6_unclassified	Subgroup_6_unclassified
Otu004374	Bacteria	Acidobacteria	Acidobacteriia	Acidobacteriales	uncultured	uncultured_ge
Otu001186	Bacteria	Actinobacteria	Thermoleophilia	Solirubrobacterales	67-14	67-14_ge
Otu002927	Bacteria	Actinobacteria	Thermoleophilia	Solirubrobacterales	67-14	67-14_ge
Otu003827	Bacteria	Actinobacteria	Thermoleophilia	Solirubrobacterales	67-14	67-14_ge
Otu006280	Bacteria	Actinobacteria	Thermoleophilia	Solirubrobacterales	67-14	67-14_ge
Otu001304	Bacteria	Actinobacteria	Thermoleophilia	Solirubrobacterales	67-14	67-14_ge
Otu003325	Bacteria	Actinobacteria	Acidimicrobiia	Acidimicrobiia_unclassifie	Acidimicrobiia_unclassified	Acidimicrobiia_unclassified
Otu004261	Bacteria	Actinobacteria	Acidimicrobiia	Acidimicrobiia_unclassifie	Acidimicrobiia_unclassified	Acidimicrobiia_unclassified
Otu005169	Bacteria	Actinobacteria	Acidimicrobiia	Acidimicrobiia_unclassifie	Acidimicrobiia_unclassified	Acidimicrobiia_unclassified
Otu031864	Bacteria	Actinobacteria	Acidimicrobiia	Acidimicrobiia_unclassifie	Acidimicrobiia_unclassified	Acidimicrobiia_unclassified
Otu000604	Bacteria	Actinobacteria	Actinobacteria	Actinobacteria_unclassifie	Actinobacteria_unclassifie	Actinobacteria_unclassified
Otu002233	Bacteria	Actinobacteria	Actinobacteria	Actinobacteria_unclassifie	Actinobacteria_unclassifie	Actinobacteria_unclassified
Otu004100	Bacteria	Actinobacteria	Actinobacteria	Actinobacteria_unclassifie	Actinobacteria_unclassifie	Actinobacteria_unclassified
Otu005831	Bacteria	Actinobacteria	Actinobacteria	Actinobacteria_unclassifie	Actinobacteria_unclassifie	Actinobacteria_unclassified
Otu007219	Bacteria	Actinobacteria	Actinobacteria	Actinobacteria_unclassifie	Actinobacteria_unclassifie	Actinobacteria_unclassified
Otu007767	Bacteria	Actinobacteria	Actinobacteria	Actinobacteria_unclassifie	Actinobacteria_unclassifie	Actinobacteria_unclassified
Otu007877	Bacteria	Actinobacteria	Actinobacteria	Actinobacteria_unclassifie	Actinobacteria_unclassifie	Actinobacteria_unclassified
Otu008454	Bacteria	Actinobacteria	Actinobacteria	Actinobacteria_unclassifie	Actinobacteria_unclassifie	Actinobacteria_unclassified
Otu011269	Bacteria	Actinobacteria	Actinobacteria	Actinobacteria_unclassifie	Actinobacteria_unclassifie	Actinobacteria_unclassified
Otu011384	Bacteria	Actinobacteria	Actinobacteria_unclassifi	Actinobacteria_unclassifie	Actinobacteria_unclassifie	Actinobacteria_unclassified

Otu025430	Bacteria	Actinobacteria	Actinobacteria	Actinobacteria_unclassifie	Actinobacteria_unclassifiec	Actinobacteria_unclassified
Otu000730	Bacteria	Actinobacteria	Actinobacteria	Propionibacteriales	Nocardioidaceae	Aeromicrobium
Otu006826	Bacteria	Actinobacteria	Actinobacteria	Propionibacteriales	Nocardioidaceae	Aeromicrobium
Otu008303	Bacteria	Actinobacteria	Actinobacteria	Propionibacteriales	Nocardioidaceae	Aeromicrobium
Otu004211	Bacteria	Actinobacteria	Thermoleophilia	Solirubrobacterales	Solirubrobacteraceae	Conexibacter
Otu002185	Bacteria	Actinobacteria	Thermoleophilia	Solirubrobacterales	Solirubrobacteraceae	Conexibacter
Otu008880	Bacteria	Actinobacteria	Actinobacteria	Corynebacteriales	Corynebacteriaceae	Corynebacteriaceae_unclassified
Otu004822	Bacteria	Actinobacteria	Actinobacteria	Pseudonocardiales	Pseudonocardiaceae	Crossiella
Otu002334	Bacteria	Actinobacteria	Actinobacteria	Pseudonocardiales	Pseudonocardiaceae	Crossiella
Otu004631	Bacteria	Actinobacteria	Actinobacteria	Pseudonocardiales	Pseudonocardiaceae	Crossiella
Otu002231	Bacteria	Actinobacteria	Actinobacteria	Pseudonocardiales	Pseudonocardiaceae	Crossiella
Otu010185	Bacteria	Actinobacteria	Actinobacteria	Pseudonocardiales	Pseudonocardiaceae	Crossiella
Otu006625	Bacteria	Actinobacteria	Actinobacteria	Propionibacteriales	Propionibacteriaceae	Cutibacterium
Otu002766	Bacteria	Actinobacteria	Actinobacteria	Micromonosporales	Micromonosporaceae	Dactylosporangium
Otu000342	Bacteria	Actinobacteria	Nitriliruptoria	Euzebyales	Euzebyaceae	Euzebyaceae_unclassified
Otu003731	Bacteria	Actinobacteria	Nitriliruptoria	Euzebyales	Euzebyaceae	Euzebyaceae_unclassified
Otu000290	Bacteria	Actinobacteria	Thermoleophilia	Gaiellales	Gaiellaceae	Gaiella
Otu000298	Bacteria	Actinobacteria	Thermoleophilia	Gaiellales	Gaiellaceae	Gaiella
Otu012378	Bacteria	Actinobacteria	Thermoleophilia	Gaiellales	Gaiellaceae	Gaiella
Otu001781	Bacteria	Actinobacteria	Thermoleophilia	Gaiellales	Gaiellaceae	Gaiella
Otu001301	Bacteria	Actinobacteria	Actinobacteria	Frankiales	Geodermatophilaceae	Geodermatophilus
Otu000820	Bacteria	Actinobacteria	Acidimicrobiia	IMCC26256	IMCC26256_fa	IMCC26256_ge
Otu002088	Bacteria	Actinobacteria	Acidimicrobiia	IMCC26256	IMCC26256_fa	IMCC26256_ge
Otu013395	Bacteria	Actinobacteria	Acidimicrobiia	IMCC26256	IMCC26256_fa	IMCC26256_ge
Otu013581	Bacteria	Actinobacteria	Acidimicrobiia	IMCC26256	IMCC26256_fa	IMCC26256_ge
Otu003029	Bacteria	Actinobacteria	Actinobacteria	Micrococcales	Intrasporangiaceae	Intrasporangiaceae_unclassified
Otu019079	Bacteria	Actinobacteria	Actinobacteria	Propionibacteriales	Nocardioidaceae	Marmoricola
Otu001934	Bacteria	Actinobacteria	Actinobacteria	Propionibacteriales	Nocardioidaceae	Marmoricola
Otu001616	Bacteria	Actinobacteria	MB-A2-108	MB-A2-108_or	MB-A2-108_fa	MB-A2-108_ge
Otu007300	Bacteria	Actinobacteria	MB-A2-108	MB-A2-108_or	MB-A2-108_fa	MB-A2-108_ge
Otu007440	Bacteria	Actinobacteria	MB-A2-108	MB-A2-108_or	MB-A2-108_fa	MB-A2-108_ge
Otu005377	Bacteria	Actinobacteria	Actinobacteria	Micrococcales	Microbacteriaceae	Microbacteriaceae_unclassified
Otu003886	Bacteria	Actinobacteria	Actinobacteria	Micrococcales	Microbacteriaceae	Microbacteriaceae_unclassified
Otu001507	Bacteria	Actinobacteria	Actinobacteria	Micromonosporales	Micromonosporaceae	Micromonosporaceae_unclassified
Otu004406	Bacteria	Actinobacteria	Acidimicrobiia	Microtrichales	Microtrichales_fa	Microtrichales_ge
Otu001016	Bacteria	Actinobacteria	Actinobacteria	Corynebacteriales	Nocardiaceae	Nocardia
Otu002137	Bacteria	Actinobacteria	Actinobacteria	Corynebacteriales	Nocardiaceae	Nocardia
Otu002110	Bacteria	Actinobacteria	Actinobacteria	Propionibacteriales	Nocardioidaceae	Nocardioides
Otu011409	Bacteria	Actinobacteria	Actinobacteria	Propionibacteriales	Propionibacteriales_unclas	Propionibacteriales_unclassified
Otu006512	Bacteria	Actinobacteria	Actinobacteria	Propionibacteriales	Propionibacteriales_unclas	Propionibacteriales_unclassified
Otu001007	Bacteria	Actinobacteria	Actinobacteria	Pseudonocardiales	Pseudonocardiaceae	Pseudonocardia
Otu001102	Bacteria	Actinobacteria	Actinobacteria	Pseudonocardiales	Pseudonocardiaceae	Pseudonocardiaceae_unclassified
Otu001390	Bacteria	Actinobacteria	Actinobacteria	Pseudonocardiales	Pseudonocardiaceae	Pseudonocardiaceae_unclassified
Otu006586	Bacteria	Actinobacteria	Actinobacteria	Pseudonocardiales	Pseudonocardiaceae	Pseudonocardiaceae_unclassified
Otu018786	Bacteria	Actinobacteria	Actinobacteria	Pseudonocardiales	Pseudonocardiaceae	Pseudonocardiaceae_unclassified
Otu026010	Bacteria	Actinobacteria	Actinobacteria	Pseudonocardiales	Pseudonocardiaceae	Pseudonocardiaceae_unclassified
Otu020898	Bacteria	Actinobacteria	Actinobacteria	Pseudonocardiales	Pseudonocardiaceae	Pseudonocardiaceae_unclassified
Otu002723	Bacteria	Actinobacteria	Actinobacteria	Corynebacteriales	Nocardiaceae	Rhodococcus
Otu001449	Bacteria	Actinobacteria	Rubrobacteria	Rubrobacterales	Rubrobacteriaceae	Rubrobacter
Otu004336	Bacteria	Actinobacteria	Rubrobacteria	Rubrobacterales	Rubrobacteriaceae	Rubrobacter
Otu002244	Bacteria	Actinobacteria	Thermoleophilia	Solirubrobacterales	Solirubrobacteraceae	Solirubrobacteraceae_unclassified
Otu001492	Bacteria	Actinobacteria	Thermoleophilia	Solirubrobacterales	Solirubrobacterales_unclas	Solirubrobacterales_unclassified
Otu006862	Bacteria	Actinobacteria	Thermoleophilia	Solirubrobacterales	Solirubrobacterales_unclas	Solirubrobacterales_unclassified
Otu011970	Bacteria	Actinobacteria	Thermoleophilia	Solirubrobacterales	Solirubrobacterales_unclas	Solirubrobacterales_unclassified
Otu003408	Bacteria	Actinobacteria	Thermoleophilia	Solirubrobacterales	Solirubrobacterales_unclas	Solirubrobacterales_unclassified
Otu013963	Bacteria	Actinobacteria	Thermoleophilia	Solirubrobacterales	Solirubrobacterales_unclas	Solirubrobacterales_unclassified
Otu013688	Bacteria	Actinobacteria	Actinobacteria	Frankiales	Sporichthyaceae	Sporichthya
Otu002434	Bacteria	Actinobacteria	Actinobacteria	Streptomycetales	Streptomycetaceae	Streptomyces

Otu016751	Bacteria	Actinobacteria	Actinobacteria	Streptomycetales	Streptomycetaceae	Streptomycetaceae_unclassified
Otu007763	Bacteria	Actinobacteria	Actinobacteria	Streptosporangiales	Streptosporangiaceae	Streptosporangiaceae_unclassified
Otu004983	Bacteria	Actinobacteria	Thermoleophilia	Thermoleophilia_unclassifi	Thermoleophilia_unclassifi	Thermoleophilia_unclassified
Otu015954	Bacteria	Actinobacteria	Thermoleophilia	Thermoleophilia_unclassifi	Thermoleophilia_unclassifi	Thermoleophilia_unclassified
Otu017462	Bacteria	Actinobacteria	Thermoleophilia	Thermoleophilia_unclassifi	Thermoleophilia_unclassifi	Thermoleophilia_unclassified
Otu001058	Bacteria	Actinobacteria	Nitriliruptoria	Euzebyales	Euzebyaceae	uncultured
Otu009689	Bacteria	Actinobacteria	Nitriliruptoria	Euzebyales	Euzebyaceae	uncultured
Otu000266	Bacteria	Actinobacteria	Nitriliruptoria	Euzebyales	Euzebyaceae	uncultured
Otu000823	Bacteria	Actinobacteria	Acidimicrobiia	Actinomarinales	uncultured	uncultured_ge
Otu000941	Bacteria	Actinobacteria	Acidimicrobiia	Actinomarinales	uncultured	uncultured_ge
Otu001568	Bacteria	Actinobacteria	Thermoleophilia	Gaiellales	uncultured	uncultured_ge
Otu001878	Bacteria	Actinobacteria	Thermoleophilia	Gaiellales	uncultured	uncultured_ge
Otu002892	Bacteria	Actinobacteria	Thermoleophilia	Gaiellales	uncultured	uncultured_ge
Otu003846	Bacteria	Actinobacteria	Acidimicrobiia	Microtrichales	uncultured	uncultured_ge
Otu005603	Bacteria	Actinobacteria	Thermoleophilia	Gaiellales	uncultured	uncultured_ge
Otu009979	Bacteria	Actinobacteria	Acidimicrobiia	Microtrichales	uncultured	uncultured_ge
Otu001097	Bacteria	Actinobacteria	Actinobacteria	Frankiales	uncultured	uncultured_ge
Otu001355	Bacteria	Actinobacteria	Acidimicrobiia	Microtrichales	uncultured	uncultured_ge
Otu005374	Bacteria	Armatimonadetes	Chthonomonadetes	Chthonomonadales	Chthonomonadales_fa	Chthonomonadales_ge
Otu007547	Bacteria	Armatimonadetes	uncultured	uncultured_or	uncultured_fa	uncultured_ge
Otu001012	Bacteria	Bacteria_unclassified	Bacteria_unclassified	Bacteria_unclassified	Bacteria_unclassified	Bacteria_unclassified
Otu001345	Bacteria	Bacteria_unclassified	Bacteria_unclassified	Bacteria_unclassified	Bacteria_unclassified	Bacteria_unclassified
Otu001473	Bacteria	Bacteria_unclassified	Bacteria_unclassified	Bacteria_unclassified	Bacteria_unclassified	Bacteria_unclassified
Otu001527	Bacteria	Bacteria_unclassified	Bacteria_unclassified	Bacteria_unclassified	Bacteria_unclassified	Bacteria_unclassified
Otu002122	Bacteria	Bacteria_unclassified	Bacteria_unclassified	Bacteria_unclassified	Bacteria_unclassified	Bacteria_unclassified
Otu002685	Bacteria	Bacteria_unclassified	Bacteria_unclassified	Bacteria_unclassified	Bacteria_unclassified	Bacteria_unclassified
Otu003550	Bacteria	Bacteria_unclassified	Bacteria_unclassified	Bacteria_unclassified	Bacteria_unclassified	Bacteria_unclassified
Otu003964	Bacteria	Bacteria_unclassified	Bacteria_unclassified	Bacteria_unclassified	Bacteria_unclassified	Bacteria_unclassified
Otu004329	Bacteria	Bacteria_unclassified	Bacteria_unclassified	Bacteria_unclassified	Bacteria_unclassified	Bacteria_unclassified
Otu004737	Bacteria	Bacteria_unclassified	Bacteria_unclassified	Bacteria_unclassified	Bacteria_unclassified	Bacteria_unclassified
Otu005750	Bacteria	Bacteria_unclassified	Bacteria_unclassified	Bacteria_unclassified	Bacteria_unclassified	Bacteria_unclassified
Otu006145	Bacteria	Bacteria_unclassified	Bacteria_unclassified	Bacteria_unclassified	Bacteria_unclassified	Bacteria_unclassified
Otu007451	Bacteria	Bacteria_unclassified	Bacteria_unclassified	Bacteria_unclassified	Bacteria_unclassified	Bacteria_unclassified
Otu007766	Bacteria	Bacteria_unclassified	Bacteria_unclassified	Bacteria_unclassified	Bacteria_unclassified	Bacteria_unclassified
Otu008100	Bacteria	Bacteria_unclassified	Bacteria_unclassified	Bacteria_unclassified	Bacteria_unclassified	Bacteria_unclassified
Otu010472	Bacteria	Bacteria_unclassified	Bacteria_unclassified	Bacteria_unclassified	Bacteria_unclassified	Bacteria_unclassified
Otu011986	Bacteria	Bacteria_unclassified	Bacteria_unclassified	Bacteria_unclassified	Bacteria_unclassified	Bacteria_unclassified
Otu011992	Bacteria	Bacteria_unclassified	Bacteria_unclassified	Bacteria_unclassified	Bacteria_unclassified	Bacteria_unclassified
Otu012267	Bacteria	Bacteria_unclassified	Bacteria_unclassified	Bacteria_unclassified	Bacteria_unclassified	Bacteria_unclassified
Otu012487	Bacteria	Bacteria_unclassified	Bacteria_unclassified	Bacteria_unclassified	Bacteria_unclassified	Bacteria_unclassified
Otu013061	Bacteria	Bacteria_unclassified	Bacteria_unclassified	Bacteria_unclassified	Bacteria_unclassified	Bacteria_unclassified
Otu020522	Bacteria	Bacteria_unclassified	Bacteria_unclassified	Bacteria_unclassified	Bacteria_unclassified	Bacteria_unclassified
Otu020537	Bacteria	Bacteria_unclassified	Bacteria_unclassified	Bacteria_unclassified	Bacteria_unclassified	Bacteria_unclassified
Otu027655	Bacteria	Bacteria_unclassified	Bacteria_unclassified	Bacteria_unclassified	Bacteria_unclassified	Bacteria_unclassified
Otu028075	Bacteria	Bacteria_unclassified	Bacteria_unclassified	Bacteria_unclassified	Bacteria_unclassified	Bacteria_unclassified
Otu028228	Bacteria	Bacteria_unclassified	Bacteria_unclassified	Bacteria_unclassified	Bacteria_unclassified	Bacteria_unclassified
Otu030720	Bacteria	Bacteria_unclassified	Bacteria_unclassified	Bacteria_unclassified	Bacteria_unclassified	Bacteria_unclassified
Otu012677	Bacteria	Bacteroidetes	Bacteroidia	Flavobacteriales	Flavobacteriaceae	Flavobacterium
Otu003455	Bacteria	Bacteroidetes	Ignavibacteria	Ignavibacteriales	Ignavibacteriales_unclassifi	Ignavibacteriales_unclassified
Otu008611	Bacteria	Bacteroidetes	Bacteroidia	Bacteroidales	Prevotellaceae	Prevotella
Otu003334	Bacteria	Bacteroidetes	Rhodothermia	Rhodothermales	Rhodothermaceae	Rhodothermaceae_unclassified
Otu002270	Bacteria	Bacteroidetes	Rhodothermia	Rhodothermales	Rhodothermaceae	Rhodothermaceae_unclassified
Otu002069	Bacteria	Bacteroidetes	Rhodothermia	Rhodothermales	Rhodothermaceae	Rubrivirga
Otu003116	Bacteria	Bacteroidetes	Rhodothermia	Rhodothermales	Rhodothermaceae	Rubrivirga
Otu007510	Bacteria	Bacteroidetes	Bacteroidia	Sphingobacteriales	Sphingobacteriaceae	Sphingobacteriaceae_unclassified
Otu004043	Bacteria	Calditrichaeota	Calditrichia	Calditrichales	Calditrichaceae	JdFR-76
Otu009908	Bacteria	Chlamydiae	Chlamydiae	Chlamydiales	cvE6	cvE6_ge
Otu012496	Bacteria	Chloroflexi	Chloroflexia	Kallotenuales	AKIW781	AKIW781_ge

Otu007324	Bacteria	Chloroflexi	Chloroflexia	Thermomicrobiales	AKYG1722	AKYG1722_ge
Otu001894	Bacteria	Chloroflexi	Chloroflexia	Thermomicrobiales	AKYG1722	AKYG1722_ge
Otu000193	Bacteria	Chloroflexi	Chloroflexia	Thermomicrobiales	JG30-KF-CM45	JG30-KF-CM45_ge
Otu001338	Bacteria	Chloroflexi	Chloroflexia	Thermomicrobiales	JG30-KF-CM45	JG30-KF-CM45_ge
Otu016858	Bacteria	Chloroflexi	Chloroflexia	Thermomicrobiales	JG30-KF-CM45	JG30-KF-CM45_ge
Otu015437	Bacteria	Chloroflexi	Chloroflexia	Thermomicrobiales	JG30-KF-CM45	JG30-KF-CM45_ge
Otu008302	Bacteria	Chloroflexi	JG30-KF-CM66	JG30-KF-CM66_or	JG30-KF-CM66_fa	JG30-KF-CM66_ge
Otu000251	Bacteria	Chloroflexi	KD4-96	KD4-96_or	KD4-96_fa	KD4-96_ge
Otu001551	Bacteria	Chloroflexi	KD4-96	KD4-96_or	KD4-96_fa	KD4-96_ge
Otu001965	Bacteria	Chloroflexi	KD4-96	KD4-96_or	KD4-96_fa	KD4-96_ge
Otu007438	Bacteria	Chloroflexi	KD4-96	KD4-96_or	KD4-96_fa	KD4-96_ge
Otu003818	Bacteria	Chloroflexi	Anaerolineae	RBG-13-54-9	RBG-13-54-9_fa	RBG-13-54-9_ge
Otu009317	Bacteria	Chloroflexi	Anaerolineae	RBG-13-54-9	RBG-13-54-9_fa	RBG-13-54-9_ge
Otu012796	Bacteria	Chloroflexi	Anaerolineae	RBG-13-54-9	RBG-13-54-9_fa	RBG-13-54-9_ge
Otu000453	Bacteria	Chloroflexi	Dehalococcoidia	S085	S085_fa	S085_ge
Otu014905	Bacteria	Chloroflexi	Dehalococcoidia	S085	S085_fa	S085_ge
Otu004693	Bacteria	Chloroflexi	TK10	TK10_or	TK10_fa	TK10_ge
Otu003477	Bacteria	Chloroflexi	Anaerolineae	Anaerolineales	Anaerolineaceae	uncultured
Otu005547	Bacteria	Chloroflexi	Anaerolineae	Anaerolineales	Anaerolineaceae	uncultured
Otu002934	Bacteria	Chloroflexi	Anaerolineae	Ardenticatenales	uncultured	uncultured_ge
Otu018197	Bacteria	Chloroflexi	Anaerolineae	Ardenticatenales	uncultured	uncultured_ge
Otu006508	Bacteria	Cyanobacteria	Melainabacteria	Obscuribacterales	Obscuribacterales_fa	Obscuribacterales_ge
Otu011662	Bacteria	Cyanobacteria	Melainabacteria	Obscuribacterales	Obscuribacterales_fa	Obscuribacterales_ge
Otu004051	Bacteria	Dadabacteria	Dadabacteriia	Dadabacteriales	Dadabacteriales_fa	Dadabacteriales_ge
Otu003728	Bacteria	Firmicutes	Bacilli	Lactobacillales	Aerococcaceae	Aerococcus
Otu003678	Bacteria	Firmicutes	Bacilli	Bacilli_unclassified	Bacilli_unclassified	Bacilli_unclassified
Otu006078	Bacteria	Firmicutes	Bacilli	Bacillales	Paenibacillaceae	Paenibacillus
Otu005240	Bacteria	Firmicutes	Clostridia	Clostridiales	Peptostreptococcaceae	Romboutsia
Otu003764	Bacteria	Gemmatimonadetes	AKAU4049	AKAU4049_or	AKAU4049_fa	AKAU4049_ge
Otu004531	Bacteria	Gemmatimonadetes	Gemmatimonadetes	Gemmatimonadales	Gemmatimonadaceae	Gemmatimonadaceae_unclassified
Otu017179	Bacteria	Gemmatimonadetes	Gemmatimonadetes	Gemmatimonadales	Gemmatimonadaceae	Gemmatimonadaceae_unclassified
Otu004004	Bacteria	Gemmatimonadetes	Gemmatimonadetes	Gemmatimonadales	Gemmatimonadaceae	Gemmatimonadaceae_unclassified
Otu008285	Bacteria	Gemmatimonadetes	Gemmatimonadetes	Gemmatimonadales	Gemmatimonadaceae	Gemmatimonas
Otu016384	Bacteria	Gemmatimonadetes	Longimicrobia	Longimicrobiales	Longimicrobiaceae	Longimicrobiaceae_ge
Otu000362	Bacteria	Gemmatimonadetes	Gemmatimonadetes	Gemmatimonadales	Gemmatimonadaceae	uncultured
Otu003530	Bacteria	Gemmatimonadetes	Gemmatimonadetes	Gemmatimonadales	Gemmatimonadaceae	uncultured
Otu012118	Bacteria	Gemmatimonadetes	Gemmatimonadetes	Gemmatimonadales	Gemmatimonadaceae	uncultured
Otu009753	Bacteria	Gemmatimonadetes	Gemmatimonadetes	Gemmatimonadales	Gemmatimonadaceae	uncultured
Otu001590	Bacteria	Latescibacteria	Latescibacteria	Latescibacterales	Latescibacteraceae	Latescibacteraceae_ge
Otu004702	Bacteria	Latescibacteria	Latescibacteria_cl	Latescibacteria_or	Latescibacteria_fa	Latescibacteria_ge
Otu004962	Bacteria	Latescibacteria	Latescibacteria_cl	Latescibacteria_or	Latescibacteria_fa	Latescibacteria_ge
Otu005735	Bacteria	Latescibacteria	Latescibacteria_cl	Latescibacteria_or	Latescibacteria_fa	Latescibacteria_ge
Otu025394	Bacteria	Latescibacteria	Latescibacteria_cl	Latescibacteria_or	Latescibacteria_fa	Latescibacteria_ge
Otu000201	Bacteria	Nitrospirae	Nitrospira	Nitrospirales	Nitrospiraceae	Nitrospira
Otu002286	Bacteria	Nitrospirae	Nitrospira	Nitrospirales	Nitrospiraceae	Nitrospira
Otu005354	Bacteria	Nitrospirae	Nitrospira	Nitrospirales	Nitrospiraceae	Nitrospira
Otu006285	Bacteria	Nitrospirae	Nitrospira	Nitrospirales	Nitrospiraceae	Nitrospira
Otu006632	Bacteria	Nitrospirae	Nitrospira	Nitrospirales	Nitrospiraceae	Nitrospira
Otu013386	Bacteria	Nitrospirae	Nitrospira	Nitrospirales	Nitrospiraceae	Nitrospira
Otu021171	Bacteria	Nitrospirae	Nitrospira	Nitrospirales	Nitrospiraceae	Nitrospira
Otu023681	Bacteria	Nitrospirae	Nitrospira	Nitrospirales	Nitrospiraceae	Nitrospira
Otu014507	Bacteria	Patescibacteria	Parcubacteria	Candidatus_Ryanbacteria	Candidatus_Ryanbacteria_	Candidatus_Ryanbacteria_ge
Otu004713	Bacteria	Patescibacteria	Saccharimonadia	Saccharimonadales	Saccharimonadales_fa	Saccharimonadales_ge
Otu009234	Bacteria	Patescibacteria	Saccharimonadia	Saccharimonadales	Saccharimonadales_unclas	Saccharimonadales_unclassified
Otu011401	Bacteria	Planctomycetes	Phycisphaerae	Phycisphaerales	Phycisphaeraceae	AKYG587
Otu002426	Bacteria	Planctomycetes	Phycisphaerae	CCM11a	CCM11a_fa	CCM11a_ge
Otu011795	Bacteria	Planctomycetes	Phycisphaerae	CCM11a	CCM11a_fa	CCM11a_ge
Otu002479	Bacteria	Planctomycetes	Planctomycetacia	Gemmatales	Gemmataceae	Gemmataceae_unclassified

Otu014464	Bacteria	Planctomycetes	Planctomycetacia	Gemmatales	Gemmataceae	Gemmataceae_unclassified
Otu002074	Bacteria	Planctomycetes	Planctomycetacia	Isosphaerales	Isosphaeraceae	Isosphaeraceae_unclassified
Otu003654	Bacteria	Planctomycetes	Phycisphaerae	mle1-8	mle1-8_fa	mle1-8_ge
Otu003894	Bacteria	Planctomycetes	Phycisphaerae	mle1-8	mle1-8_fa	mle1-8_ge
Otu021934	Bacteria	Planctomycetes	Phycisphaerae	mle1-8	mle1-8_fa	mle1-8_ge
Otu005446	Bacteria	Planctomycetes	Phycisphaerae	Phycisphaerales	Phycisphaeraceae	Phycisphaeraceae_unclassified
Otu004334	Bacteria	Planctomycetes	Phycisphaerae	Phycisphaerae_unclassified	Phycisphaerae_unclassified	Phycisphaerae_unclassified
Otu000606	Bacteria	Planctomycetes	Planctomycetacia	Pirellulales	Pirellulaceae	Pir4_lineage
Otu003538	Bacteria	Planctomycetes	Planctomycetacia	Pirellulales	Pirellulaceae	Pir4_lineage
Otu002813	Bacteria	Planctomycetes	Planctomycetacia	Pirellulales	Pirellulaceae	Pirellulaceae_unclassified
Otu003104	Bacteria	Planctomycetes	Pla4_lineage	Pla4_lineage_or	Pla4_lineage_fa	Pla4_lineage_ge
Otu004335	Bacteria	Planctomycetes	Planctomycetacia	Planctomycetales	Rubinisphaeraceae	SH-PL14
Otu002254	Bacteria	Planctomycetes	Planctomycetacia	Pirellulales	Pirellulaceae	uncultured
Otu002278	Bacteria	Planctomycetes	Planctomycetacia	Gemmatales	Gemmataceae	uncultured
Otu002656	Bacteria	Planctomycetes	Planctomycetacia	Gemmatales	Gemmataceae	uncultured
Otu002678	Bacteria	Planctomycetes	Planctomycetacia	Gemmatales	Gemmataceae	uncultured
Otu003020	Bacteria	Planctomycetes	Planctomycetacia	Pirellulales	Pirellulaceae	uncultured
Otu004690	Bacteria	Planctomycetes	Planctomycetacia	Gemmatales	Gemmataceae	uncultured
Otu004768	Bacteria	Planctomycetes	Planctomycetacia	Gemmatales	Gemmataceae	uncultured
Otu007538	Bacteria	Planctomycetes	Planctomycetacia	Pirellulales	Pirellulaceae	uncultured
Otu010332	Bacteria	Planctomycetes	Planctomycetacia	Gemmatales	Gemmataceae	uncultured
Otu008584	Bacteria	Planctomycetes	Planctomycetacia	Planctomycetales	Gimesiaceae	uncultured
Otu005432	Bacteria	Planctomycetes	Planctomycetacia	Gemmatales	Gemmataceae	uncultured
Otu008953	Bacteria	Planctomycetes	Planctomycetacia	Gemmatales	Gemmataceae	uncultured
Otu002551	Bacteria	Planctomycetes	Planctomycetacia	Gemmatales	Gemmataceae	uncultured
Otu003053	Bacteria	Planctomycetes	Planctomycetacia	Gemmatales	Gemmataceae	uncultured
Otu007472	Bacteria	Planctomycetes	Planctomycetacia	Gemmatales	Gemmataceae	uncultured
Otu006585	Bacteria	Planctomycetes	Planctomycetacia	Gemmatales	Gemmataceae	uncultured
Otu008984	Bacteria	Planctomycetes	Planctomycetacia	Gemmatales	Gemmataceae	uncultured
Otu003178	Bacteria	Planctomycetes	Planctomycetacia	Planctomycetales	uncultured	uncultured_ge
Otu003450	Bacteria	Planctomycetes	Phycisphaerae	Tepidisphaerales	WD2101_soil_group	WD2101_soil_group_ge
Otu001936	Bacteria	Proteobacteria	Alphaproteobacteria	Rhizobiales	A0839	A0839_ge
Otu003916	Bacteria	Proteobacteria	Alphaproteobacteria	Acetobacterales	Acetobacteraceae	Acetobacteraceae_unclassified
Otu004163	Bacteria	Proteobacteria	Alphaproteobacteria	Acetobacterales	Acetobacteraceae	Acetobacteraceae_unclassified
Otu003210	Bacteria	Proteobacteria	Alphaproteobacteria	Alphaproteobacteria_uncla	Alphaproteobacteria_uncla	Alphaproteobacteria_unclassified
Otu003245	Bacteria	Proteobacteria	Alphaproteobacteria	Alphaproteobacteria_uncla	Alphaproteobacteria_uncla	Alphaproteobacteria_unclassified
Otu003134	Bacteria	Proteobacteria	Alphaproteobacteria	Sphingomonadales	Sphingomonadaceae	Altererythrobacter
Otu002265	Bacteria	Proteobacteria	Alphaproteobacteria	Caulobacterales	Parvularculaceae	Amphiplicatus
Otu012494	Bacteria	Proteobacteria	Gammaproteobacteria	Betaproteobacteriales	B1-7BS	B1-7BS_ge
Otu001376	Bacteria	Proteobacteria	Deltaproteobacteria	Myxococcales	bacteriap25	bacteriap25_ge
Otu005727	Bacteria	Proteobacteria	Alphaproteobacteria	Rhizobiales	Rhizobiales_Incertae_Sedis	Bauldia
Otu003738	Bacteria	Proteobacteria	Gammaproteobacteria	Betaproteobacteriales	Betaproteobacteriales_unc	Betaproteobacteriales_unclassified
Otu012171	Bacteria	Proteobacteria	Gammaproteobacteria	Betaproteobacteriales	Betaproteobacteriales_unc	Betaproteobacteriales_unclassified
Otu009862	Bacteria	Proteobacteria	Deltaproteobacteria	Myxococcales	Blfdi19	Blfdi19_ge
Otu002936	Bacteria	Proteobacteria	Gammaproteobacteria	Betaproteobacteriales	Burkholderiaceae	Burkholderiaceae_unclassified
Otu012840	Bacteria	Proteobacteria	Gammaproteobacteria	Betaproteobacteriales	Burkholderiaceae	Burkholderiaceae_unclassified
Otu013497	Bacteria	Proteobacteria	Gammaproteobacteria	Betaproteobacteriales	Burkholderiaceae	Burkholderiaceae_unclassified
Otu016062	Bacteria	Proteobacteria	Gammaproteobacteria	Betaproteobacteriales	Burkholderiaceae	Burkholderiaceae_unclassified
Otu024140	Bacteria	Proteobacteria	Gammaproteobacteria	Betaproteobacteriales	Burkholderiaceae	Burkholderiaceae_unclassified
Otu021727	Bacteria	Proteobacteria	Gammaproteobacteria	Betaproteobacteriales	Burkholderiaceae	Burkholderiaceae_unclassified
Otu006380	Bacteria	Proteobacteria	Alphaproteobacteria	Caulobacterales	Caulobacteraceae	Caulobacteraceae_unclassified
Otu007867	Bacteria	Proteobacteria	Alphaproteobacteria	Caulobacterales	Caulobacteraceae	Caulobacteraceae_unclassified
Otu009022	Bacteria	Proteobacteria	Gammaproteobacteria	CCM19a	CCM19a_fa	CCM19a_ge
Otu000836	Bacteria	Proteobacteria	Deltaproteobacteria	Deltaproteobacteria_uncla	Deltaproteobacteria_uncla	Deltaproteobacteria_unclassified
Otu001640	Bacteria	Proteobacteria	Deltaproteobacteria	Deltaproteobacteria_uncla	Deltaproteobacteria_uncla	Deltaproteobacteria_unclassified
Otu008979	Bacteria	Proteobacteria	Deltaproteobacteria	Deltaproteobacteria_uncla	Deltaproteobacteria_uncla	Deltaproteobacteria_unclassified
Otu033768	Bacteria	Proteobacteria	Deltaproteobacteria	Deltaproteobacteria_uncla	Deltaproteobacteria_uncla	Deltaproteobacteria_unclassified
Otu001460	Bacteria	Proteobacteria	Alphaproteobacteria	Sphingomonadales	Sphingomonadaceae	Ellin6055

Otu002926	Bacteria	Proteobacteria	Gammaproteobacteria	Enterobacteriales	Enterobacteriaceae	Enterobacteriaceae_unclassified
Otu003074	Bacteria	Proteobacteria	Gammaproteobacteria	Enterobacteriales	Enterobacteriaceae	Enterobacteriaceae_unclassified
Otu006773	Bacteria	Proteobacteria	Gammaproteobacteria	Enterobacteriales	Enterobacteriaceae	Enterobacteriaceae_unclassified
Otu010106	Bacteria	Proteobacteria	Gammaproteobacteria	Enterobacteriales	Enterobacteriaceae	Enterobacteriaceae_unclassified
Otu013536	Bacteria	Proteobacteria	Gammaproteobacteria	Enterobacteriales	Enterobacteriaceae	Enterobacteriaceae_unclassified
Otu013618	Bacteria	Proteobacteria	Gammaproteobacteria	Enterobacteriales	Enterobacteriaceae	Enterobacteriaceae_unclassified
Otu019713	Bacteria	Proteobacteria	Gammaproteobacteria	Enterobacteriales	Enterobacteriaceae	Enterobacteriaceae_unclassified
Otu020311	Bacteria	Proteobacteria	Gammaproteobacteria	Enterobacteriales	Enterobacteriaceae	Enterobacteriaceae_unclassified
Otu020381	Bacteria	Proteobacteria	Gammaproteobacteria	Enterobacteriales	Enterobacteriaceae	Enterobacteriaceae_unclassified
Otu021286	Bacteria	Proteobacteria	Gammaproteobacteria	Enterobacteriales	Enterobacteriaceae	Enterobacteriaceae_unclassified
Otu033370	Bacteria	Proteobacteria	Gammaproteobacteria	Enterobacteriales	Enterobacteriaceae	Enterobacteriaceae_unclassified
Otu023417	Bacteria	Proteobacteria	Gammaproteobacteria	Enterobacteriales	Enterobacteriaceae	Enterobacteriaceae_unclassified
Otu003320	Bacteria	Proteobacteria	Gammaproteobacteria	Enterobacteriales	Enterobacteriaceae	Enterobacteriaceae_unclassified
Otu019890	Bacteria	Proteobacteria	Gammaproteobacteria	Enterobacteriales	Enterobacteriaceae	Escherichia-Shigella
Otu030657	Bacteria	Proteobacteria	Gammaproteobacteria	Enterobacteriales	Enterobacteriaceae	Escherichia-Shigella
Otu000310	Bacteria	Proteobacteria	Gammaproteobacteria	Gammaproteobacteria_un	Gammaproteobacteria_unc	Gammaproteobacteria_unclassified
Otu000524	Bacteria	Proteobacteria	Gammaproteobacteria	Gammaproteobacteria_un	Gammaproteobacteria_unc	Gammaproteobacteria_unclassified
Otu001150	Bacteria	Proteobacteria	Gammaproteobacteria	Gammaproteobacteria_un	Gammaproteobacteria_unc	Gammaproteobacteria_unclassified
Otu002191	Bacteria	Proteobacteria	Gammaproteobacteria	Gammaproteobacteria_un	Gammaproteobacteria_unc	Gammaproteobacteria_unclassified
Otu006579	Bacteria	Proteobacteria	Gammaproteobacteria	Gammaproteobacteria_un	Gammaproteobacteria_unc	Gammaproteobacteria_unclassified
Otu006940	Bacteria	Proteobacteria	Gammaproteobacteria	Gammaproteobacteria_un	Gammaproteobacteria_unc	Gammaproteobacteria_unclassified
Otu007571	Bacteria	Proteobacteria	Gammaproteobacteria	Gammaproteobacteria_un	Gammaproteobacteria_unc	Gammaproteobacteria_unclassified
Otu009296	Bacteria	Proteobacteria	Gammaproteobacteria	Gammaproteobacteria_un	Gammaproteobacteria_unc	Gammaproteobacteria_unclassified
Otu014317	Bacteria	Proteobacteria	Gammaproteobacteria	Gammaproteobacteria_un	Gammaproteobacteria_unc	Gammaproteobacteria_unclassified
Otu016879	Bacteria	Proteobacteria	Gammaproteobacteria	Gammaproteobacteria_un	Gammaproteobacteria_unc	Gammaproteobacteria_unclassified
Otu019205	Bacteria	Proteobacteria	Gammaproteobacteria	Gammaproteobacteria_un	Gammaproteobacteria_unc	Gammaproteobacteria_unclassified
Otu002978	Bacteria	Proteobacteria	Alphaproteobacteria	Rhizobiales	Hyphomicrobiaceae	Hyphomicrobiaceae_unclassified
Otu007047	Bacteria	Proteobacteria	Alphaproteobacteria	Rhizobiales	Hyphomicrobiaceae	Hyphomicrobium
Otu005434	Bacteria	Proteobacteria	Gammaproteobacteria	KF-JG30-C25	KF-JG30-C25_fa	KF-JG30-C25_ge
Otu000613	Bacteria	Proteobacteria	Gammaproteobacteria	Xanthomonadales	Rhodanobacteraceae	Luteibacter
Otu000785	Bacteria	Proteobacteria	Gammaproteobacteria	Xanthomonadales	Xanthomonadaceae	Lysobacter
Otu002554	Bacteria	Proteobacteria	Gammaproteobacteria	Betaproteobacteriales	Methylophilaceae	Methylobacillus
Otu003151	Bacteria	Proteobacteria	Gammaproteobacteria	Methylococcales	Methylococcales_unclassifi	Methylococcales_unclassified
Otu000455	Bacteria	Proteobacteria	Alphaproteobacteria	Rhizobiales	Methyloligellaceae	Methyloligellaceae_unclassified
Otu020007	Bacteria	Proteobacteria	Gammaproteobacteria	Betaproteobacteriales	Methylophilaceae	Methylophilaceae_unclassified
Otu002886	Bacteria	Proteobacteria	Gammaproteobacteria	Betaproteobacteriales	Nitrosomonadaceae	mle1-7
Otu000817	Bacteria	Proteobacteria	Deltaproteobacteria	Myxococcales	Myxococcales_unclassified	Myxococcales_unclassified
Otu006987	Bacteria	Proteobacteria	Deltaproteobacteria	NB1-j	NB1-j_fa	NB1-j_ge
Otu013985	Bacteria	Proteobacteria	Gammaproteobacteria	Nitrosococcales	Nitrosococcaceae	Nitrosococcaceae_unclassified
Otu008375	Bacteria	Proteobacteria	Deltaproteobacteria	Bdellovibrionales	Bdellovibrionaceae	OM27_clade
Otu005178	Bacteria	Proteobacteria	Alphaproteobacteria	Rhizobiales	Hyphomicrobiaceae	Pedomicrobium
Otu000428	Bacteria	Proteobacteria	Deltaproteobacteria	Myxococcales	Polyangiaceae	Polyangiaceae_unclassified
Otu004699	Bacteria	Proteobacteria	Gammaproteobacteria	Salinisphaerales	Solimonadaceae	Polycyclovorans
Otu022008	Bacteria	Proteobacteria	Gammaproteobacteria	Salinisphaerales	Solimonadaceae	Polycyclovorans
Otu008301	Bacteria	Proteobacteria	Proteobacteria_unclassifi	Proteobacteria_unclassifie	Proteobacteria_unclassifiec	Proteobacteria_unclassified
Otu014964	Bacteria	Proteobacteria	Proteobacteria_unclassifi	Proteobacteria_unclassifie	Proteobacteria_unclassifiec	Proteobacteria_unclassified
Otu020439	Bacteria	Proteobacteria	Proteobacteria_unclassifi	Proteobacteria_unclassifie	Proteobacteria_unclassifiec	Proteobacteria_unclassified
Otu023420	Bacteria	Proteobacteria	Proteobacteria_unclassifi	Proteobacteria_unclassifie	Proteobacteria_unclassifiec	Proteobacteria_unclassified
Otu025699	Bacteria	Proteobacteria	Proteobacteria_unclassifi	Proteobacteria_unclassifie	Proteobacteria_unclassifiec	Proteobacteria_unclassified
Otu002066	Bacteria	Proteobacteria	Gammaproteobacteria	Xanthomonadales	Rhodanobacteraceae	Pseudofulvimonas
Otu008994	Bacteria	Proteobacteria	Alphaproteobacteria	Rhizobiales	Rhizobiaceae	Rhizobiaceae_unclassified
Otu001091	Bacteria	Proteobacteria	Alphaproteobacteria	Rhizobiales	Rhizobiales_Incertae_Sedis	Rhizobiales_Incertae_Sedis_unclassified
Otu004061	Bacteria	Proteobacteria	Alphaproteobacteria	Rhizobiales	Rhizobiales_unclassified	Rhizobiales_unclassified
Otu004251	Bacteria	Proteobacteria	Alphaproteobacteria	Rhizobiales	Rhizobiales_unclassified	Rhizobiales_unclassified
Otu005635	Bacteria	Proteobacteria	Alphaproteobacteria	Rhizobiales	Rhizobiales_unclassified	Rhizobiales_unclassified
Otu002336	Bacteria	Proteobacteria	Alphaproteobacteria	Rhizobiales	Rhizobiales_unclassified	Rhizobiales_unclassified
Otu004083	Bacteria	Proteobacteria	Alphaproteobacteria	Rhodobacterales	Rhodobacteraceae	Rhodobacteraceae_unclassified
Otu001235	Bacteria	Proteobacteria	Alphaproteobacteria	Azospirillales	Azospirillaceae	Skermanella

Otu018633	Bacteria	Proteobacteria	Alphaproteobacteria	Azospirillales	Azospirillaceae	Skermanella
Otu006835	Bacteria	Proteobacteria	Alphaproteobacteria	Sphingomonadales	Sphingomonadaceae	Sphingomonadaceae_unclassified
Otu001078	Bacteria	Proteobacteria	Alphaproteobacteria	Sphingomonadales	Sphingomonadaceae	Sphingomonas
Otu002979	Bacteria	Proteobacteria	Gammaproteobacteria	Xanthomonadales	Xanthomonadaceae	Stenotrophomonas
Otu001493	Bacteria	Proteobacteria	Deltaproteobacteria	Desulfarculales	Desulfarculaceae	uncultured
Otu002792	Bacteria	Proteobacteria	Deltaproteobacteria	Myxococcales	Sandaracinaceae	uncultured
Otu003683	Bacteria	Proteobacteria	Alphaproteobacteria	Micropepsales	Micropepsaceae	uncultured
Otu006240	Bacteria	Proteobacteria	Deltaproteobacteria	Desulfarculales	Desulfarculaceae	uncultured
Otu000728	Bacteria	Proteobacteria	Alphaproteobacteria	Rhizobiales	uncultured	uncultured_ge
Otu002862	Bacteria	Proteobacteria	Alphaproteobacteria	Rhizobiales	uncultured	uncultured_ge
Otu006745	Bacteria	Proteobacteria	Gammaproteobacteria	Nitrosococcales	Nitrosococcaceae	wb1-P19
Otu025721	Bacteria	Proteobacteria	Gammaproteobacteria	Nitrosococcales	Nitrosococcaceae	wb1-P19
Otu003635	Bacteria	Proteobacteria	Gammaproteobacteria	Nitrosococcales	Nitrosococcaceae	wb1-P19
Otu005013	Bacteria	Proteobacteria	Gammaproteobacteria	Nitrosococcales	Nitrosococcaceae	wb1-P19
Otu012302	Bacteria	Proteobacteria	Gammaproteobacteria	Xanthomonadales	Xanthomonadaceae	Xanthomonadaceae_unclassified
Otu005994	Bacteria	Proteobacteria	Gammaproteobacteria	Xanthomonadales	Xanthomonadaceae	Xanthomonadaceae_unclassified
Otu006098	Bacteria	Proteobacteria	Gammaproteobacteria	Xanthomonadales	Xanthomonadaceae	Xanthomonadaceae_unclassified
Otu010061	Bacteria	Proteobacteria	Gammaproteobacteria	Xanthomonadales	Xanthomonadales_unclassi	Xanthomonadales_unclassified
Otu004166	Bacteria	Proteobacteria	Gammaproteobacteria	Xanthomonadales	Xanthomonadales_unclassi	Xanthomonadales_unclassified
Otu003610	Bacteria	Rokubacteria	NC10	Rokubacteriales	Rokubacteriales_fa	Rokubacteriales_ge
Otu007537	Bacteria	Rokubacteria	NC10	Rokubacteriales	Rokubacteriales_fa	Rokubacteriales_ge
Otu001669	Bacteria	Verrucomicrobia	Verrucomicrobiae	Chthoniobacterales	Chthoniobacteraceae	Candidatus_Udaeobacter
Otu007528	Bacteria	Verrucomicrobia	Verrucomicrobiae	Opitutales	Opitutaceae	Opitutaceae_unclassified
Otu006051	Bacteria	Verrucomicrobia	Verrucomicrobiae	Pedosphaerales	Pedosphaeraceae	Pedosphaeraceae_ge
Otu009641	Bacteria	Verrucomicrobia	Verrucomicrobiae	Pedosphaerales	Pedosphaeraceae	Pedosphaeraceae_ge
Otu014325	Bacteria	Verrucomicrobia	Verrucomicrobiae	Pedosphaerales	Pedosphaeraceae	Pedosphaeraceae_ge

P. destructans positive Indicator taxa

OTU	Kingdo	Phylum				
Otu000321	Bacteria	Actinobacteria	Actinobacteria	Micrococcales	Micrococcaceae	Micrococcaceae_unclassified
Otu000339	Bacteria	Actinobacteria	Actinobacteria	Micrococcales	Micrococcaceae	Micrococcaceae_unclassified
Otu000640	Bacteria	Actinobacteria	Actinobacteria	Micrococcales	Micrococcaceae	Micrococcaceae_unclassified
Otu000161	Bacteria	Actinobacteria	Actinobacteria	Actinobacteria_unclassifie	Actinobacteria_unclassifie	Actinobacteria_unclassified
Otu001641	Bacteria	Proteobacteria	Gammaproteobacteria	Enterobacteriales	Enterobacteriaceae	Enterobacteriaceae_unclassified
Otu002065	Bacteria	Actinobacteria	Actinobacteria	Micrococcales	Micrococcaceae	Micrococcaceae_unclassified
Otu001303	Bacteria	Actinobacteria	Actinobacteria	Micrococcales	Micrococcaceae	Micrococcaceae_unclassified
Otu002167	Bacteria	Actinobacteria	Actinobacteria	Micrococcales	Micrococcaceae	Micrococcaceae_unclassified
Otu001271	Bacteria	Actinobacteria	Actinobacteria	Micrococcales	Micrococcaceae	Micrococcaceae_unclassified
Otu000693	Bacteria	Actinobacteria	Actinobacteria	Micrococcales	Micrococcaceae	Micrococcaceae_unclassified
Otu001840	Bacteria	Actinobacteria	Actinobacteria	Micrococcales	Micrococcaceae	Micrococcaceae_unclassified
Otu000849	Bacteria	Proteobacteria	Gammaproteobacteria	Gammaproteobacteria_un	Gammaproteobacteria_unc	Gammaproteobacteria_unclassified
Otu000505	Bacteria	Actinobacteria	Actinobacteria	Micrococcales	Micrococcaceae	Micrococcaceae_unclassified
Otu002001	Bacteria	Actinobacteria	Actinobacteria	Micrococcales	Micrococcaceae	Micrococcaceae_unclassified
